# Efficacy and safety of bofanglutide, a GLP-1 receptor agonist, in Chinese adults with overweight or obesity: a randomized, double-blind, placebo-controlled phase 2b trial

**DOI:** 10.1038/s41392-026-02586-8

**Published:** 2026-02-27

**Authors:** Linong Ji, Leili Gao, Junhang Tian, Ruihua Dong, Zhongtao Zhang, Hongyan Shu, Jing Zhao, Liyuan Zhao, Anshun He, Tian Xie, Yue Li, Wei Chen

**Affiliations:** 1 Peking University People’s Hospital, Peking University Diabetes Center, Beijing, China; 2 Luoyang Third People’s Hospital, Luoyang, Henan China; 3 Beijing Friendship Hospital, Capital Medical University, Beijing, China; 4 Zibo Municipal Hospital, Zibo, Shandong China; 5 Gan & Lee Pharmaceuticals, Beijing, China; 6 Gan & Lee Pharmaceuticals Shandong Co. Ltd, Linyi, Shandong China

**Keywords:** Endocrine system and metabolic diseases, Biologics

## Abstract

Bofanglutide is a novel biweekly (once every two weeks; Q2W) glucagon-like peptide-1 receptor agonist. We evaluated the efficacy and safety of bofanglutide in Chinese adults with overweight or obesity in a randomized, double-blind, placebo-controlled phase 2b trial (ClinicalTrials.gov, NCT06256562). Adults with overweight (body mass index [BMI] ≥24, <28 kg/m^2^) and at least one weight-related comorbidity, or obesity (BMI ≥ 28 kg/m^2^), were randomly assigned to five dose groups: 12 mg Q2W, 18 mg Q2W, 24 mg Q2W, 48 mg Q2W, and 24 mg once weekly (QW), with randomization to bofanglutide or placebo within each dose group. The primary endpoint was the percentage change in body weight from baseline to week 30. Between June 8, 2023, and June 5, 2024, 340 participants (185 [54.4%] male; mean age, 33.1 years; mean body weight, 95.6 kg; mean BMI, 33.2 kg/m²) were randomized into the following groups: bofanglutide 12 mg Q2W (*n* = 52), 18 mg Q2W (*n* = 53), 24 mg Q2W (*n* = 52), 48 mg Q2W (*n* = 64), 24 mg QW (*n* = 53), or placebo (*n* = 66). Overall, 286 participants (84.1%) completed the trial. The mean percentage change in body weight from baseline to week 30 ranged from −9.75% to −16.69% with bofanglutide, compared with −1.15% with placebo (all *p* < 0.001 versus placebo). Adverse events occurred in 98.9% (271/274) of the bofanglutide group versus 86.4% (57/66) of the placebo group and were mostly grade 1–2 gastrointestinal events (83.9% [230/274] with bofanglutide and 33.3% [22/66] with placebo). Bofanglutide is generally well tolerated and has a robust ability to reduce body weight.

## Introduction

Obesity is a chronic, relapsing, and progressive disease that poses a significant global public health challenge. Its pathogenesis reflects a complex interplay of genetic, metabolic, behavioral, and environmental factors, ultimately resulting in dysregulated energy balance.^1,2^ Since 1975, the prevalence of obesity has increased dramatically worldwide. According to recent data from the World Health Organization (WHO), more than 2.5 billion adults aged 18 years and older are overweight, including more than 890 million individuals living with obesity.^3^ China has mirrored this global trend, with a rapid rise in obesity prevalence.^4,5^ A large-scale cross-sectional survey involving 15.8 million Chinese adults reported that 34.8% were classified as overweight and 14.1% had obesity.^4^ The clinical consequences of obesity are substantial, as it is a well-recognized risk factor for a spectrum of complications including insulin resistance, type 2 diabetes mellitus (T2DM), dyslipidemia, hypertension, cardiovascular disease, metabolic dysfunction-associated steatotic liver disease, and osteoarthritis.^6–8^ Consequently, there is an urgent and growing need for effective and sustainable interventions to manage this disease and its associated complications.^9,10^

The cornerstone of weight management remains comprehensive lifestyle intervention, including dietary modification, increased physical activity, and behavioral therapy.^11^ Although lifestyle intervention is recommended as the first-line treatment for overweight and obesity, achieving and maintaining clinically meaningful weight loss through this intervention alone is often challenging.^12^ For a substantial proportion of patients, particularly those who have previously failed to lose weight or maintain weight loss, the use of weight-loss medication may be considered.^13^ Over the past decade, substantial advancements have been made in the pharmacological management of obesity, particularly with the development of glucagon-like peptide-1 (GLP-1) receptor agonists (RAs). These agents have demonstrated remarkable efficacy in clinical trials to reduce body weight, and have been associated with a wide range of beneficial pleiotropic effects, including improved glycemic control, blood pressure reduction, and favorable lipid profile changes.^10,14–16^ However, real-world observational studies have consistently reported lower effectiveness of GLP-1 RAs compared with outcomes observed in prospective controlled clinical trials.^17^ One plausible explanation for this discrepancy may be attributed to insufficient medication adherence and treatment persistence. In a survey investigating the reasons for discontinuation of GLP-1 RAs among 637 physicians and patients treated with these agents, the most common patient-reported problem with the treatment was not preferring injections.^18^ These findings indicate that there is an unmet need for effective weight-loss therapies with less frequent dosing frequency (e.g., biweekly or monthly), which may enhance treatment adherence and persistence and thereby ensure the delivery of the long-term benefits associated with sustained weight loss.^19^

Bofanglutide (also known as GZR18) is a structurally modified human GLP-1 analog that does not contain unnatural amino acids and incorporates a 22C fatty diacid moiety.^20^ The pharmacokinetics of bofanglutide include a half-life of approximately 1 week and slower, more gradual absorption from subcutaneous tissue.^20,21^ These characteristics are expected to permit a longer two-week interval between administrations, which enables bofanglutide to be developed as a potential biweekly treatment option for weight management and T2DM. The unique molecular design of bofanglutide confers sustained yet moderate bioactivity, which is anticipated to attenuate the dose-related gastrointestinal adverse events commonly observed with more potent, short-acting GLP-1 RAs.^20,22^ Improved tolerability may, in turn, allow titration to higher therapeutic doses, achieving substantial efficacy while maintaining good safety and tolerability profile. In a 35-week phase 2a clinical trial in Chinese adults with obesity, bofanglutide resulted in a mean placebo-adjusted body weight reduction of up to 18.6% at doses escalated to 30 mg, with a favorable safety profile.^23^ Notably, within the trial, 38.5% of the bofanglutide-treated participants received bofanglutide biweekly and achieved a 13.5% reduction in body weight at week 35. These results provide preliminary evidence to support the further development of bofanglutide as an effective biweekly GLP-1 analog.

To further rigorously investigate the feasibility of biweekly dosing frequency and the weight-loss potential at higher target doses, a 30-week, randomized, double-blind, placebo-controlled phase 2b trial was conducted in Chinese adults with obesity or overweight and at least one weight-related comorbidity. The trial was designed with a dose-escalation period (10–18 weeks) followed by a dose-maintenance period (12–20 weeks) at the respective target doses. To our knowledge, this is the first phase 2 trial specifically designed to explore the dose-exposure relationship of a biweekly GLP-1 analog for weight management and to directly compare weekly versus biweekly dosing regimens. By targeting injection frequency, a key determinant of treatment adherence and persistence, this study provides critical evidence on whether this extended dosing interval can sustain clinically meaningful weight loss, improve cardiometabolic outcomes, and maintain an acceptable safety and tolerability profile. These findings have important clinical implications, as they may establish a more convenient therapeutic option capable of improving long-term adherence and clinical outcomes in the management of obesity.

## Results

### Demographic and baseline characteristics

**Fig. 1:**
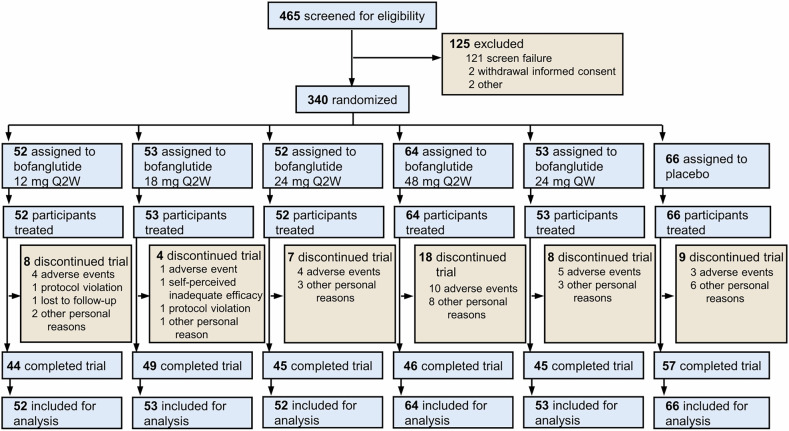
Flow diagram of the participants. The flow diagram illustrates the disposition of the participants screened and enrolled. QW once weekly, Q2W biweekly

Between June 8, 2023, and June 5, 2024, 465 individuals were screened, and 340 of them were randomized into one of the following groups: bofanglutide 12 mg Q2W (*n* = 52), 18 mg Q2W (*n* = 53), 24 mg Q2W (*n* = 52), 48 mg Q2W (*n* = 64), 24 mg QW (*n* = 53), or placebo (*n* = 66). Overall, 286 participants (84.1%) completed the trial, while 45 (16.4%) of the 274 participants receiving bofanglutide and 9 (13.6%) of the 66 participants receiving placebo prematurely discontinued the trial (Fig. 1). The proportion of participants who completed the trial was similar across treatment groups (range 85%–92%), except for the bofanglutide 48 mg Q2W group, which had fewer completers (72%). All 340 randomized participants were included in both the modified intention-to-treat (mITT) and safety populations. Demographic and baseline characteristics were well balanced across the treatment groups (Table 1). Overall, the participants had a mean age of 33.1 (standard deviation [SD] 7.2) years, a mean baseline body weight of 95.6 (19.7) kg, and a mean baseline BMI of 33.2 (4.6) kg/m². Among the 340 randomized participants, 185 (54.4%) were male and 155 (45.6%) were female, and detailed sex‑specific baseline characteristics are provided in supplementary Table 1. At baseline, female participants demonstrated numerically lower body weights, BMIs, lipid profiles, and blood pressures than male participants. Additionally, 313 participants (92.1%) had a BMI of 28 kg/m² or more, and 334 participants (98.2%) had weight-related comorbidities, among whom 79 participants (23.7%) had prediabetes. Among the participants, 332 (97.6%) had medication adherence within the 80–120% range.

**Table 1:** Demographic and baseline characteristics

	Bofanglutide Q2W				Bofanglutide QW 24 mg (*N* = 53)	Placebo (*N* = 66)
	12 mg (*N* = 52)	18 mg (*N* = 53)	24 mg (*N* = 52)	48 mg (*N* = 64)		
Age, years	32.3 (7.0)	33.0 (7.6)	33.7 (6.3)	32.8 (7.8)	32.8 (7.4)	33.7 (7.2)
Sex [n (%)]						
Male	32 (61.5)	27 (50.9)	32 (61.5)	29 (45.3)	30 (56.6)	35 (53.0)
Female	20 (38.5)	26 (49.1)	20 (38.5)	35 (54.7)	23 (43.4)	31 (47.0)
Race, Asian [*n* (%)]	52 (100)	53 (100)	52 (100)	64 (100)	53 (100)	66 (100)
Body weight (kg)	97.22 (21.22)	93.01 (14.59)	97.17 (17.22)	94.87 (23.17)	94.52 (18.60)	96.90 (21.46)
BMI (kg/m^2^)	33.7 (4.9)	32.4 (4.1)	33.3 (4.1)	33.0 (4.9)	33.1 (4.5)	33.8 (5.0)
BMI (kg/m^2^) [*n* (%)]						
≥24, < 28	1 (1.9)	5 (9.4)	3 (5.8)	8 (12.5)	5 (9.4)	5 (7.6)
≥28	51 (98.1)	48 (90.6)	49 (94.2)	56 (87.5)	48 (90.6)	61 (92.4)
Waist circumference (cm)	107.7 (13.2)	105.1 (11.2)	108.1 (11.0)	105.5 (14.0)	106.3 (12.2)	108.2 (13.1)
HbA1c (%)	5.60 (0.39)	5.48 (0.41)	5.57 (0.42)	5.54 (0.34)	5.67 (0.49)	5.62 (0.37)
Triglycerides (mmol/L)	1.99 (0.96)	2.00 (1.04)	2.00 (0.76)	1.67 (0.83)	2.16 (1.14)	1.73 (0.88)
Total cholesterol (mmol/L)	4.86 (0.80)	4.95 (0.74)	5.08 (0.89)	4.94 (1.12)	5.08 (0.95)	4.82 (0.87)
LDL cholesterol (mmol/L)	3.13 (0.75)	3.10 (0.63)	3.29 (0.80)	3.09 (0.75)	3.20 (0.79)	3.10 (0.77)
HDL cholesterol (mmol/L)	1.08 (0.23)	1.13 (0.25)	1.14 (0.17)	1.22 (0.24)	1.11 (0.24)	1.15 (0.24)
SBP (mmHg)	122.5 (9.60)	121.5 (11.41)	122.9 (10.53)	123.4 (10.37)	119.9 (11.44)	119.7 (10.31)
DBP (mmHg)	81.83 (7.42)	81.16 (9.02)	81.40 (7.71)	82.11 (8.30)	81.46 (7.63)	80.36 (7.44)
Comorbidities occurring in ≥ 10% of participants in total, *n* (%)						
Hepatic steatosis^1^	51 (98.1)	50 (94.3)	49 (94.2)	57 (89.1)	50 (94.3)	59 (89.4)
Hyperlipidemia	26 (50.0)	25 (47.2)	27 (51.9)	21 (32.8)	28 (52.8)	29 (43.9)
Hyperuricemia	20 (38.5)	24 (45.3)	23 (44.2)	20 (31.3)	22 (41.5)	23 (34.8)
Impaired glucose tolerance	16 (30.8)	10 (18.9)	7 (13.5)	15 (23.4)	13 (24.5)	15 (22.7)
Dyslipidemia	7 (13.5)	5 (9.4)	11 (21.2)	17 (26.6)	10 (18.9)	10 (15.2)
Hyperinsulinemia	9 (17.3)	4 (7.5)	8 (15.4)	9 (14.1)	8 (15.1)	6 (9.1)
Hypertension	8 (15.4)	13 (24.5)	11 (21.2)	11 (17.2)	9 (17.0)	9 (13.6)

^1^Hepatic steatosis was diagnosed by ultrasound

Data are presented as the means (SDs) or n (%), mITT population. *BMI* body mass index, *HbA1c* glycated hemoglobin, *HDL* high-density lipoprotein, *LDL* low-density lipoprotein, *SBP* systolic blood pressure, *DBP* diastolic blood pressure, *mITT* modified intention-to-treat, *SD* standard deviation, *QW* once weekly, *Q2W* biweekly

### Primary endpoint

**Fig. 2:**
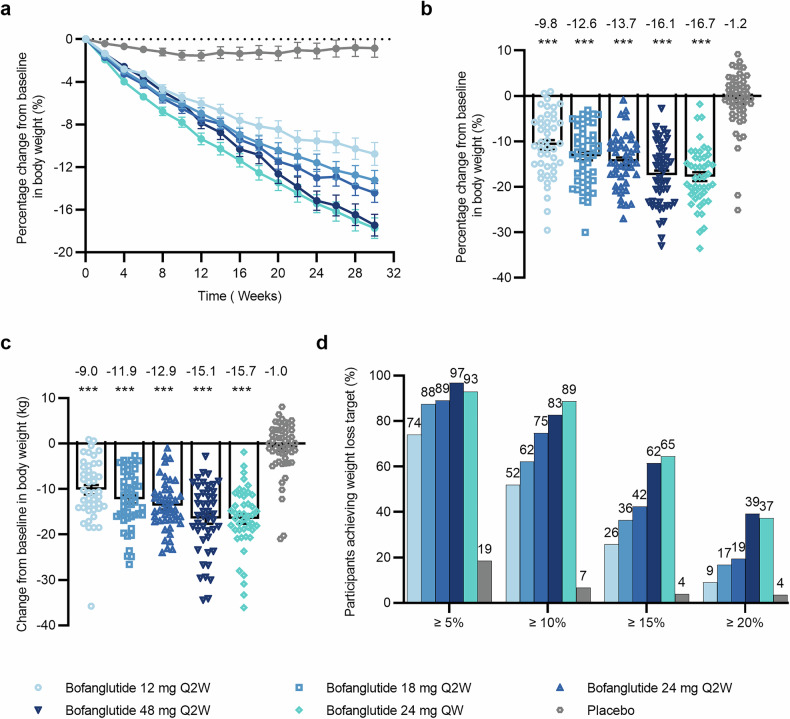
Changes in body weight with bofanglutide compared with placebo. **a** Percentage change from baseline in body weight over time. **b** Percentage change from baseline in body weight at week 30. **c** Change from baseline in body weight at week 30. **d** The proportions of participants who reached weight loss targets (≥5%, ≥10%, ≥15%, and ≥20%). Data in (**a**, **b**, **c**) are represented as the LSM (SE) from ANCOVA with multiple imputation in the mITT population. Proportion of participants who reached weight loss targets was obtained by dividing the number of participants reaching the respective target at week 30 by the number of participants in the mITT population. For (**b**, **c**) a statistically significant difference between the bofanglutide and placebo groups at the end of treatment is indicated as ***p < 0.001. Bofanglutide Q2W: 12 mg, *n* = 52; 18 mg, *n* = 53; 24 mg, *n* = 52; 48 mg, *n* = 64; bofanglutide 24 mg QW: *n* = 53; placebo: *n* = 66. ANCOVA analysis of covariance, LSM least squares mean, SE standard error, mITT modified intention-to-treat, QW once weekly, Q2W biweekly

In the primary analysis, the body weight reduction from baseline to week 30 was greater with bofanglutide than with placebo (Fig. 2a, b; Table 2). The least squares mean (LSM) percentage changes for participants receiving bofanglutide 12 mg, 18 mg, 24 mg, and 48 mg Q2W were −9.75% (standard error [SE] 0.99), −12.55% (0.96), −13.66% (0.97), and −16.09% (0.94), respectively, whereas the participants receiving 24 mg QW presented a change of −16.69% (0.98), and the placebo group presented a change of −1.15% (0.87). The treatment differences between bofanglutide and placebo were −8.60% (95% confidence interval [CI]: −11.18 to −6.01), −11.39% (−13.92 to −8.87), −12.50% (−15.07 to −9.94), and −14.94% (−17.45 to −12.42), respectively, across the Q2W dose range of 12–48 mg, and −15.54% (−18.10 to −12.98) for 24 mg QW (*p* < 0.001 for all comparisons). Sensitivity analyses via the analysis of covariance (ANCOVA) model with last observation carried forward (LOCF) and supporting analysis via a mixed model for repeated measures (MMRM) also confirmed the robust and dose-dependent weight-loss effect of bofanglutide at week 30 (supplementary Table 2). Compared with the placebo group, all bofanglutide treatment groups demonstrated significantly greater reductions in body weight at week 30 (−9.01 to −15.73 kg vs. −1.01 kg) (*p* < 0.001 for all comparisons) (Fig. 2c and Table 2).

**Table 2:** Primary and secondary endpoints

	Bofanglutide Q2W				Bofanglutide QW 24 mg (*N* = 53)	Placebo (*N* = 66)
	12 mg (*N* = 52)	18 mg (*N* = 53)	24 mg (*N* = 52)	48 mg (*N* = 64)		
**Primary endpoint** - Percentage change from baseline in body weight						
Percentage change, %	−9.75 (0.99)	−12.55 (0.96)	−13.66 (0.97)	−16.09 (0.94)	−16.69 (0.98)	−1.15 (0.87)
ETD versus placebo	−8.60 (−11.18, −6.01) *p* < 0.001	−11.39 (−13.92, −8.87) *p* < 0.001	−12.50 (−15.07, −9.94) *p* < 0.001	−14.94 (−17.45, −12.42) *p* < 0.001	−15.54 (−18.10, −12.98) *p* < 0.001	
**Secondary endpoints**						
Proportion of participants achieving weight loss targets						
≥5%, *n* (%)	38 (74.0)	46 (87.5)	46 (89.1)	61 (96.8)	49 (93.0)	12 (18.5)
ETD versus placebo	58.4 (43.5, 73.3) *p* < 0.001	71.7 (58.8, 84.6) *p* < 0.001	73.7 (62.1, 85.4) *p* < 0.001	81.6 (72.5, 90.7) *p* < 0.001	77.2 (65.6, 88.8) *p* < 0.001	
≥10%, n (%)	26 (51.9)	33 (62.2)	38 (74.7)	52 (82.7)	47 (88.7)	4 (6.7)
ETD versus placebo	46.0 (32.7, 59.2) *p* < 0.001	55.8 (42.5, 69.0) *p* < 0.001	68.9 (54.7, 83.2) *p* < 0.001	81.3 (70.4, 92.3) *p* < 0.001	82.1 (71.4, 92.9) *p* < 0.001	
≥15%, n (%)	13 (25.7)	19 (36.4)	21 (42.4)	39 (61.5)	34 (64.5)	2 (3.9)
ETD versus placebo	20.1 (7.5, 32.7) *p* < 0.001	32.5 (20.3, 44.7) *p* < 0.001	37.6 (22.5, 52.8) *p* < 0.001	59.2 (46.2, 72.2) *p* < 0.001	60.7 (47.2, 74.1) *p* < 0.001	
≥20%, n (%)	4 (9.0)	9 (16.7)	9 (19.4)	25 (39.2)	20 (37.3)	2 (3.5)
ETD versus placebo	4.7 (−5.6, 15.0) *p* = 0.260	13.6 (3.9, 23.3) *p* = 0.022	16.7 (4.7, 28.8) *p* = 0.008	37.3 (23.9, 50.6) *p* < 0.001	34.3 (20.1, 48.4) *p* < 0.001	
Change from baseline in body weight, kg	−9.01 (0.94)	−11.88 (0.91)	−12.89 (0.93)	−15.14 (0.89)	−15.73 (0.93)	−1.01 (0.83)
ETD versus placebo	−8.00 (−10.46, −5.55) *p* < 0.001	−10.87 (−13.28, −8.46) *p* < 0.001	−11.88 (−14.33, −9.43) *p* < 0.001	−14.13 (−16.51, −11.74) *p* < 0.001	−14.72 (−17.16, −12.29) *p* < 0.001	
Change from baseline in BMI, kg/m^2^	−3.39 (0.33)	−4.18 (0.32)	−4.56 (0.33)	−5.78 (0.32)	−5.68 (0.32)	−0.32 (0.29)
ETD versus placebo	−3.07 (−3.93, −2.20) *p* < 0.001	−3.86 (−4.71, −3.01) *p* < 0.001	−4.24 (−5.10, −3.38) *p* < 0.001	−5.46 (−6.32, −4.60) *p* < 0.001	−5.36 (−6.21, −4.52) *p* < 0.001	
Change from baseline in waist circumference, cm	−9.35 (0.92)	−10.74 (0.90)	−11.86 (0.96)	−14.24 (0.90)	−13.69 (0.91)	−2.23 (0.82)
ETD versus placebo	−7.11 (−9.55, −4.67) *p* < 0.001	−8.50 (−10.90, −6.10) *p* < 0.001	−9.62 (−12.10, −7.15) *p* < 0.001	−12.01 (−14.44, −9.58) *p* < 0.001	−11.45 (−13.87, −9.04) *p* < 0.001	

For continuous variables, the data are presented as the LSM (SE) for changes from baseline and the LSM (95% CI) for ETD from ANCOVA of the mITT population. For categorical variables, the data are *n* (%) and ETD (95% CI) from the logistic regression model. The proportions of participants who reached weight loss targets (≥ 5%, ≥ 10%, ≥ 15%, and ≥ 20%) were summarized via Rubin’s formula. Participants with missing values at week 30 were imputed via the multiple imputation method. Statistical significance for the comparison between the bofanglutide and placebo groups at the end of treatment was assessed via *p*-values. *ANCOVA* analysis of covariance, *ETD* estimated treatment difference, *mITT* modified intention-to-treat, *LSM* least squares mean, *SE* standard error, *CI* confidence interval, *QW* once weekly, *Q2W* biweekly

Subgroup analyses revealed that greater percentage reductions in body weight were attained by female participants than by male participants following treatment with bofanglutide (supplementary Table 3).

### Secondary and exploratory endpoints

At week 30, a significantly greater proportion of participants in the bofanglutide groups achieved body weight reductions of 5% or more, 10% or more, 15% or more, and 20% or more from baseline than those in the placebo group (*p* < 0.001 for all bofanglutide groups versus placebo) (Fig. 2d and Table 2). Specifically, a reduction in body weight of 5% or more, 10% or more, 15% or more, and 20% or more reached 96.8%, 82.7%, 61.5%, and 39.2%, respectively, in participants who received 48 mg of Q2W; 93.0%, 88.7%, 64.5% and 37.3%, respectively, in those who received 24 mg of QW; and 18.5%, 6.7%, 3.9% and 3.5%, respectively, in those who received placebo. No clear difference was observed in the proportions between the 48 mg Q2W and 24 mg QW groups. Similar to body weight, significant reductions in waist circumference (9.35 to 14.24 cm versus 2.23 cm) and BMI (3.39 to 5.78 kg/m^2^ versus 0.32 kg/m^2^) were observed for all bofanglutide doses compared with the placebo from baseline at week 30 (Table 2 and supplementary Fig. 2). Nevertheless, the magnitude of these reductions was comparable between the 48 mg Q2W and 24 mg QW groups.

Treatment with bofanglutide also led to favorable changes in a range of other cardiometabolic parameters. At week 30, compared with placebo, all bofanglutide doses groups (except the 12 mg Q2W group) demonstrated significant reductions in systolic and diastolic blood pressure, total cholesterol, low-density lipoprotein (LDL) cholesterol, and triglycerides, whereas improvements in high-density lipoprotein (HDL) cholesterol were less pronounced (supplementary Table 4 and supplementary Fig. 3). Furthermore, significant reductions in glycated hemoglobin (HbA1c), fasting plasma glucose (FPG), and fasting insulin levels were observed for all bofanglutide doses compared with placebo at week 30, accompanied by improvements in the homeostatic model assessment of insulin resistance (HOMA-IR) (supplementary Table 4). Additionally, treatment with bofanglutide was associated with significant reductions in aminotransferase (ALT), aspartate aminotransferase (AST), and serum uric acid levels across all bofanglutide doses groups at week 30 (supplementary Table 4 and supplementary Fig. 4).

A comparative analysis of the Impact of Weight on Quality of Life-Lite Clinical Trials Version (IWQoL-Lite-CT) total and physical function scores revealed significant improvements in all bofanglutide groups at week 30 compared with the placebo group (supplementary Table 5). The mean changes from baseline to week 30 ranged from 8.5 to 12.7 and from 7.5 to 11.4, respectively, across the bofanglutide groups versus 8.0 and 6.6, respectively, in the placebo group. Similar improvements were observed in the Short Form-36 Health Survey (SF-36) physical functioning norm-based score (PF_NBS) and physical component summary, with changes ranging from 1.86–3.17 and 1.93–3.77, respectively, compared with 1.01 and 1.67, respectively, in the placebo group (supplementary Table 5). In addition, there were no differences in the changes in the SF-36 mental component scores between the bofanglutide and placebo groups after 30 weeks of treatment, with ranges of 0.64–1.87 and 1.28, respectively.

### Safety endpoints

Treatment-emergent adverse events (TEAEs) of any grade were reported in 98.9% (271/274) of participants receiving bofanglutide and 86.4% (57/66) of participants receiving placebo, with most events being grade 1–2 in severity according to common terminology criteria for adverse events (CTCAE) criteria (Table 3). Serious adverse events (SAEs) were reported in eight participants (2.9%) in the bofanglutide group and one (1.5%) in the placebo group. Among these events, only one case of liver failure in the placebo group, was considered by the investigator to be related to the investigational product (IP). This event was possibly associated with the participant’s history of fatty liver and recovered after appropriate treatment. No deaths were observed. TEAEs leading to trial discontinuation occurred in 1.9% to 15.6% of participants receiving bofanglutide and 4.5% of those receiving placebo, with the highest rate in the bofanglutide 48 mg Q2W group, primarily due to gastrointestinal AEs during the dose-escalation period.

**Table 3:** Treatment-emergent adverse events

	Bofanglutide Q2W				Bofanglutide QW 24 mg (*N* = 53)	Placebo (*N* = 66)
	12 mg (*N* = 52)	18 mg (*N* = 53)	24 mg (*N* = 52)	48 mg (*N* = 64)		
Any TEAE	50 (96.2)	52 (98.1)	52 (100.0)	64 (100.0)	53 (100.0)	57 (86.4)
IP-related TEAE	46 (88.5)	50 (94.3)	47 (90.4)	62 (96.9)	46 (86.8)	40 (60.6)
IP-related Grade 3 or greater TEAE	0 (0.0)	2 (3.8)	2 (3.8)	2 (3.1)	1 (1.9)	1 (1.5)
SAE	2 (3.8)	3 (5.7)	1 (1.9)	0 (0.0)	2 (3.8)	1 (1.5)
Death	0 (0.0)	0 (0.0)	0 (0.0)	0 (0.0)	0 (0.0)	0 (0.0)
IP-related SAE	0 (0.0)	0 (0.0)	0 (0.0)	0 (0.0)	0 (0.0)	1 (1.5)
TEAE leading to trial discontinuation	4 (7.7)	1 (1.9)	4 (7.7)	10 (15.6)	5 (9.4)	3 (4.5)
AESI	41 (78.8)	42 (79.2)	45 (86.5)	58 (90.6)	44 (83.0)	23 (34.8)
Hypoglycemia	2 (3.8)	5 (9.4)	1 (1.9)	0 (0.0)	2 (3.8)	1 (1.5)
Severe hypoglycemia	0 (0.0)	0 (0.0)	0 (0.0)	0 (0.0)	0 (0.0)	0 (0.0)
Gastrointestinal AE	41 (78.8)	42 (79.2)	45 (86.5)	58 (90.6)	44 (83.0)	22 (33.3)
Nausea	18 (34.6)	25 (47.2)	25 (48.1)	41 (64.1)	20 (37.7)	6 (9.1)
Vomiting	13 (25.0)	15 (28.3)	24 (46.2)	40 (62.5)	28 (52.8)	8 (12.1)
Diarrhea	14 (26.9)	19 (35.8)	22 (42.3)	24 (37.5)	26 (49.1)	12 (18.2)
IP-related AESI	39 (75.0)	41 (77.4)	45 (86.5)	58 (90.6)	44 (83.0)	21 (31.8)
IP-related injection site reaction	18 (34.6)	34 (64.2)	25 (48.1)	28 (43.8)	27 (50.9)	22 (33.3)

Data are *n* (%), safety population. *TEAE* treatment-emergent adverse event, *IP* investigational product, *SAE* serious adverse event, *AESI* adverse events of special interest, *QW* once weekly, *Q2W* biweekly

Gastrointestinal AEs were defined as any event classified under the System Organ Class “gastrointestinal disorders” and the preferred term “decreased appetite”. Severe hypoglycemia is defined as no specific glucose threshold, but hypoglycemia is associated with severe cognitive impairment requiring external assistance for recovery

A total of 253 participants (74.4%) experienced 1695 adverse events of special interest (AESI), including gastrointestinal adverse events (AEs) and hypoglycemic episodes (Table 3). Gastrointestinal AEs were the most common TEAEs, including nausea, vomiting, and diarrhea, which occurred in 78.8% to 90.6% of participants in the bofanglutide groups and 33.3% in the placebo group. The majority of gastrointestinal AEs were grade 1–2 in severity, occurring primarily during the early stages of the dose-escalation period and diminishing toward the end of the trial (supplementary Figs. 5, 6). Hypoglycemic episodes were reported in eleven participants, of which eight (2.9%) in the bofanglutide groups and one (1.5%) in the placebo group were deemed IP-related by the investigator. Notably, all hypoglycemia was classified as grade 1, as defined by blood glucose levels less than 3.9 mmol/L and greater than 3.0 mmol/L. There were no reports of severe hypoglycemia.

Only four participants (two with 24 mg Q2W; one with 24 mg QW; one with placebo) had ALT elevations >3× the upper limit of normal (ULN) during the trial, three of whom (one with 24 mg Q2W; one with 24 mg QW; one with placebo) had concurrent AST elevations >3 × ULN. Most elevations were transient and returned to normal with continued treatment. Lipase elevations >3 × ULN were observed in only two participants (one with 12 mg Q2W and one with 18 mg Q2W) at week 12 and week 8, respectively. No amylase elevation >3×ULN or pancreatitis was reported in any participant.

No associations were detected between bofanglutide and mental health outcomes, as measured by the Patient Health Questionnaire (PHQ-9). Furthermore, no cases of suicidal ideation or behavior were reported according to assessments using the Columbia-Suicide Severity Rating Scale (C-SSRS). A total of 154 participants (45.3%) experienced IP-related injection site reactions (mostly injection site pain), including 132 participants (48.2%) who received bofanglutide and 22 (33.3%) who received placebo (Table 3). In addition, the presence of anti-bofanglutide antibodies during treatment was detected in 32.7% to 52.8% of the participants across the bofanglutide dose groups. No significant differences in body weight reduction or the incidence of TEAEs were observed between participants with and without anti-bofanglutide antibodies. No participants in the trial developed neutralizing antibodies.

### Pharmacokinetics endpoints

The steady-state systemic exposure of bofanglutide increased in a dose-dependent manner. Following the last dose, the mean maximum plasma concentration (C_max_) and area under the curve over the dosing interval (AUC_0–τ_) for the Q2W regimen ranged from 1236.9 ± 238.1 to 4445.8 ± 903.4 ng/mL and from 257,146.5 ± 59,187.8 to 964,657.9 ± 155,176.8 h*ng/mL, respectively, across the 12 to 48 mg dose range; the corresponding values for the 24 mg QW group were 4766.4 ± 2162.4 ng/mL and 645,662.1 ± 264,131.3 h*ng/mL, respectively (supplementary Table 6). The estimated β value (90% CI) of these two parameters was contained within the acceptance interval for the Q2W regimen (supplementary Table 7). In addition, the mean half-life (t_1/2_) values ranged from 156.2 ± 16.7 h to 176.7 ± 41.0 h across all bofanglutide treatments (supplementary Table 6).

## Discussion

In this randomized, double-blind, placebo-controlled phase 2b trial, biweekly (12/18/24/48 mg Q2W) or weekly (24 mg QW) bofanglutide achieved mean placebo-adjusted body weight reductions of up to 14.9% and 15.5%, respectively, after 30 weeks in Chinese adults with overweight or obesity. This finding confirms that bofanglutide administered Q2W has a similar efficacy to that of QW at an equipotent dose. The observed trajectory of body weight reduction suggested that a plateau had not been reached at the end of the treatment period. Furthermore, participants treated with bofanglutide showed improvements in multiple cardiovascular and metabolic risk factors. Like other GLP-1 RAs, bofanglutide was associated with mild to moderate gastrointestinal AEs, primarily during the dose-escalation period of the trial, but was otherwise generally well tolerated across all dose levels, and no IP-related SAEs were reported. The present study provides initial evidence for the efficacy and safety of bofanglutide at a Q2W dosing interval for the treatment of overweight and obesity, which will be confirmed in phase 3 trials.

Bofanglutide demonstrated a substantial dose-dependent body weight reduction after 30 weeks of treatment, particularly at doses of 48 mg Q2W and 24 mg QW, which induced a greater than 15% placebo-adjusted body weight reduction. Despite the relatively short treatment period, this outcome is comparable to or exceeds established GLP-1 RAs for body weight management. In the STEP7 study, where 80% of participants were Chinese, 2.4 mg semaglutide achieved a placebo-adjusted body weight reduction of 8.5% after 44 weeks of treatment.^24^ Similarly, in the SURMOUNT-CN study, 52 weeks of tirzepatide treatment resulted in placebo-adjusted body weight reductions of 11.3% and 15.1% at 10 mg and 15 mg, respectively, in Chinese participants with overweight or obesity.^25^ Most of the participants who received bofanglutide at doses of 48 mg Q2W and 24 mg QW achieved a weight reduction of 5% or more from baseline. In the 48 mg Q2W group, over 80% of the participants achieved 10% or more, more than two-thirds (≥67%) achieved 15% or more, and nearly 40% achieved 20% or more. This represents a high magnitude of efficacy relative to other GLP-1 RAs and approaches that achieved with bariatric surgeries.^26^ Additionally, given that participants continued to lose weight at the end of the trial, an extended intervention in the ongoing phase 3 trials (NCT06728124; NCT07150975) may achieve greater body weight reductions.

In addition to body weight reduction, bofanglutide at all doses significantly lowered HbA1c, FPG, and HOMA-IR, suggesting improved glycemic control and reduced insulin resistance even in nondiabetic individuals with overweight or obesity. Furthermore, bofanglutide improved other cardiometabolic risk factors, including waist circumference, blood pressure, and blood lipid profiles. Given the high prevalence of hepatic steatosis (92.9%), hyperlipidemia (45.9%) and hyperuricemia (38.8%) at baseline, the observed reductions in blood pressure, blood lipids, liver enzymes, and serum uric acid with bofanglutide treatment indicate comprehensive cardiometabolic benefits in Chinese adults with overweight or obesity. These improvements were consistent with phase 2a results in participants with obesity,^23^ indicating the substantial potential of bofanglutide to alleviate metabolic disorders.

Bofanglutide was generally well tolerated, with most TEAEs being mild to moderate in severity. The most common IP-related TEAEs were nausea, vomiting, and diarrhea, which is consistent with established GLP-1 RAs.^27,28^ These events occurred predominantly during the early dose-escalation period and gradually decreased over time, which aligns with the findings observed in the phase 2a trial and indicates the tolerance process.^23^ The higher incidence of gastrointestinal AEs may be attributed to fast escalation during a relatively short dose-escalation period. This effect was particularly pronounced in the bofanglutide 48 mg Q2W group, which utilized larger dose increments (i.e., no titration at 9 mg and 18 mg) than the other dose groups. As illustrated in supplementary Fig. 7a, incorporating 9 mg and 18 mg as intermediate doses substantially reduced the incidence of gastrointestinal AEs. Furthermore, per the prespecified protocol criteria, participants who experienced intolerable AEs at a titration dose of ≤12 mg were required to discontinue the trial and were not allowed to maintain a lower dose (≤12 mg). This criterion contributed to the highest number of discontinuations in the 48 mg Q2W group, most of which occurred during the direct escalation from 6 mg to 12 mg (supplementary Fig. 7b). These findings suggest that an improved dose-escalation scheme in phase 3 trials may help minimize the incidence of gastrointestinal AEs and trial discontinuations related to these AEs.^29^ Notably, although trial discontinuation rates attributed to TEAEs were higher in most bofanglutide groups (1.9% to 15.6%) than in the placebo group (4.5%), they were comparable to the published phase 2 trial results of other GLP-1 analogs for the treatment of overweight or obesity, with 4% to 17% for semaglutide and 6% to 16% for retatrutide.^27,28^ Injection site pain was reported in both the bofanglutide and placebo groups, representing 61.1% of all injection site reactions, followed by pruritus (approximately 15% of events). Since injection site reactions also occurred in the placebo group and no clear relationship was observed between bofanglutide dose and their incidence, it can be hypothesized that these reactions may be related to the solvent used in the phase 2b trial, which will be optimized in future studies. Approximately 32% of the participants developed anti-bofanglutide antibodies during treatment, but none of the participants developed neutralizing antibodies. No cases of suicidal ideation or behavior were reported, and no noticeable changes in mental health scores were observed; however, further investigations in larger populations and extended clinical trials are needed.

Chronic weight management requires consistent and long-term treatment.^30^ In such cases, GLP-1 RAs administered once weekly compared with once daily have been shown to promote enhanced adherence and persistence among patients, accompanied by better therapeutic outcomes.^31^ The observed similar efficacy of equipotent doses of Q2W and QW may be due to the comparable plasma exposure of bofanglutide between 48 mg Q2W and 24 mg QW groups, where the steady-state plasma exposures (C_max_ and AUC_0-168h_) were 4445 versus 4766 ng/mL and 599,492 versus 645,662 h*ng/mL, respectively. The population pharmacokinetic model further confirmed consistent exposure (AUC_0-336h_: 1,133,000 and 1,138,000 h*µg/L) between the two regimens at steady state (supplementary Fig. 8). Although a numerically higher incidence of gastrointestinal AEs and a higher discontinuation rate were observed in the 48 mg Q2W group, this is most likely attributable to its faster and relatively aggressive dose-escalation scheme and is less likely due to the higher doses administered under a biweekly regimen. Further evaluation of this dosing regimen is being conducted in several phase 3 confirmatory trials, which adopt an optimal and gentle dose-escalation scheme (24 mg [3–6–9–12–18–24 mg] or 48 mg [3–6–9–12–18–24–36–48 mg]). Additionally, the incidence of gastrointestinal AEs decreased during the second week of each biweekly dosing interval (Supplementary Figs. 5, 6). This trend suggests that a biweekly regimen may offer a period of reduced AE burden between doses. Taken together, both the pharmacokinetic and pharmacodynamic results support the feasibility of a biweekly dosing frequency without compromising efficacy and safety.

The strengths of this trial include the approximately equal sex representation and a design that featured, for the first time, an investigation into the dose-exposure relationship of a biweekly regimen alongside a direct comparison of weekly and biweekly dosing. This study also has several limitations. First, the relatively short treatment period of the current exploratory 2b trial may have influenced the estimation of bofanglutide’s maximal efficacy, as most participants likely require long-term pharmacotherapy for optimal weight reduction and other metabolic improvements. Second, all participants were of Chinese, which limits the extrapolation of the current findings to other races or ethnicities. To evaluate its applicability in a more diverse population, a phase 2 trial of bofanglutide is currently underway in the United States (NCT06737042). Third, the slightly higher dropout rate observed in participants in the high-dose group highlights the need to further optimize the dose-escalation strategy.

In conclusion, 30 weeks of biweekly bofanglutide treatment at doses ranging from 12 to 48 mg resulted in significant, dose-dependent body weight reductions, accompanied by apparent improvements across a range of cardiometabolic risk factors. The safety profiles of bofanglutide were manageable at doses up to 48 mg, despite a slight increase in the incidence of mild to moderate gastrointestinal AEs. Compared with the 24 mg QW dose, the 48 mg Q2W dose demonstrated comparable in vivo drug exposure, weight-loss effects, and safety profiles, suggesting the feasibility of biweekly dosing with bofanglutide. These findings collectively provide a strong rationale for the further development of bofanglutide as a biweekly formulation for effective weight management.

## Materials and methods

### Trial design and participants

This multicenter, randomized, double-blind, placebo-controlled phase 2b trial was conducted at 20 sites in China. Eligible adults were aged 18 to 75 years (both inclusive), were classified as overweight (body mass index [BMI] of 24 to less than 28 kg/m^2^) with at least one predefined weight-related comorbidity (one or more conditions of prediabetes, hypertension, dyslipidemia, and fatty liver; weight-bearing joint pain; obesity-related dyspnea or accompanied by obstructive sleep apnea syndrome), or were classified as obese (BMI of 28 kg/m^2^ or more) according to the Chinese BMI classification standard.^32^ Participants who met the specified criteria were required to demonstrate an understanding of the trial procedures and methods and express a willingness to comply with the trial protocol. The detailed inclusion and exclusion criteria are listed in the supplementary materials. All participants provided written informed consent.

### Randomization and masking

The randomization schedule for the study was generated by an independent statistician who was not involved in the clinical operation of the study. SAS software and a block randomization method were used to generate random numbers. Eligible participants were assigned in a 5:5:5:6:5 ratio to one of five dose groups: 12 mg biweekly (once every two weeks; Q2W), 18 mg Q2W, 24 mg Q2W, 48 mg Q2W and 24 mg once weekly (QW) (supplementary Fig. 1). Within each dose group, participants were further randomized at a 4:1 ratio to receive bofanglutide (*n* = 52) or placebo (*n* = 13), except for the 48 mg Q2W group, which used a 5:1 ratio (65 with bofanglutide and 13 with placebo) to ensure a sufficiently large population for safety assessment. Concealment was conducted via an interactive web response system (IWRS).

To minimize bias, a double-blind design was adopted for bofanglutide and placebo within each dose group. The bofanglutide and placebo formulations were indistinguishable with respect to labeling and physical appearance. Investigators and participants remained blinded to the administration of bofanglutide and placebo until after database lock.

### Procedures

The study design included a one-week screening period, a 30-week treatment period (including a dose-escalation period of 10–18 weeks and a dose-maintenance period of 12–20 weeks at target doses), and a three-week safety follow-up period.

During the 30-week treatment period, the investigational product (IP; bofanglutide or placebo) was administered by the study nurses or by the participants themselves via subcutaneous injections via reusable pen delivery devices (GanleePen^®^) at either Q2W or QW intervals. For each bofanglutide dose group, a matching placebo with the same injection volume and schedule was provided. Bofanglutide or placebo was administered biweekly at one of four dose-escalation schedules: 12 mg (3–6–9–12 mg), 18 mg (3–6–9–12–18 mg), 24 mg (3–6–9–12–18–24 mg), and 48 mg (3–6–12–24–36–48 mg) or once weekly at 24 mg (3–6–9–12–15–24 mg) for a total treatment duration of 30 weeks, with doses escalating every 2–4 weeks. The detailed dose-escalation schedules are illustrated in supplementary Fig. 1; the rationale for the dose-escalation setting and dose adjustment is provided in the supplementary materials.

For self-injections, the dosage was documented via participant diaries and preinjection photographs of the GanleePen^®^. All the injection cartridges used were returned at subsequent visits to verify the self-administered doses against these records. Medication adherence (%) was defined as the total administered dose divided by the total prescribed dose and then multiplied by 100.

Throughout the trial, participants received standardized lifestyle intervention under the guidance of site investigators, including dietary counseling targeting an energy deficit of approximately 500 kcal/day and a physical activity program comprising 150 min to 220 min of moderate-intensity aerobic and strength exercises per week, following Chinese guidelines for weight management.^33–35^

Study visits were scheduled at screening (Day -7 to Day -1), week 0 (Day 0; baseline), every two weeks from week 2 to week 30, and again at week 33 (safety follow-up). Participants who experienced intolerable adverse events at a titration dose of ≤12 mg were required to discontinue the trial after investigator evaluation and were not permitted to maintain a lower dose (≤12 mg). All efforts were made to conduct the week 30 visit for all participants, including those who discontinued treatment prematurely.

### Endpoints

The primary endpoint was the percentage change in body weight from baseline to week 30. The secondary efficacy endpoints included the proportion of participants who achieved body weight loss of 5% or more, 10% or more, 15% or more, and 20% or more at week 30. Additional secondary endpoints included assessments of changes from baseline to week 30 in body weight, waist circumference, and BMI, as well as alterations in glucose metabolism parameters (glycated hemoglobin [HbA1c], fasting plasma glucose [FPG], fasting insulin, and homeostatic model assessment for insulin resistance [HOMA-IR]). Changes from baseline to week 30 in other cardiovascular risk factors (blood pressure and lipid profiles, including total cholesterol, low-density lipoprotein [LDL] cholesterol, high-density lipoprotein [HDL] cholesterol, and triglycerides) were also evaluated. Exploratory endpoints included changes in serum uric acid, aminotransferase (ALT), and aspartate aminotransferase (AST) levels from baseline to week 30. Furthermore, changes in the Impact of Weight on Quality of Life-Lite Clinical Trials Version (IWQOL-Lite-CT) and the Short Form-36 Health Survey (SF-36) questionnaires were included. The pharmacokinetic endpoints included the area under the concentration‒time curve.

Safety endpoints included the incidence of treatment-emergent adverse events (TEAEs), serious adverse events (SAEs), and adverse events of special interest (AESI), specifically hypoglycemic episodes and gastrointestinal AEs (e.g., nausea, vomiting, diarrhea, and constipation). The severity of TEAEs was assessed via the Common Terminology Criteria for Adverse Events (CTCAE; version 5.0). Potential associations with treatment were assessed by the investigators on the basis of prespecified criteria. Hypoglycemic episodes were defined as participant-reported symptoms and/or a confirmed plasma glucose level of ≤3.9 mmol/L. Assessments of mental health were facilitated by the Patient Health Questionnaire (PHQ-9) and the Columbia-Suicide Severity Rating Scale (C-SSRS). The immunogenicity endpoints included anti-bofanglutide antibodies and neutralizing antibodies.

### Statistical analysis

The sample size calculation assumed an 11% difference in the mean percentage reduction in body weight from baseline to week 30 for the optimal bofanglutide dose compared with the placebo, on the basis of the study results of similar drugs and the nonclinical/clinical data of bofanglutide. Assuming a standard deviation of 10% and a dropout rate of 20%, a sample size of 338 participants (65 in each dose group except 78 in the 48 mg Q2W dose group) was calculated to provide at least 80% power to detect a clinical difference (one-sided *p* = 0.025). Specifically, given the potential for some participants in the bofanglutide 48 mg Q2W group to experience gastrointestinal intolerance and thus be unable to escalate to 48 mg, the study population in the 48 mg Q2W dose group was expanded to 78 participants (65 receiving bofanglutide and 13 receiving placebo).

The primary efficacy endpoints were analyzed via the modified intention-to-treat (mITT) population, which consisted of all randomized participants who received at least one dose of the IP. The treatment-regimen estimator was used to assess the treatment effect of bofanglutide relative to placebo at 30 weeks, regardless of adherence to treatment (according to the treatment policy strategy in the ICH E9 [R1] addendum).^36^ Safety endpoints were analyzed via the safety population, which consisted of all randomized participants who received at least one dose of the IP and had at least one safety evaluation.

The primary analysis for the primary endpoint was performed in the mITT population via an analysis of covariance (ANCOVA) model, with group as a fixed effect and the baseline body weight value as a covariate. Missing data at week 30 were imputed via the multiple imputation method. For each bofanglutide group, imputations were derived from the observed data of the pooled placebo group, whereas that for the placebo group was performed using its own observed data. The least squares mean (LSM) of the percentage change from baseline for each group and its two-sided 95% confidence interval (CI) were calculated. Each bofanglutide group was compared with the pooled placebo group, with the estimated LSM difference (bofanglutide group-placebo group), its two-sided 95% CI, and the corresponding *p* values presented for each comparison. A sensitivity analysis of the primary endpoint was conducted via the ANCOVA model described above, with the modification that missing data at week 30 were imputed via the last observation carried forward (LOCF) method. A supporting analysis of the primary endpoint was also conducted via a mixed model for repeated measures (MMRM) in the mITT population or per-protocol set (PPS) population (a subset of the mITT population, defined as participants who met all inclusion criteria and no exclusion criteria, had evaluable primary efficacy endpoints and avoided major protocol deviations), with treatment, baseline BMI (overweight or obesity), visit, and treatment-by-visit interaction as fixed effects, and baseline body weight value as a covariate. A subgroup analysis was conducted regarding BMI and sex.

The analysis of continuous variables for secondary efficacy endpoints was conducted via the same methodology as that employed for the primary endpoint analysis, with corresponding baseline values as covariates. For the categorical endpoints (i.e., proportions of participants who achieved prespecified weight loss targets), a logistic regression model was used, incorporating the same fixed effect and covariate as the model for the primary endpoint. Missing data were imputed via the multiple imputation method. Statistical inference over imputed data was guided by Rubin’s formula.

The safety data were summarized descriptively. All the statistical analyses were conducted via SAS version 9.4.

## Supplementary information

Supplementary materials

Clinical trial protocol

## Data Availability

The study protocol, including statistical analysis plan, and other supporting materials are available within this article and its supplementary files. The datasets generated and/or analyzed during the current study are not publicly available due to ethical and regulatory restrictions but can be requested from the corresponding author for non-commercial, academic purposes. All data-sharing requests will be reviewed by the corresponding author and the sponsor, Gan & Lee Pharmaceuticals, within 2 weeks of submission. Approved requests will require a signed data access agreement with the sponsor prior to the release of any data.

## References

[1] Blüher, M. Obesity: global epidemiology and pathogenesis. *Nat. Rev. Endocrinol.* **15**, 288–298 (2019). 30814686 10.1038/s41574-019-0176-8

[2] Bray, G. A. Obesity: a 100 year perspective. *Int J. Obes. (Lond.)* **49**, 159–167 (2025). 38714830 10.1038/s41366-024-01530-6

[3] WHO. *Obesity and overweight*. https://www.who.int/en/news-room/fact-sheets/detail/obesity-and-overweight (2025).

[4] Chen, K. et al. Prevalence of obesity and associated complications in China: A cross-sectional, real-world study in 15.8 million adults. *Diab. Obes. Metab.* **25**, 3390–3399 (2023). 10.1111/dom.15238 37589256

[5] Hemmingsson, E. The unparalleled rise of obesity in China: a call to action. *Int J. Obes. (Lond.)* **45**, 921–922 (2021). 33608648 10.1038/s41366-021-00774-w

[6] Fruh, S. M. Obesity: Risk factors, complications, and strategies for sustainable long-term weight management. *J. Am. Assoc. Nurse Pr.* **29**, S3–S14 (2017). 10.1002/2327-6924.12510 PMC6088226 29024553

[7] Valenzuela, P. L. et al. Obesity and the risk of cardiometabolic diseases. *Nat. Rev. Cardiol.* **20**, 475–494 (2023). 36927772 10.1038/s41569-023-00847-5

[8] Yamada, T., Kimura-Koyanagi, M., Sakaguchi, K., Ogawa, W. & Tamori, Y. Obesity and risk for its comorbidities diabetes, hypertension, and dyslipidemia in Japanese individuals aged 65 years. *Sci. Rep.* **13**, 2346 (2023). 36759688 10.1038/s41598-023-29276-7 PMC9911391

[9] Wolfenden, L., Ezzati, M., Larijani, B. & Dietz, W. The challenge for global health systems in preventing and managing obesity. *Obes. Rev.* **20**, 185–193 (2019). 31317659 10.1111/obr.12872

[10] Ruban, A., Stoenchev, K., Ashrafian, H. & Teare, J. Current treatments for obesity. *Clin. Med (Lond.)* **19**, 205–212 (2019). 31092512 10.7861/clinmedicine.19-3-205 PMC6542229

[11] Elmaleh-Sachs, A. et al. Obesity management in adults: a review. *JAMA* **330**, 2000–2015 (2023). 38015216 10.1001/jama.2023.19897 PMC11325826

[12] Müller, T. D., Blüher, M., Tschöp, M. H. & DiMarchi, R. D. Anti-obesity drug discovery: advances and challenges. *Nat. Rev. Drug Discov.* **21**, 201–223 (2022). 34815532 10.1038/s41573-021-00337-8 PMC8609996

[13] Chinese Society of Endocrinology. Guideline for chronic weight management and clinical practice of anti-obesity medications (2024 version) (in Chinese). *Chin. J. Endocrinol. Metab.* **40**, 545–564 (2024).

[14] Abbasi, J. Semaglutide updates, hypertension triple pills, and more-heart health highlights from ESC congress. *JAMA* **332**, 1319–1321 (2024). 10.1001/jama.2024.19544 39365594

[15] Jalleh, R. J. et al. Gastrointestinal effects of GLP-1 receptor agonists: mechanisms, management, and future directions. *Lancet Gastroenterol. Hepatol.* **9**, 957–964 (2024). 39096914 10.1016/S2468-1253(24)00188-2

[16] Ryan, D. H. et al. Long-term weight loss effects of semaglutide in obesity without diabetes in the SELECT trial. *Nat. Med.* **30**, 2049–2057 (2024). 10.1038/s41591-024-02996-7 PMC11271387 38740993

[17] Gasoyan, H. et al. Changes in weight and glycemic control following obesity treatment with semaglutide or tirzepatide by discontinuation status. *Obes. (Silver Spring)* **33**, 1657–1667 (2025). 10.1002/oby.24331 PMC12381620 40491239

[18] Sikirica, M. V. et al. Reasons for discontinuation of GLP1 receptor agonists: data from a real-world cross-sectional survey of physicians and their patients with type 2 diabetes. *Diab. Metab. Syndr. Obes.* **10**, 403–412 (2017). 10.2147/DMSO.S141235 PMC5630073 29033597

[19] Polonsky, W. H., Arora, R., Faurby, M., Fernandes, J. & Liebl, A. Higher Rates of Persistence and Adherence in Patients with Type 2 Diabetes Initiating Once-Weekly vs Daily Injectable Glucagon-Like Peptide-1 Receptor Agonists in US Clinical Practice (STAY Study). *Diab. Ther.* **13**, 175–187 (2022). 10.1007/s13300-021-01189-6 PMC8776963 34918213

[20] Zhang, M. et al. GZR18, a novel long-acting GLP-1 analog, demonstrated positive in vitro and in vivo pharmacokinetic and pharmacodynamic characteristics in animal models. *Eur. J. Pharm.* **928**, 175107 (2022). 10.1016/j.ejphar.2022.175107 35718129

[21] Liu, Y. et al. The safety, tolerability, pharmacokinetics and pharmacodynamics of GZR18 in healthy American and Chinese adult subjects. *Diab. Obes. Metab.* **27**, 2777–2789 (2025). 10.1111/dom.16285 40028667

[22] Knudsen, L. B., Hastrup, S., Underwood, C. R., Wulff, B. S. & Fleckner, J. Functional importance of GLP-1 receptor species and expression levels in cell lines. *Regul. Pept.* **175**, 21–29 (2012). 22252224 10.1016/j.regpep.2011.12.006

[23] Ji, L. et al. 1858-LB: A Novel GLP-1 Analog, GZR18, Induced an 18.6% Weight Reduction in Subjects with Obesity in a Phase Ib/IIa Trial. *Diabetes* **73**, 1858–18 (2024).

[24] Mu, Y. et al. Efficacy and safety of once weekly semaglutide 2·4 mg for weight management in a predominantly east Asian population with overweight or obesity (STEP 7): a double-blind, multicentre, randomised controlled trial. *Lancet Diab. Endocrinol.* **12**, 184–195 (2024). 10.1016/S2213-8587(23)00388-1 38330988

[25] Zhao, L. et al. Tirzepatide for weight reduction in Chinese adults with obesity: The SURMOUNT-CN randomized clinical trial. *JAMA* **332**, 551–560 (2024). 38819983 10.1001/jama.2024.9217 PMC11337071

[26] van Rijswijk, A. S., van Olst, N., Schats, W., van der Peet, D. L. & van de Laar, A. W. What Is Weight Loss After Bariatric Surgery Expressed in Percentage Total Weight Loss (%TWL)? A Systematic Review. *Obes. Surg.* **31**, 3833–3847 (2021). 34002289 10.1007/s11695-021-05394-x

[27] O’Neil, P. M. et al. Efficacy and safety of semaglutide compared with liraglutide and placebo for weight loss in patients with obesity: a randomised, double-blind, placebo and active controlled, dose-ranging, phase 2 trial. *Lancet* **392**, 637–649 (2018). 30122305 10.1016/S0140-6736(18)31773-2

[28] Jastreboff, A. M. et al. Triple-Hormone-Receptor Agonist Retatrutide for Obesity - A Phase 2 Trial. *N. Engl. J. Med* **389**, 514–526 (2023). 37366315 10.1056/NEJMoa2301972

[29] Nauck, M. A. et al. A Phase 2, Randomized, Dose-Finding Study of the Novel Once-Weekly Human GLP-1 Analog, Semaglutide, Compared With Placebo and Open-Label Liraglutide in Patients With Type 2 Diabetes. *Diab. Care* **39**, 231–241 (2016). 10.2337/dc15-0165 26358288

[30] Kheniser, K., Saxon, D. R. & Kashyap, S. R. Long-Term Weight Loss Strategies for Obesity. *J. Clin. Endocrinol. Metab.* **106**, 1854–1866 (2021). 33595666 10.1210/clinem/dgab091 PMC8427732

[31] Uzoigwe, C., Liang, Y., Whitmire, S. & Paprocki, Y. Semaglutide Once-Weekly Persistence and Adherence Versus Other GLP-1 RAs in Patients with Type 2 Diabetes in a US Real-World Setting. *Diab. Ther.* **12**, 1475–1489 (2021). 10.1007/s13300-021-01053-7 PMC8099966 33837922

[32] Pan, X. F., Wang, L. & Pan, A. Epidemiology and determinants of obesity in China. *Lancet Diab. Endocrinol.* **9**, 373–392 (2021). 10.1016/S2213-8587(21)00045-0 34022156

[33] Writing committee of expert consensus on overweight/obesity medical nutrition therapy in China. Expert consensus on overweight/obesity medical nutrition therapy in China (in Chinese). *Chin. J. Diab. Mellitus* **8**, 525–540 (2016).

[34] Chinese Diabetes Society. Guideline for the Prevention and Treatment of Diabetes Mellitus in China (2024 Edition) (in Chinese). *Chin. J. Diab. Mellit.* **17**, 16–139 (2021).

[35] Nutrition and Metabolism Management Branch of China International Exchange and Promotive Association for Medical and Health Care; Clinical Nutrition Branch of Chinese Nutrition Society; Chinese Diabetes Society; Chinese Society for Parenteral and Enteral Nutrition; Nutrition Physician Committee of Chinese Medical Doctor Association. Guidelines for medical nutrition treatment of overweight/obesity in China (in Chinese). *Chin. J. Front. Med. Sci.* (Electron Ed) **13**, 1–55 (2021).

[36] European Medicines Agency. ICH E9 (R1) addendum on estimands and sensitivity analysis in clinical trials to the guideline on statistical principles for clinical trials. https://www.ema.europa.eu/en/documents/scientific-guideline/ich-e9-r1-addendum-estimands-and-sensitivity-analysis-clinical-trials-guideline-statistical-principles-clinical-trials-step-5_en.pdf (2020).

