# Supplementary material for: Efficacy and safety of bofanglutide, a GLP-1 receptor agonist, in Chinese adults with overweight or obesity: a randomized, double-blind, placebo-controlled phase 2b trial
Source: Signal Transduct Target Ther. 2026 Feb 27;11:73. doi: 10.1038/s41392-026-02586-8 (PMC12948953; doi:10.1038/s41392-026-02586-8)
Supplement: Supplementary file 1 — Supplementary materials [file 41392_2026_2586_MOESM1_ESM.docx]

Supplementary Materials for

**Efficacy and safety of bofanglutide, a GLP-1 receptor agonist, in Chinese adults with overweight or obesity: a randomized, double-blind, placebo-controlled phase 2b trial**

Linong Ji^1,#,*^, Leili Gao^1,#^, Junhang Tian^2^, Ruihua Dong^3^, Zhongtao Zhang^3^, Hongyan Shu^4^, Jing Zhao^5^, Liyuan Zhao^5^, Anshun He^5^, Tian Xie^5^, Yue Li^5^, Wei Chen^5,6,*^

Correspondence to: jiln@bjmu.edu.cn; weichen45@126.com

**This PDF file includes:**

Materials and Methods

Supplementary Text

Figures. S1 to S8

Tables S1 to S7

Materials and Methods

Inclusion criteria

Participants must meet all of the following inclusion criteria to be eligible for the study:

1. Male or female aged 18–75 years (inclusive).

2. Participants who were obese (body mass index [BMI] ≥28 kg/m^2^), or overweight (24 kg/m^2^≤BMI<28 kg/m^2^) with at least one of the following: Concomitant with one or more of prediabetes, hypertension, dyslipidemia, fatty liver; weight-bearing joint pain; Obesity-induced dyspnoea or obstructive sleep apnoea syndrome.

*Note:* Prediabetes included impaired fasting glucose (IFG) and/or impaired glucose tolerance (IGT), based on World Health Organization 1999. Such as 7.8 mmol/L≤ oral glucose tolerance test (OGTT) 2 h post glucose<11.1 mmol/L and fasting plasma glucose (FPG) <7 mmol/L is IGT; such as 6.1 mmol/L≤FPG<7.0 mmol/L and OGTT 2 h plasma glucose <7.8 mmol/L is IFG.

| Classification of glucose metabolism | Venous plasma glucose (mmol/L) | |
| --- | --- | --- |
|  | FPG | OGTT 2h post glucose |
| Normal glucose regulation | <6.1 | <7.8 |
| Impaired fasting glucose | ≥6.1, <7.0 | <7.8 |
| Impaired glucose tolerance | <7.0 | ≥7.8, <11.1 |
| Diabetes | ≥7.0 | ≥11.1 |

The lower limit of normal reference range of FPG is usually 3.9 mmol/L. OGTT= Oral glucose tolerance test; FPG= Fasting plasma glucose.

3. Able to understand the procedures and methods in this study; willing and able to maintain a stable diet and exercise lifestyle during the research period, and willing to sign the Informed Consent Form (ICF) voluntarily.

Exclusion criteria

Participants will not be enrolled in the study if they meet any of the following criteria:

1. Limb deformities or defects affecting height and body weight measurement.

2. Pregnant or lactating women, men or women of reproductive potential unwilling to use contraception throughout the study and for 6 months after the last dose of investigational product.

3. History of drug abuse within 1 year before screening, or positive results in drug abuse screening during screening or before randomization.

4. Alcohol abuse history, defined as an average weekly alcohol consumption of more than 14 units for men/7 units for women (1 standard unit equivalent to 360 mL of beer or 150 mL of wine or 45 mL of spirits with 40% alcohol) within 6 months prior to screening.

5. Participants who are known or suspected to be allergic to glucagon-like peptide-1 (GLP-1) drugs or excipients.

6. Participants with an absolute weight change greater than 5.0% due to any reason within the 3 months prior to screening. The formula for calculating weight change is: (highest weight in the 12 weeks prior to screening - lowest weight)/highest weight * 100%.

7. History or evidence of any of the following:

1) Previous diagnosis of type 1 or type 2 diabetes mellitus;

2) Proliferative retinopathy within 1 year prior to screening or at screening;

3) Previous severe hypoglycemia or recurrent symptomatic hypoglycemia (≥2 episodes in half a year);

4) Secondary diseases or drugs leading to obesity, including: elevated cortisol (e.g. Cushing’s syndrome), pituitary and hypothalamic damage leading to obesity, weight loss medicine reduction/discontinuation leading to obesity, etc.;

5) Previous history of moderate to severe depression; or previous history of severe psychiatric disorders, such as schizophrenia, bipolar disorder, etc. Or when screening, the score of Patient Health Questionnaire (PHQ-9) ≥15;

6) Previous suicidal tendency or suicidal behavior, or when screening, the participants had suicidal ideation in categories 4 and 5 of the Columbia-Suicide Severity Rating Scale (C-SSRS);

7) Systolic blood pressure ≥160 mmHg and/or diastolic blood pressure ≥100 mmHg at screening;

8) Previous history of thyroid C-cell carcinoma, multiple endocrine neoplasia (MEN) type 2A or 2B or related family history, or previous history of malignancy within the past 5 years (except for cured basal cell carcinoma of the skin, squamous cell carcinoma of the skin, other polyps and carcinoma in situ of the cervix);

9) History of the following cardiovascular diseases: decompensated cardiac insufficiency (New York Heart Association [NYHA] Class III or IV), unstable angina or myocardial infarction, heart valve replacement surgery, coronary artery bypass grafting (CABG) or other invasive cardiovascular surgery including percutaneous coronary intervention, cerebrovascular accidents or stroke with sequelae;

10) History of acute and chronic pancreatitis, symptomatic gallbladder disease (excluding cholecystectomy), pancreatic injury, and other high-risk factors that may lead to pancreatitis;

11) Participants with previous gastrointestinal diseases such as gastroparesis, esophageal motility disorders, gastroesophageal reflux, chronic diarrhea, fecal incontinence, and constipation, who are deemed unsuitable for participation in this study by the investigators.

8. Satisfaction of any laboratory test parameter with the following criteria at screening:

1) Serum calcitonin ≥50 ng/L (pg/mL);

2) Alanine aminotransferase (ALT) ≥3.0 * upper limit of normal (ULN) and/or aspartate aminotransferase (AST) ≥3.0 * ULN and/or total bilirubin ≥2.0 * ULN;

3) Estimated glomerular filtration rate (eGFR) <60 mL/min/1.73 m^2^, estimated by Chronic Kidney Disease Epidemiology Collaboration (CKD-EPI) equation;

4) Presence of thyroid dysfunction (thyroid-stimulating hormone [TSH]) >6 mIU/L or <0.4 mIU/L);

5) Fasting triglycerides ≥5.64 mmol/L (500 mg/dL);

6) Blood amylase or lipase >1.5×ULN;

7) International normalized ratio (INR) of prothrombin time > upper limit of normal range;

8) Glycated hemoglobin <110 g/L (men) or <100 g/L (women).

9) Fasting venous glucose ≥7.0 mmol/L or venous blood glucose after a 2-hour OGTT (75 g) ≥ 11.1 mmol/L;

10) Participants with positive HIV antibody, treponema pallidum antibody, hepatitis B virus surface antigen (HBsAg) and hepatitis C virus (HCV) antibody.

9. Presence of the following clinically significant 12-lead electrocardiogram (ECG) abnormalities at screening: heart rate<50 beats/min or >100 beats/min, second- or third-degree atrioventricular (AV) block, long QT syndrome or QTc >500 ms (The calculation formula can be found in Attachment 4), left or right bundle branch block, pre-excitation syndrome, or other significant arrhythmia (other than sinus arrhythmia).

10. Blood donation and/or blood loss ≥400 mL or bone marrow donation within 3 months prior to screening, or hematological disorders (including but not limited to haemoglobinopathy, haemolytic anaemia, thalassaemia, sickle cell anaemia).

11. Participants who had undergone weight loss surgery except acupuncture and moxibustion, liposuction and abdominal liposuction within one year before screening;

12. Previous history of organ transplantation；or medium to major surgery, severe trauma and severe infection within 6 months prior to screening, unsuitable for participation in this study at the discretion of the investigator; or surgery scheduled during the study, except outpatient surgery posing no impact on the safety of participants and the study results at the discretion of the investigator.

13. Use of any of the following drugs or treatments within 3 months prior to screening:

1) GLP-1 receptor (GLP-1R) agonists or GLP-1R/ glucagon receptor (GCGR) agonists or glucose-dependent insulinotropic polypeptide receptor (GIPR)/GLP-1R agonists or GIPR/GLP-1R/GCGR agonists.

2) Drugs affecting body weight, including systemic steroids (intravenous, oral, or intra-articular administration), Antidepression such as selective serotonin reuptake inhibitors (SSRIs), serotonin noradrenaline reuptake inhibitors (SNRIs), tricyclics, tetracyclics, etc., other psychiatric agents or sedatives (e.g. imipramine, amitriptyline, mirtazapine, paroxetine, phenelzine, chlorpromazine, thioridazine, clozapine, olanzapine, valproic acid, valproic acid derivatives, lithium salts), diuretic, etc.;

3) Traditional Chinese medicine, Chinese patent drug, herbal medicine, health-care products, meal replacements, etc. that affect body weight.

4) Weight loss drugs, such as sibutramine hydrochloride, orlistat, phentermine, phenylpropanolamine, mazindol, phentermine, diethylpropion, lorcaserin, phentermine/topiramate, naltrexone/bupropion, etc.;

5) Hypoglycemic agents, such as metformin, α-glucosidase inhibitors, sulfonylureas, dipeptidyl peptidase-4 (DPP-4) inhibitors, sodium-glucose cotransporter-2 (SGLT-2) inhibitors, thiazolidinediones (TZDs), etc.;

14. Participation in clinical trials of other drugs, vaccines or medical devices within 3 months prior to screening and have received treatment.

15. Any other factor that, in the investigator’s opinion, may compromise the evaluation of efficacy or safety or otherwise render the participant unsuitable for the study.

Assessments for several endpoints

Body weight, waist circumference, vital signs, and adverse events were monitored at every visit. Blood chemistry (including blood lipids), serum calcitonin, lipase, amylase, and thyroid function test were tested at screening, weeks 8, 12, 16, 20, 24, 30, and 33. HbA1c, FPG, and fasting insulin were tested at screening, and week 30. Immunogenicity was monitored at weeks 0, 16, 30, and 33. Impact of Weight on Quality of Life-Lite-Clinical Trials (IWQOL-Lite-CT) and the 36-Item Short Form Health Survey (SF-36) (version 2) questionnaire assessments were conducted at weeks 0, 12, 24, and 30. PHQ-9 and C-SSRS assessment were conducted at screening, weeks 12, 24, and 33.

Supplementary Text

Summary of the study design

This trial protocol is based on its own non-clinical and clinical data, as well as the study designs of similar drugs, and demonstrates high feasibility and safety. The specific summary is as follows.

The initial titration dose setting

In the phase 1a trial, participants were given bofanglutide at a dose of 50 μg/kg, which was still well tolerated and safe, and the actual dose of bofanglutide administered in this dose group ranged from 2.84 to 4.06 mg, or approximately 3 to 4 mg. Although there was a single pre-adaptive administration of 10 μg/kg prior to the target dose of 50 μg/kg, the design of this dose group could not be used as the basis for the tolerability of a single dose, however, considering that the dose of 10 μg/kg is relatively small and the target dose of 50 μg/kg increases significantly (5 times) compared to it, it is believed that the effect of pre-adapted administration of 10 μg/kg can be ignored. 50 μg/kg can be roughly considered as a single well tolerated dose. Therefore, this study used 3 mg as the starting titration dose.

The highest dose setting

The conversion of non-clinical toxicological data for bofanglutide resulted in a single-dose human equivalent dose (HED) of approximately 81 mg~22.8 mg, and a weekly-dose HED of approximately 19.44 mg~68.04 mg. The completed clinical trial of bofanglutide showed that the participants tolerated well at a maximum dose of 30 mg.

Existing Phase 1a clinical data indicate that within the dose range of 20 to 50 μg/kg, bofanglutide exhibits a dose-proportional-response relationship, indicating that bofanglutide dose and exposure can be predicted through a linear relationship, which is also one of the safety foundations for further dose escalation.

The relationship between dose and adverse event (AE) in Phase 1b/2a Part B showed that AE is mainly concentrated in the early stage of dose escalation (3 to 5 mg). When the dose exceeded 7 mg, the number of AE no longer increased with dose escalation, but showed a significant decreasing trend. This suggests that bofanglutide has gradually developed tolerance in the human body after multiple doses, and it is expected that subsequent 24 mg once-weekly (QW) and 48 mg bi-weekly (Q2W) doses may not produce stronger gastrointestinal reactions and other AEs.

Finally, the similar drug semaglutide is still being re-explored for efficacy at higher doses (7.2 mg and 16 mg) after the 2.4 mg strength for an existing weight-loss indication has been marketed, suggesting that there is a trend in the development of increased doses of GLP-1 analogs to explore superior effects.

In summary, this study is planned to add a 48 mg Q2W dose group, and if the participants do not tolerate it, it is allowed to reduce it to 36 mg Q2W and continue to be administered. In order to explore the optimal choice of efficacy/safety/compliance for different dosing frequencies in comparison with the 48 mg Q2W group, and to provide more and more sufficient experimental basis for the dose selection of the subsequent clinical development, the target dose of the once-weekly group is set at 24 mg.

The dose escalation interval setting

The dose escalation regimen for the 48 mg Q2W dose group in the study is shown in the table below. The amplification factor between doses is maintained at 1.3 to 2 times. The rate of dose escalation in the high-dose group slows down, and it is expected to have certain feasibility.

| Number | Dose | Multiples of previous dose |
| --- | --- | --- |
| 1 | 3 mg | NA |
| 2 | 6 mg | 2 |
| 3 | 12 mg | 2 |
| 4 | 24 mg | 2 |
| 5 | 36 mg | 1.5 |
| 6 | 48 mg | 1.3 |

Safety monitoring

This study conducted a comprehensive safety monitoring on the participants average once a month, including laboratory tests, ECG tests, physical examinations, etc., to ensure that any potential AEs can be detected in a timely manner during the dose escalation process. And this study has also established strict withdrawal criteria and dose adjustment strategies. Participants can be discontinued from the trial based on safety assessment findings or at their own request. The above measures designed to maximize safety and protect the rights of the participants throughout the study.

Discontinuation of participants from treatment or study

Criteria for discontinuation

1. Withdrawal at the participants’ request.

2. Discontinuation as required by regulatory authorities.

3. Discontinuation of participation by the investigators: the investigator must discontinue a participation in the study if, in the investigator's judgment, it is in the participant's best interest to do so (e.g., due to unsuitability for further treatment with the investigational product).

1) Participants with any intolerable AEs at the titration dose of ≤12 mg should discontinue from the study after evaluation by the investigator.

2) In case participants have experienced AEs or serious adverse events (SAEs), the investigator considers that it is not recommended for them to further participate in the study;

3) The patient has a confirmed diagnosis of moderate to major depressive disorder or other severe psychiatric disorder (generalized anxiety, panic attack, bipolar disorder, schizophrenia, etc.), or a PHQ-9 score ≥15 in the study.

4) The patient with suicidal tendency or behavior, or with Type 4 and 5 suicidal ideas as measured by the C-SSRS in the study.

5) Participants who are pregnant, have any pregnancy plan, or unwilling to take effective contraception.

6) The patient is treated with the GLP-1 receptor agonist (RA) or DPP-4 inhibitor.

7) Participants with acute and chronic pancreatitis, symptomatic gallbladder disease (such as multiple gallstones in the gallbladder), pancreatic injury and other high-risk factors that may lead to pancreatitis.

8) Participants with suspected or confirmed thyroid C-cell carcinoma, MEN2A or 2B or other malignancies.

9) Participants with new or progressive abnormalities in thyroid-stimulating hormone (TSH >10 mIU/L) or calcitonin levels (calcitonin >50 pg/mL), as determined by the investigator to be unsuitable for further treatment with the investigational product.

10) Participants with severe hypoglycemic events (defined as hypoglycemic events requiring assistance from others to obtain carbohydrates, glucagon, or other rescue measures) or recurrent unexplained hypoglycemic events (at least two occurrences within 1 month);

11) Participants with poor compliance, affecting safety or tolerability judgment; including but not limited to:

- Participants failed to take the investigational product and receive examinations as required;
- Participants take other drugs or foods affecting safety evaluation.
- Participants cannot tolerate the dose level or escalation method assigned to him/her.
- Participants have other behaviors affecting the study results.

The investigator should attempt to obtain participant information if a participant discontinues from the study. For participants lost to follow-up, the investigator should make every effort (at least 3 telephone contacts with the participant) to complete the final evaluation and record it for documenting purpose (the date of telephone contact and the information summary should be included in the source documents). All evaluations and observations, along with a narrative describing the reason for participant exclusion, must be documented in the source documents. The electronic Case Report form (eCRF) must document the primary reason for withdrawal.

Dose adjustment strategies

The most common adverse reaction of GLP-1 drugs is gastrointestinal intolerance. Participants who experience intolerable adverse events prior to titration to 12 mg (≤12 mg) will be discontinued from the trial after being assessed by the investigator.

If the participant is intolerable to the titration dose of >12 mg, reduce the dose to the previous tolerable dose (≥12 mg). Other visits and test times remain unchanged, and the total dosing duration remains 30 weeks. For example, if the participant in the dose group (48 mg, Q2W) is intolerant from 36 mg to 48 mg, reduce the titration dose to 36 mg after evaluation by the investigator. Other visits and test times remain unchanged, and the total dosing duration remains 30 weeks.

During the study, the investigator can also communicate with the sponsor the dose reduction strategy initiated due to participant intolerance and the subsequent dose titration strategy based on the participants' tolerance.


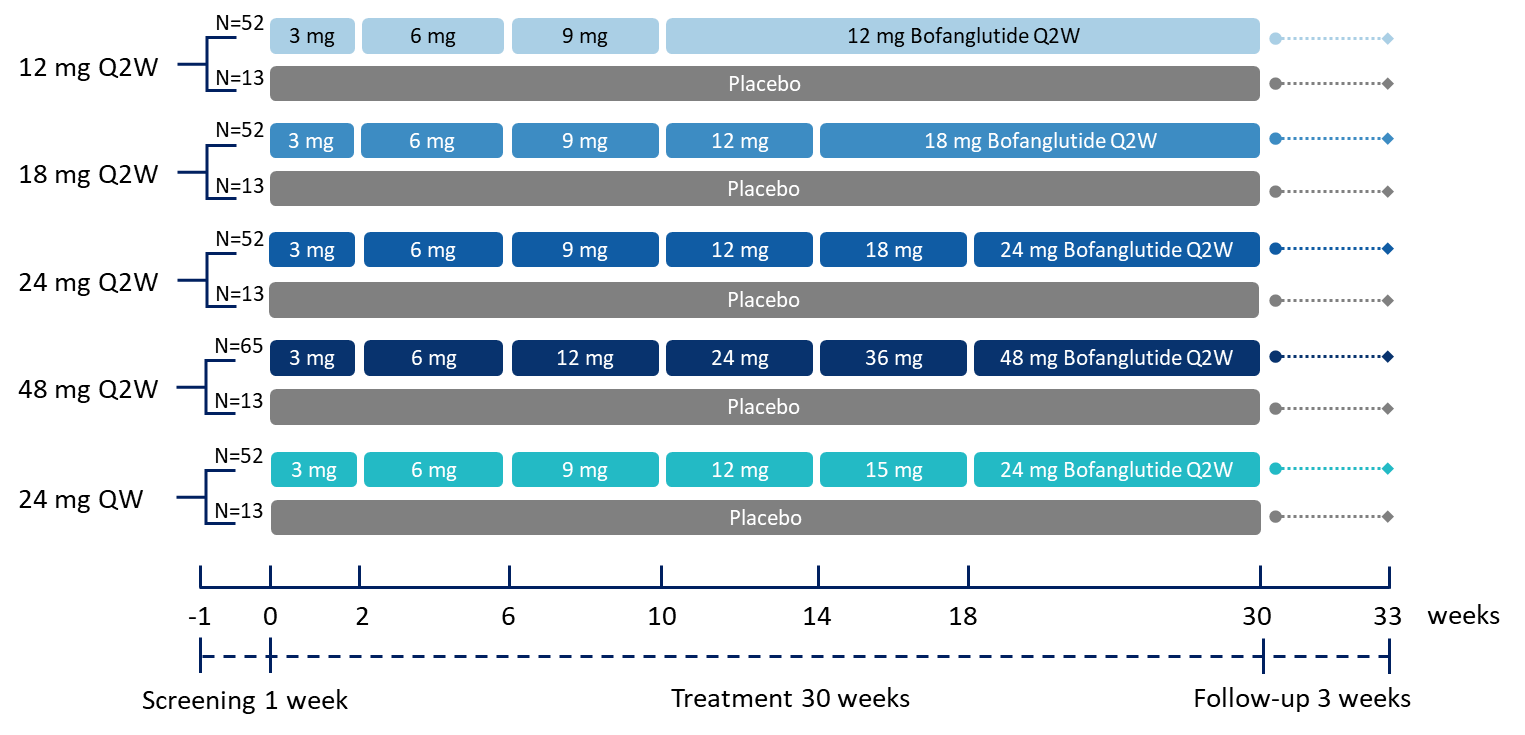


**Figure. S1.** The dose escalation schedules. QW= once weekly; Q2W= biweekly.

**
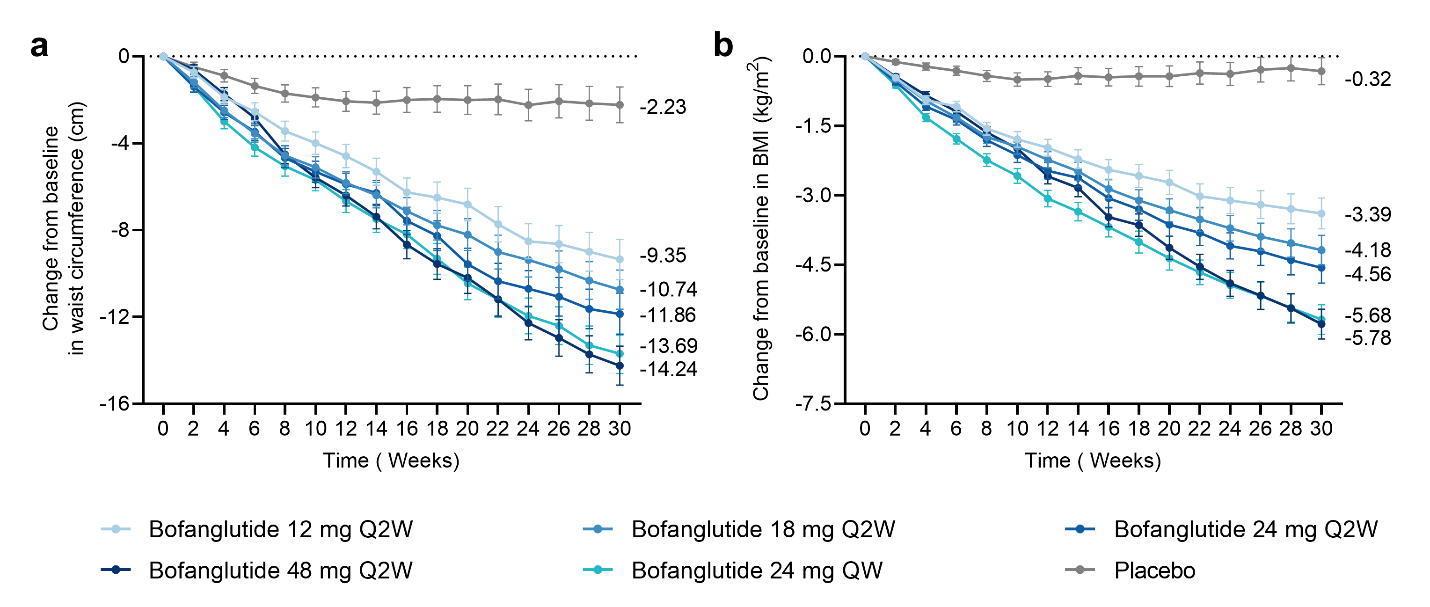
**

**Figure. S2.** Changes from baseline in waist circumference (A) and BMI (B) over time, from ANCOVA analysis, mITT population. Participants with missing value at week 30 were imputed using multiple imputation method. Symbols and error bars represent LSM and SE. Bofanglutide Q2W: 12 mg, n=52; 18 mg, n=53; 24 mg, n=52; 48 mg, n=64; bofanglutide 24 mg QW: n=53; placebo: n=66. BMI= body mass index; ANCOVA= analysis of covariance; SE= standard error; mITT= modified intention-to-treat; QW= once weekly; Q2W= biweekly.

**
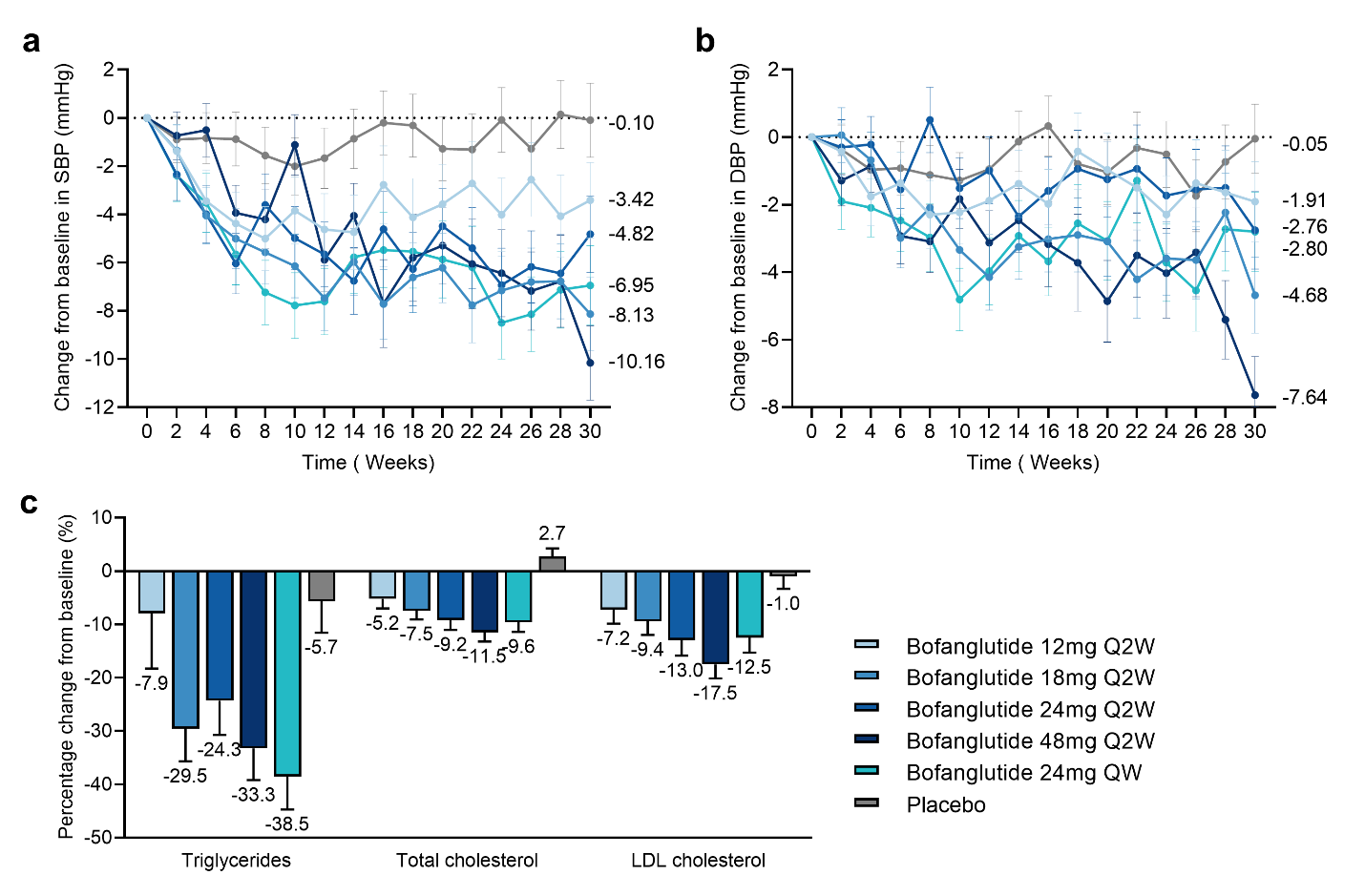
**

**Figure. S3.** Changes from baseline in SBP (A) and DBP (B) over time, and percentage change from baseline in lipids at week 30 (C), from ANCOVA analysis, mITT population. Participants with missing value at week 30 were imputed using multiple imputation method. Symbols and error bars represent LSM and SE. Bofanglutide Q2W: 12 mg, n=52; 18 mg, n=53; 24 mg, n=52; 48 mg, n=64; bofanglutide 24 mg QW: n=53; placebo: n=66. SBP= systolic blood pressure; DBP= diastolic blood pressure; LDL= low-density lipoprotein; ANCOVA= analysis of covariance; LSM= least squares mean; SE= standard error; mITT= modified intention-to-treat; QW= once weekly; Q2W= biweekly.

**
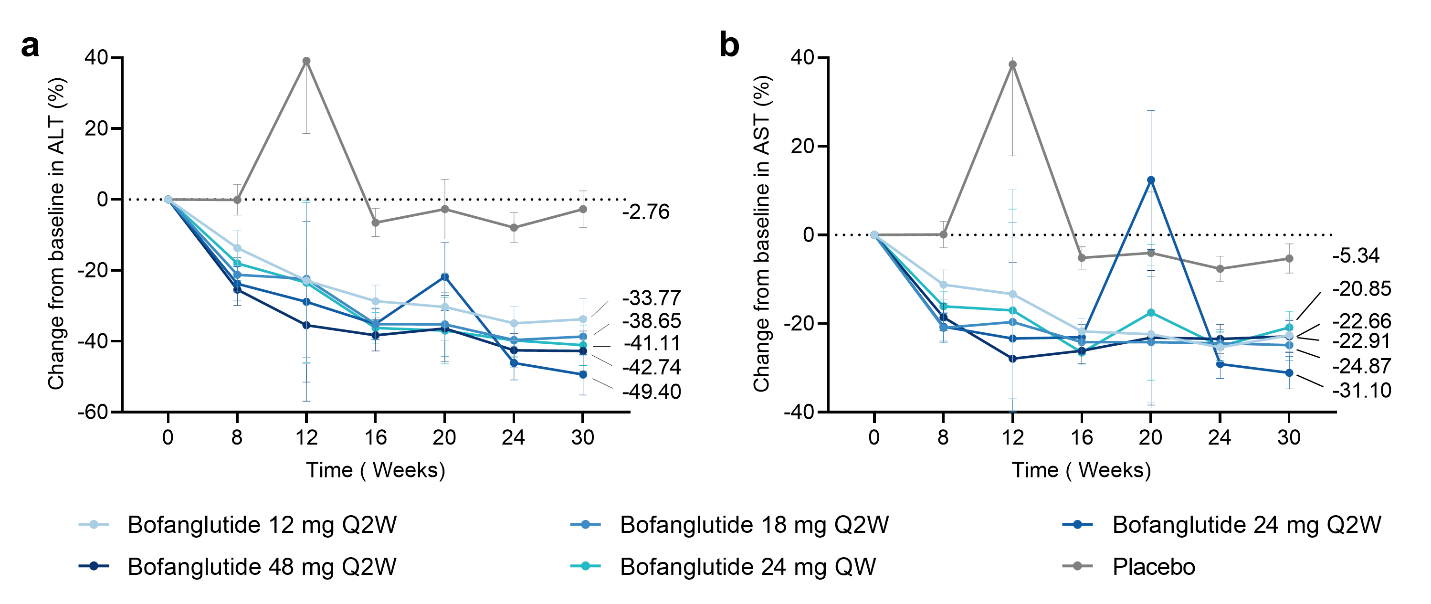
**

**Figure. S4.** Percentage change from baseline in ALT and AST levels over time, from ANCOVA analysis, mITT population.

Participants with missing value at week 30 were not imputed. Symbols and error bars represent LSM and SE. Bofanglutide Q2W: 12 mg, n=52; 18 mg, n=53; 24 mg, n=52; 48 mg, n=64; bofanglutide 24 mg QW: n=53; placebo: n=66. ANCOVA= analysis of covariance; LSM= least squares mean; SE= standard error; mITT= modified intention-to-treat; ALT= alanine aminotransferase; AST= aspartate aminotransferase; QW= once weekly; Q2W= biweekly.

*Note:* At week 12, a serious adverse event of liver failure with elevated ALT/AST was reported in one participant from the placebo group (see singular peaks in the placebo traces, Panels A/B), leading to trial discontinuation. The investigator considered this event possibly related to the investigational product. Additionally, the participant's history of fatty liver was identified as a potential contributing factor. The event resolved after appropriate treatment.

At week 20, an elevation in ALT/AST levels was observed in one participant from the bofanglutide 24 mg Q2W group (indicated by a single peak in the blue traces in Panels A/B). The participant had discontinued treatment (but remained in the trial) one month prior to the onset of this elevation. The investigator considered this event as not related to the investigational product. The elevation was transient and subsequently resolved to normal levels.


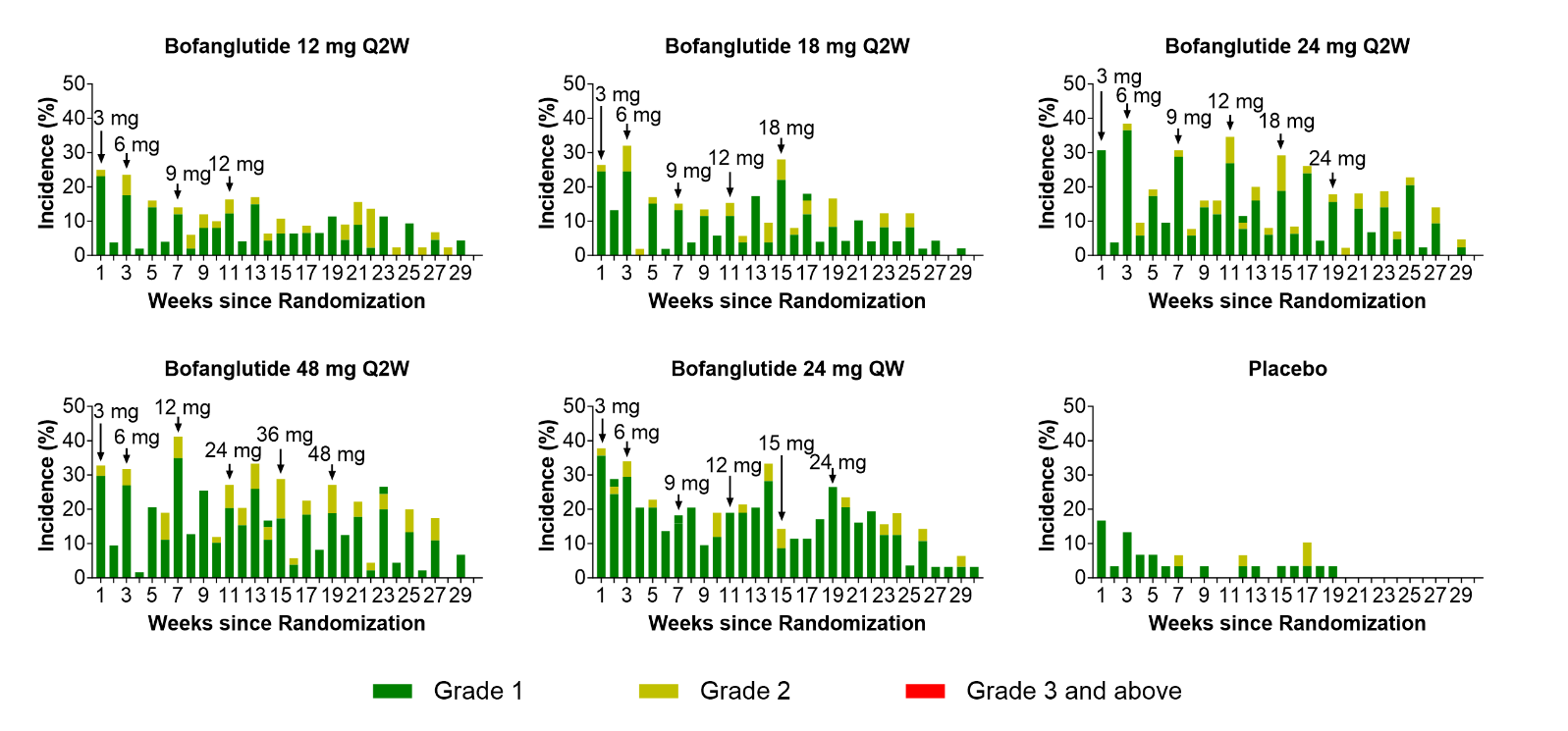


**Figure. S5.** By-week incidence of gastrointestinal adverse events over time, safety population. For each week, participants were counted once under each MedDRA preferred term and maximum severity. MedDRA= medical dictionary for regulatory activities; IP= investigational product; AE= adverse event; QW= once weekly; Q2W= biweekly.

Note: Gastrointestinal adverse events presented here are events within the corresponding System Organ Class “gastrointestinal disorders“, excluding the preferred term “decreased appetite”.


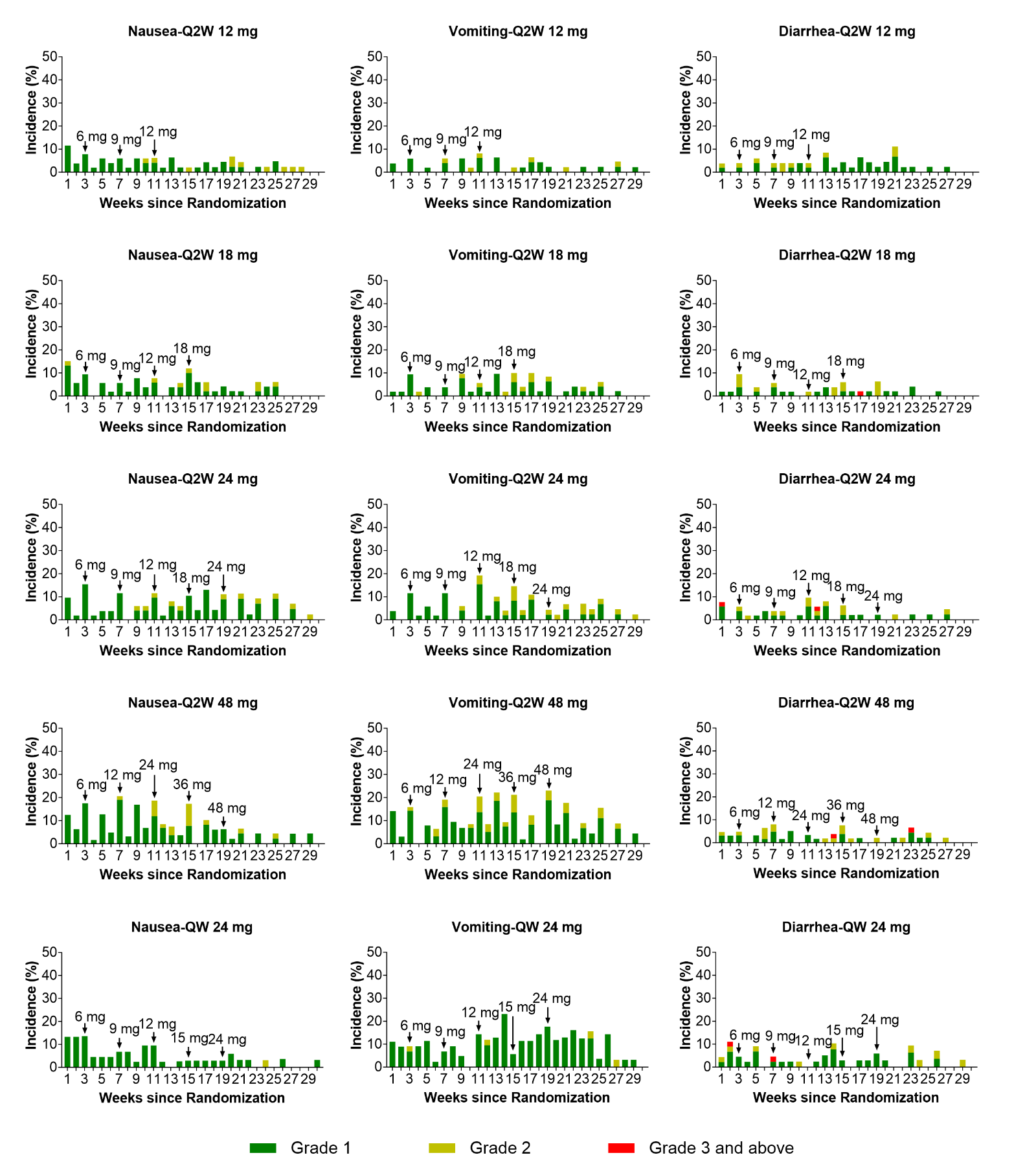


**Figure. S6.** By-week incidence of nausea, vomiting, and diarrhea over time, safety population. For each week, participants were counted once under each MedDRA preferred term and maximum severity. MedDRA= medical dictionary for regulatory activities; IP= investigational product; AE= adverse event; QW= once weekly; Q2W= biweekly.


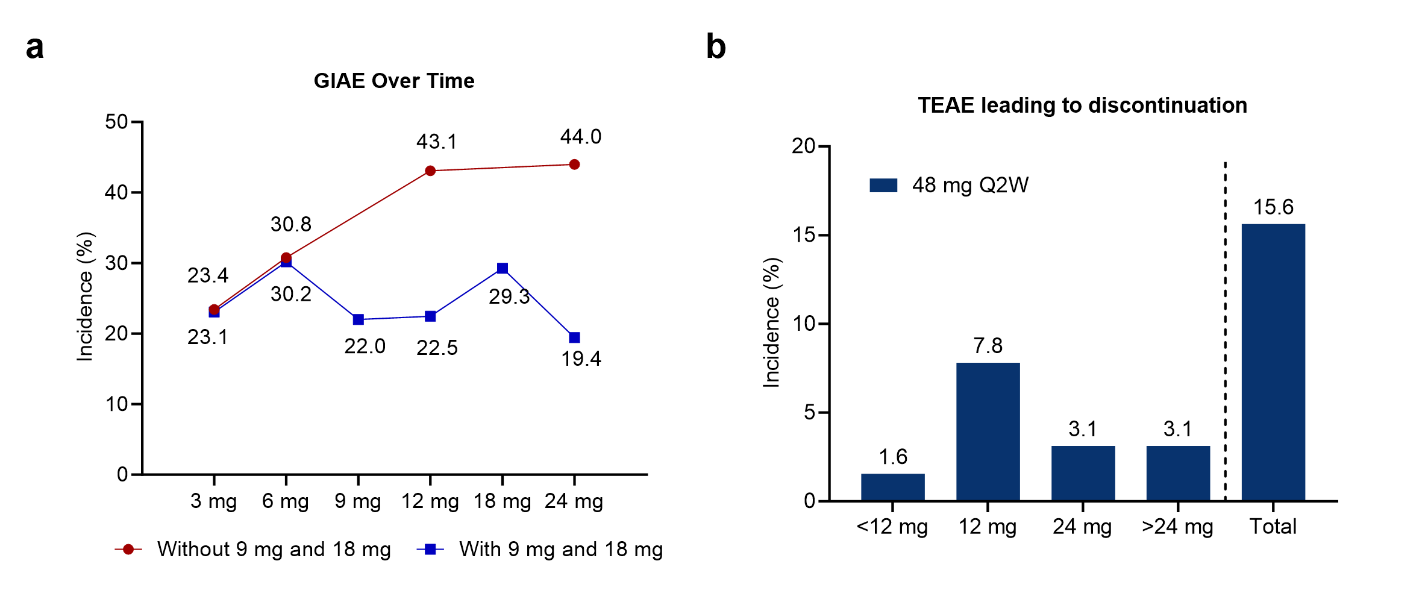


**Figure. S7.** The incidence of gastrointestinal adverse events of bofanglutide with or without 9 mg and 18 mg as the intermediate doses (A), and the incidence of TEAE leading to discontinuation at different doses (B). In panel B, “<12 mg”, “12 mg”, “24 mg”, and “>24 mg” refer to the last dose received before discontinuation.


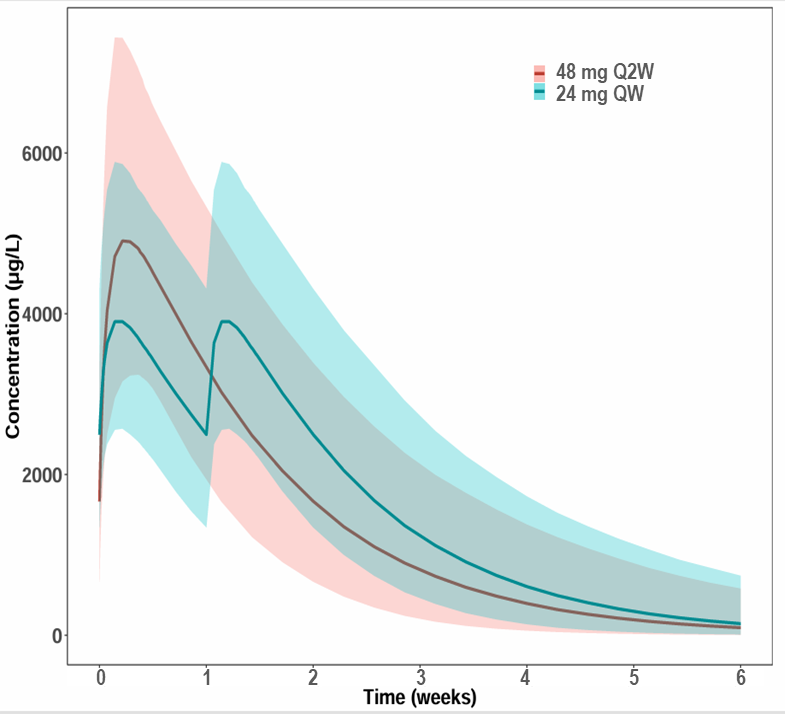


**Figure. S8.** Bofanglutide concentration-time curve in the last 2 weeks of treatment (steady-state) from population PK model, which was developed based on the PK data collected in the current study. Continuous lines represent the median and bands show the 95% prediction interval. QW= once weekly; Q2W= biweekly; PK= pharmacokinetics.

**Table S1.** Sex-specific demographic and baseline characteristics

| **Female participants**  **(N=155)** | **Bofanglutide Q2W** | | | | **Bofanglutide QW 24 mg (N=23)** | **Placebo (N=31)** |
| --- | --- | --- | --- | --- | --- | --- |
|  | **12 mg (N=20)** | **18 mg (N=26)** | **24 mg (N=20)** | **48 mg (N=35)** |  |  |
| Body weight (kg) | 82.33 (10.85) | 86.28 (15.92) | 87.10 (12.31) | 82.65 (11.21) | 83.62 (14.64) | 87.32 (16.31) |
| BMI (kg/m^2^) | 32.24 (3.82) | 32.20 (4.84) | 32.92 (3.29) | 31.86 (3.08) | 32.24 (4.88) | 33.25 (4.79) |
| BMI (kg/m^2^) [n (%)] | | | | | | |
| ≥24，＜28 | 1 (5.0) | 4 (15.4) | 1 (5.0) | 3 (8.6) | 4 (17.4) | 3 (9.7) |
| ≥28 | 19 (95.0) | 22 (84.6) | 19 (95.0) | 32 (91.4) | 19 (82.6) | 28 (90.3) |
| Waist circumference (cm) | 99.52 (9.62) | 101.06 (13.36) | 102.49 (8.91) | 99.20 (10.53) | 100.78 (12.71) | 103.05 (12.27) |
| HbA1c (%) | 5.57 (0.50) | 5.45 (0.41) | 5.53 (0.32) | 5.57(0.32) | 5.79 (0.59) | 5.54 (0.29) |
| Triglycerides (mmol/L) | 1.45 (0.48) | 1.72 (0.90) | 1.67 (0.49) | 1.59 (0.76) | 1.86 (1.05) | 1.25 (0.34) |
| Total cholesterol (mmol/L) | 4.69 (0.68) | 4.80 (0.55) | 5.12 (0.69) | 4.99 (1.36) | 4.96 (0.91) | 4.67 (0.89) |
| LDL cholesterol (mmol/L) | 3.09 (0.64) | 3.07 (0.58) | 3.34 (0.61) | 3.07 (0.81) | 3.07 (0.68) | 3.00 (0.81) |
| HDL cholesterol (mmol/L) | 1.15 (0.28) | 1.18 (0.21) | 1.20 (0.13) | 1.27 (0.23) | 1.17 (0.27) | 1.24 (0.27) |
| SBP (mmHg) | 117.3 (9.6) | 118.1 (10.3) | 119.1 (12.8) | 120.8 (10.1) | 114.7 (10.6) | 113.7 (8.7) |
| DBP (mmHg) | 80.0 (7.5) | 79.3 (8.5) | 80.0 (8.4) | 80.2 (8.6) | 78.9 (8.9) | 76.0 (5.9) |
| **Male participants**  **(N=185)** | **Bofanglutide Q2W** | | | | **Bofanglutide QW 24 mg (N=30)** | **Placebo (N=35)** |
|  | **12 mg (N=32)** | **18 mg (N=27)** | **24 mg (N=32)** | **48 mg (N=29)** |  |  |
| Body weight (kg) | 106.53 (20.88) | 99.49 (9.66) | 103.46 (16.99) | 109.61 (25.36) | 102.88 (17.07) | 105.39 (22.08) |
| BMI (kg/m^2^) | 34.65 (5.25) | 32.63 (3.27) | 33.58 (4.63) | 34.37 (6.24) | 33.69 (4.21) | 34.32 (5.23) |
| BMI (kg/m^2^) [n (%)] |  |  |  |  |  |  |
| ≥24，＜28 | 0 (0.0) | 1 (3.7) | 2 (6.3) | 5 (17.2) | 1 (3.3) | 2 (5.7) |
| ≥28 | 32 (100.0) | 26 (96.3) | 30 (93.8) | 24 (82.8) | 29 (96.7) | 33 (94.3) |
| Waist circumference (cm) | 112.81 (12.69) | 108.99 (6.88) | 111.53 (10.79) | 113.16 (13.95) | 110.51 (10.07) | 112.69 (12.31) |
| HbA1c (%) | 5.61 (0.32) | 5.51 (0.41) | 5.59 (0.47) | 5.51 (0.37) | 5.57 (0.38) | 5.70 (0.41) |
| Triglycerides (mmol/L) | 2.33 (1.03) | 2.27 (1.11) | 2.20 (0.83) | 1.76 (0.91) | 2.38 (1.16) | 2.17 (0.98) |
| Total cholesterol (mmol/L) | 4.97 (0.87) | 5.10 (0.88) | 5.05 (1.01) | 4.87 (0.77) | 5.17 (0.99) | 4.95 (0.85) |
| LDL cholesterol (mmol/L) | 3.16 (0.82) | 3.14 (0.68) | 3.26 (0.90) | 3.12 (0.68) | 3.30 (0.86) | 3.19 (0.74) |
| HDL cholesterol (mmol/L) | 1.04 (0.19) | 1.09 (0.28) | 1.10 (0.19) | 1.15 (0.24) | 1.07 (0.21) | 1.07 (0.16) |
| SBP (mmHg) | 125.8 (8.2) | 124.9 (11.6) | 125.3 (8.2) | 126.5 (10.0) | 123.9 (10.5) | 125.0 (8.7) |
| DBP (mmHg) | 83.0 (7.3) | 82.9 (9.3) | 82.3 (7.3) | 84.4 (7.5) | 83.5 (5.8) | 84.2 (6.5) |

Data are mean (SD) or n (%), mITT population. BMI= body mass index; HbA1c =glycated hemoglobin; HDL =high-density lipoprotein; LDL= low-density lipoprotein; SBP= systolic blood pressure; DBP= diastolic blood pressure; mITT= modified intention-to-treat; SD= standard deviation; QW= once weekly; Q2W= biweekly.

**Table S2.** Sensitivity and supporting analysis of body weight change

|  | **Bofanglutide Q2W** | | | | **Bofanglutide QW**  **24 mg (N=53)** | **Placebo**  **(N=66)** |
| --- | --- | --- | --- | --- | --- | --- |
|  | **12 mg (N=52)** | **18 mg (N=53)** | **24 mg (N=52)** | **48 mg (N=64)** |  |  |
| Sensitivity analysis (ANCOVA+LOCF; mITT population) | | | | | | |
| Percentage Change, % | -9.72 (0.94) | -12.50 (0.93) | -13.34 (0.94) | -15.09 (0.84) | -16.28 (0.93) | -1.16 (0.83) |
| ETD versus placebo | -8.57 (-11.03, -6.10)  *p*<0.001 | -11.35 (-13.80, -8.89)  *p*<0.001 | -12.19 (-14.65, -9.73)  *p*<0.001 | -13.94 (-16.27, -11.61)  *p*<0.001 | -15.12 (-17.57, -12.67)  *p*<0.001 |  |
| Supporting analysis 1 (MMRM; mITT population) | | | | | | |
| Percentage Change, % | -9.98 (0.94) | -12.74 (0.91) | -13.82 (0.93) | -17.57 (0.87) | -17.30 (0.93) | -0.93 (0.83) |
| ETD versus placebo | -9.05 (-11.50, -6.59)  *p*<0.001 | -11.81 (-14.22, -9.39)  *p*<0.001 | -12.89 (-15.32, -10.45)  *p*<0.001 | -16.64 (-19.00, -14.28)  *p*<0.001 | -16.37 (-18.81, -13.93)  *p*<0.001 |  |
| Supporting analysis 2 (MMRM; PPS population) | | | | | | |
| Number of participants | N=41 | N=49 | N=43 | N=45 | N=43 | N=53 |
| Percentage Change, % | -11.15 (1.04) | -13.22 (0.95) | -14.25 (1.01) | -17.29 (0.99) | -17.78 (1.01) | -0.99 (0.91) |
| ETD versus placebo | -10.17 (-12.86, -7.47) *p*<0.001 | -12.23 (-14.80, -9.67) *p*<0.001 | -13.27 (-15.93, -10.61) *p*<0.001 | -16.30 (-18.93, -13.68) *p*<0.001 | -16.80 (-19.45, -14.14) *p*<0.001 |  |

Data were presented as LSM (SE) for changes from baseline and LSM (95%CI) for ETD, from ANCOVA or MMRM analysis, mITT or PPS population. Statistical significance for the comparison between the bofanglutide and placebo groups at the end of treatment was assessed using *p* values. ANCOVA= analysis of covariance; mITT= modified intention-to-treat; PPS= per-protocol set; LOCF= last observation carried forward; MMRM= mixed model repeated measures; ETD= estimated treatment difference; LSM= least squares mean; SE= standard error; CI= confidence interval; QW= once weekly; Q2W= biweekly.

**Table S3.** Subgroup analysis of body weight change by baseline BMI and sex

|  | **Bofanglutide Q2W** | | | | **Bofanglutide QW**  **24 mg (N=53)** | **Placebo**  **(N=66)** |
| --- | --- | --- | --- | --- | --- | --- |
|  | **12 mg (N=52)** | **18 mg (N=53)** | **24 mg (N=52)** | **48 mg (N=64)** |  |  |
| Percentage Change in body weight at week 30, % (ANCOVA+ multiple imputation; mITT population) | | | | | | |
| **BMI** |  |  |  |  |  |  |
| ≥ 24 kg/m^2^,＜27 kg/m^2^（N=15） | -0.94 (-22.30, 20.41) | -14.02 (-24.48, -3.57) | -22.70 (-34.79, -10.61) | -17.74 (-23.57, -11.92) | -16.86 (-25.67, -8.04) | 3.29 (-3.86, 10.45) |
| ≥ 27 kg/m^2^,＜30 kg/m^2^（N=63） | -7.83 (-12.61, -3.05) | -15.45 (-19.45, -11.45) | -13.86 (-18.37, -9.35) | -16.01 (-20.41, -11.61) | -17.69 (-21.79, -13.59) | 1.85 (-2.14, 5.85) |
| ≥ 30 kg/m^2^（N=262） | -10.25 (-12.34, -8.15) | -11.59 (-13.75, -9.43) | -13.40 (-15.52, -11.27) | -16.04 (-18.19, -13.89) | -16.36 (-18.58, -14.14) | -2.07 (-4.00, -0.14) |
| **Sex** | | | | | | |
| Male (N=185) | -7.79 (-10.08, -5.49) | -11.85 (-14.33, -9.37) | -12.86 (-15.15, -10.57) | -15.03 (-17.61, -12.44) | -15.10 (-17.48, -12.72) | -1.11 (-3.29, 1.07) |
| Female (N= 155) | -12.65 (-16.00, -9.29) | -13.52 (-16.35, -10.68) | -14.85 (-18.08, -11.62) | -16.94 (-19.59, -14.29) | -18.82 (-21.91, -15.73) | -1.18 (-3.86, 1.50) |

Data were presented as LSM (95%CI) for changes from baseline, from ANCOVA analysis, mITT population. Participants with missing values at week 30 were imputed using the multiple imputation method. ANCOVA= analysis of covariance; mITT= modified intention-to-treat; LSM= least squares mean; CI= confidence interval; QW= once weekly; Q2W= biweekly; BMI= body mass index.

**Table S4.** Key secondary and exploratory efficacy endpoints at week 30

|  | **Bofanglutide Q2W** | | | | **Bofanglutide QW**  **24 mg (N=53)** | **Placebo (N=66)** |
| --- | --- | --- | --- | --- | --- | --- |
|  | **12 mg (N=52)** | **18 mg (N=53)** | **24 mg (N=52)** | **48 mg (N=64)** |  |  |
| **Secondary efficacy endpoints** | | | | | | |
| **Change from baseline in** | | | | | | |
| SBP, mmHg | -3.42 (1.56) | -8.13 (1.51) | -4.82 (1.58) | -10.16 (1.54) | -6.95 (1.64) | -0.10 (1.54) |
| ETD versus placebo | -3.31 (-7.47, 0.84)  *p*=0.059 | -8.03 (-12.17, -3.89)  *p*<0.001 | -4.71 (-8.84, -0.58)  *p*=0.013 | -10.05 (-14.12, -5.98)  *p*<0.001 | -6.85 (-11.16, -2.53)  *p*<0.001 |  |
| DBP, mmHg | -1.91 (1.19) | -4.68 (1.12) | -2.76 (1.15) | -7.64 (1.14) | -2.80 (1.17) | -0.05 (1.02) |
| ETD versus placebo | -1.86 (-4.95, 1.24)  *p*=0.120 | -4.63 (-7.55, -1.70)  *p*<0.001 | -2.71 (-5.71, 0.29)  *p*=0.038 | -7.59 (-10.52, -4.67)  *p*<0.001 | -2.75 (-5.82, 0.32)  *p*=0.040 |  |
| HbA1c, % | -0.29 (0.04) | -0.28 (0.04) | -0.33 (0.04) | -0.45 (0.04) | -0.47 (0.05) | 0.07 (0.04) |
| ETD versus placebo | -0.35 (-0.47, -0.24)  *p*<0.001 | -0.35 (-0.46, -0.23)  *p*<0.001 | -0.40 (-0.52, -0.28)  *p*<0.001 | -0.52(-0.64, -0.39)  *p*<0.001 | -0.53 (-0.66, -0.41)  *p*<0.001 |  |
| FPG, mmol/L | -0.17 (0.07) | -0.06 (0.07) | -0.18 (0.07) | -0.40 (0.07) | -0.32 (0.07) | 0.21 (0.07) |
| ETD versus placebo | -0.37 (-0.56,-0.18)  *p*<0.001 | -0.27 (-0.47,-0.07)  *p*=0.008 | -0.38 (-0.59,-0.17)  *p*<0.001 | -0.61 (-0.82,-0.40)  *p*<0.001 | -0.53 (-0.73,-0.32)  *p*<0.001 |  |
| Fasting insulin, μIU/mL | -3.28 (3.05) | -0.97 (3.11) | -5.58 (3.03) | -7.84 (2.78) | -7.15 (3.04) | 6.42 (3.48) |
| ETD versus placebo | -9.70 (-18.57, -0.83)  *p*=0.032 | -7.39 (-16.50, 1.72)  *p*=0.111 | -12.00 (-21.22, -2.78)  *p*=0.011 | -14.26 (-23.40, -5.12)  *p*=0.002 | -13.57 (-22.78, -4.36)  *p*=0.004 |  |
| HOMA-IR | -0.89 (1.03) | 0.27 (1.06) | -1.58 (1.03) | -1.96 (0.94) | -1.82 (1.03) | 2.26 (1.21) |
| ETD versus placebo | -3.15 (-6.23, -0.08)  *p*=0.044 | -1.99 (-5.14, 1.16)  *p*=0.214 | -3.83 (-7.00, -0.68)  *p*=0.018 | -4.22 (-7.33, -1.10)  *p*=0.008 | -4.08 (-7.23, -0.93)  *p*=0.011 |  |
| **Percentage change from baseline in** | | | | | | |
| Total cholesterol, % | -5.24 (1.78) | -7.47 (1.61) | -9.22 (1.81) | -11.47 (1.75) | -9.63 (1.77) | 2.72 (1.53) |
| ETD versus placebo | -7.96 (-12.40, -3.52)  *p*<0.001 | -10.19 (-14.55, -5.84)  *p*<0.001 | -11.94 (-16.57, -7.31)  *p*<0.001 | -14.19 (-18.63, -9.75)  *p*<0.001 | -12.35 (-17.00, -7.69)  *p*<0.001 |  |
| LDL cholesterol, % | -7.24 (2.64) | -9.44 (2.52) | -12.96 (2.89) | -17.51 (2.62) | -12.48 (2.82) | -1.00 (2.36) |
| ETD versus placebo | -6.24 (-13.06, 0.59)  *p*=0.073 | -8.44 (-15.20, -1.68)  *p*=0.014 | -11.96 (-19.28, -4.64)  *p*=0.001 | -16.51 (-23.35, -9.67)  *p*<0.001 | -11.48 (-18.77, -4.19)  *p*=0.002 |  |
| HDL cholesterol, % | 2.13 (2.31) | 5.82 (2.21) | -3.91 (2.28) | -4.76 (2.24) | 3.14 (2.46) | 3.73 (2.02) |
| ETD versus placebo | -1.60 (-7.48, 4.28)  *p*=0.594 | 2.09 (-3.74, 7.92)  *p*=0.483 | -7.64 (-13.56, -1.73)  *p*=0.011 | -8.49 (-14.47, -2.50)  *p*=0.006 | -0.59 (-6.86, 5.69)  *p*=0.854 |  |
| Triglycerides, % | -7.90 (10.39) | -29.54 (6.13) | -24.32 (6.41) | -33.26 (5.94) | -38.54 (6.17) | -5.69 (5.84) |
| ETD versus placebo | -2.21 (-25.30, 20.87)  *p*=0.849 | -23.84 (-40.23, -7.46)  *p*=0.004 | -18.63 (-36.05, -1.21)  *p*=0.036 | -27.57 (-43.89, -11.24) *p*=0.001 | -32.85 (-49.47, -16.22)  *p*<0.001 |  |
| **Exploratory efficacy endpoints** | | | | | | |
| Number of participants | N=44 | N=49 | N=45 | N=46 | N=45 | N=55 |
| **Change from baseline in** | | | | | | |
| Serum uric acid, μmol//L | -86.95 (8.77) | -91.10 (8.31) | -101.70 (8.74) | -74.96 (8.62) | -100.25 (8.67) | -35.31 (7.84) |
| ETD versus placebo | -51.63 (-74.80, -28.47)  *p*<0.001 | -55.79 (-78.29, -33.29) *p*<0.001 | -66.38 (-89.50, -43.27)  *p*<0.001 | -39.64 (-62.59, -16.70) *p*<0.001 | -64.94 (-87.96, -41.92)  *p*<0.001 |  |
| **Percentage change from baseline in** | | | | | | |
| ALT, % | -33.77 (5.78) | -38.65 (5.48) | -49.40 (5.72) | -42.74 (5.65) | -41.11 (5.70) | -2.76 (5.16) |
| ETD versus placebo | -31.01 (-46.23, -15.80)  *p*<0.001 | -35.89 (-50.74, -21.04) *p*<0.001 | -46.65 (-61.77, -31.52)  *p*<0.001 | -39.98 (-55.09, -24.88) *p*<0.001 | -38.35 (-53.52, -23.19)  *p*<0.001 |  |
| AST, % | -22.66 (3.69) | -24.87 (3.50) | -31.10 (3.6586) | -22.91 (3.62) | -20.85 (3.64) | -5.34 (3.30) |
| ETD versus placebo | -17.32 (-27.05, -7.59)  *p*<0.001 | -19.54 (-29.01, -10.07) *p*<0.001 | -25.77 (-35.45, -16.08)  *p*<0.001 | -17.58 (-27.22, -7.93)  *p*<0.001 | -15.51 (-25.18, -5.85)  *p*=0.002 |  |

Data are LSM (SE) for change from baseline and LSM (95% CI) for ETD versus placebo at week 30 from ANCOVA analysis, mITT population. Participants with missing values at week 30 were imputed using the multiple imputation method (with the exception of exploratory endpoints, for which no imputation was performed). Statistical significance for the comparison between the bofanglutide and placebo groups at the end of treatment was assessed using *p* values. ALT= alanine aminotransferase; AST= aspartate aminotransferase; BMI= body-mass index; FPG= fasting plasma glucose; HbA1c= glycated hemoglobin; HOMA-IR= homeostatic model assessment of insulin resistance; SBP= systolic blood pressure; DBP= diastolic blood pressure; HDL= high-density lipoprotein; LDL= low- density lipoprotein; LSM= least squares mean; SE= standard error; CI= confidence interval; ETD= estimated treatment difference; mITT= modified intention-to-treat; ANCOVA= analysis of covariance; QW= once weekly; Q2W= biweekly.

**Table S5.** IWQoL-Lite-CT and SF-36 scores at week 30

|  | **Bofanglutide Q2W** | | | | **Bofanglutide QW**  **24 mg (N=53)** | **Placebo (N=66)** |
| --- | --- | --- | --- | --- | --- | --- |
|  | **12 mg (N=52)** | **18 mg (N=53)** | **24 mg (N=52)** | **48 mg (N=64)** |  |  |
| IWQoL-Lite-CT, change from baseline in | | | | | | |
| Total scores | 10.61 (1.65) | 12.57 (1.52) | 12.68 (1.55) | 11.05 (1.73) | 8.45 (1.62) | 8.03 (1.38) |
| ETD versus placebo | 2.58 (-1.63, 6.78) | 4.53 (0.52, 8.54) | 4.65 (0.59, 8.70) | 3.01 (-1.37, 7.40) | 0.42 (-3.73, 4.56) |  |
| Physical function scores | 8.8 (1.68) | 11.4 (1.54) | 11.4 (1.55) | 8.9 (1.78) | 7.5 (1.62) | 6.6 (1.39) |
| ETD versus placebo | 2.3 (-2.0, 6.6) | 4.9 (0.8, 8.9) | 4.9 (0.8, 9.0) | 2.4 (-2.1, 6.8) | 1.0 (-3.2, 5.1) |  |
| SF-36, change from baseline in | | | | | | |
| PF_NBS | 2.36 (0.56) | 3.08 (0.53) | 3.17 (0.53) | 2.92 (0.55) | 1.86 (0.54) | 1.01 (0.49) |
| ETD versus placebo | 1.35 (-0.13, 2.83) | 2.07 (0.66, 3.47) | 2.16 (0.74, 3.58) | 1.91 (0.44, 3.38) | 0.86 (-0.61, 2.32) |  |
| Physical component summary | 2.99 (0.57) | 3.77 (0.544) | 3.23 (0.55) | 2.73 (0.54) | 1.93 (0.57) | 1.67 (0.50) |
| ETD versus placebo | 1.32 (-0.18, 2.82) | 2.10 (0.65, 3.55) | 1.56 (0.10, 3.02) | 1.06 (-0.40, 2.52) | 0.26 (-1.24, 1.77) |  |
| Mental component summary | 1.46 (0.74) | 1.75 (0.71) | 1.87 (0.73) | 0.64 (0.74) | 1.12 (0.73) | 1.28 (0.64) |
| ETD versus placebo | 0.18 (-1.74, 2.11) | 0.47 (-1.41, 2.36) | 0.59 (-1.29, 2.47) | -0.64 (-2.56, 1.27) | -0.16 (-2.07, 1.75) |  |

Data are LSM (SE) for change from baseline and LSM (95% CI) for ETD versus placebo at week 30 from ANCOVA analysis, mITT population. Participants with missing values at week 30 were imputed using the multiple imputation method. IWQOL-Lite-CT= Impact of Weight on Quality of Life-Lite Clinical Trials Version; SF-36= Short Form-36 Health Survey; PF_NBS= physical functioning norm-based scoring; LSM= least squares mean; SE= standard error; CI= confidence interval; ETD= estimated treatment difference; mITT= modified intention-to-treat; ANCOVA= analysis of covariance; QW= once weekly; Q2W= biweekly.

**Table S6.** Pharmacokinetics endpoint

|  | **Bofanglutide Q2W** | | | | **Bofanglutide QW**  **24 mg (N=53)** |
| --- | --- | --- | --- | --- | --- |
|  | **12 mg (N=52)** | **18 mg (N=53)** | **24 mg (N=52)** | **48 mg (N=64)** |  |
| C_max,ss_ (ng/mL) | 1236.9 (238.07) | 1765.9 (481.3) | 2127.5 (719.6) | 4445.8 (903.4) | 4766.4 (2162.4) |
| AUC_0–τ,ss_ (h*ng/mL) | 257,146.5 (59,187.8) | 396,900.0 (99,298.1) | 479,088.0 (154,885.4) | 964,657.9 (155,176.8) | 645,662.1 (264,131.3) |
| AUC_0-168h_ (h*ng/mL) | 162,593.7 (31,659.8) | 246,648.7 (63,458.2) | 299,100.0 (96,909.5) | 599491.9 (100,910.3) | 645662.1 (264,131.3) |
| T_max,ss_ (h) | 24.0 | 48.0 | 48.0 | 36.0 | 36.0 |
| t_1/2_ (h) | 156.2 (16.7) | 159.9 (44.0) | 169.2 (29.7) | 162.9 (20.2) | 176.7 (41.0) |

Data were presented as mean (SD) or median, mPKPS population, including all participants who were randomized, received the IMP at least once, underwent intensive blood sampling and had at least one PK evaluation. The dosing interval τ was 336 h in the Q2W group and 168 h in the QW group. SD= standard deviation; SS= steady state; IP= investigational product; mPKPS=micro pharmacokinetic parameter set; QW= once weekly; Q2W= biweekly.

**Table S7.** Relationship between PK parameters and dose after last dose of bofanglutide (Q2W dosing frequency)

| **PK parameters** | **β** | **SE** | **95% CI** | **Acceptance interval** |
| --- | --- | --- | --- | --- |
| C_max,ss_ | 0.96 | 0.07 | 0.84~1.07 | 0.84~1.16 |
| AUC_0–τ,ss_ | 0.98 | 0.07 | 0.87~1.09 | 0.84~1.16 |

Data were from mPKPS population. The dosing interval τ was 336 h in the Q2W group. SE= standard error; mPKPS= micro pharmacokinetic parameter set; SS= steady state; CI= confidence interval; Q2W= biweekly.
