# Supplementary material for: Efficacy and safety of bofanglutide, a GLP-1 receptor agonist, in Chinese adults with overweight or obesity: a randomized, double-blind, placebo-controlled phase 2b trial
Source: Signal Transduct Target Ther. 2026 Feb 27;11:73. doi: 10.1038/s41392-026-02586-8 (PMC12948953; doi:10.1038/s41392-026-02586-8)
Supplement: Supplementary file 2 — Clinical trial protocol [file 41392_2026_2586_MOESM2_ESM.pdf]

Protocol GL-GLP-CH2005

A Multicenter, Randomized, Controlled Phase *II* Clinical Study to Evaluate the Efficacy and Safety of GZR18 Injection in Chinese Obese/Overweight Patients

Phase *II*

Gan & Lee Pharmaceuticals Co., Ltd.

No. 8 Nanfeng West 1st Street, Huoxian, Tongzhou District, Beijing

China

Confidentiality

This document is owned by **Gan & Lee Pharmaceuticals Co., Ltd.** and may not be distributed, copied, published or otherwise used in whole or in part without express permission from **Gan & Lee Pharmaceuticals Co., Ltd.**

Sponsor Signature Page

This protocol has been critically reviewed by the sponsor. It contains information consistent with the following:

- Current risk-benefit assessment of the investigational product (IP).
- *Declaration of Helsinki*, ICH GCP and Chinese GCP.

The investigator is provided with details of any significant or new findings related to IP treatment, including adverse events (AEs).

Signatures of the sponsor: ______________________ Date: ____________________

[redacted]

Signatures of the sponsor: ______________________ Date: ____________________

[redacted]

Signatures of the sponsor: ______________________ Date: ____________________

[redacted]

Signature Page for Investigator

I have read the protocol and its attachments for clinical study GL-GLP-CH2005. I agree to:

1. Conduct this clinical study in accordance with the design and detailed provisions of this protocol and its attachments;
2. Not to make modifications to the study plan specified in the study protocol, i.e. not to violate/deviate from the protocol, before obtaining the written consent from the sponsor and the Ethics Committee, unless the modification is intended to protect the subjects form direct harms;
3. Protect the rights, safety and welfare of the subjects;
4. Supervise the study tasks if they are delegated to others;
5. Manage all investigational drug s provided by the sponsor and maintain records of drug disposition;
6. Conduct this study in accordance with all applicable regulations, requirements of Ethics Committee, ICH GCP and provisions of Chinese GCP;
7. Obtain approval for the protocol and all written materials provided to the subjects prior to the start of the study;
8. Obtain informed consent from all enrolled subjects prior to initiation of any study-related procedures or administration of investigational drug s at the site;
9. Maintain records of each subject’s participation in the study, as well as all data generated under this protocol;
10. Keep all information related to this study confidential.

Site name：

Address：

Investigator Name：

Signature for Principal Investigator： ______________**Date**__________________

Signature Page of the Contract Research Organization（CRO）

I have read and confirm the protocol and its attachments for clinical study GL-GLP-CH2005. I agree to conduct the study in accordance with this protocol.

I agree to：

- Strictly follow the protocol, current *ICH GCP* and GCP guidelines, as well as applicable regulations and guidelines for clinical monitoring of this trial.
- Maintain all data and information provided by Gan & Lee Pharmaceuticals Co., Ltd. in accordance with confidentiality requirements, and when such data and information is to be presented to the Independent Ethics Committee (IEC), it must be marked as confidential.

Contract research organization： [redacted]

Address: [redacted]

Name of the Project Director:：

Signature of the Project Director： ______________ **Date：** _________________

Signature Page of Biostatistics Facility

We have read and understood the study protocol and its attachments for clinical study GL-GLP-CH2005, and agree to comply with all the provisions listed in the protocol.

I agree to:

- Strictly follow the protocol, current *ICH GCP* and GCP guidelines, as well as applicable regulations and guidelines for data management and statistical analysis of this trial.
- Maintain all data and information provided by Gan & Lee Pharmaceuticals Co., Ltd. in accordance with confidentiality requirements, and when such data and information is to be presented to the Independent Ethics Committee (IEC), it must be marked as confidential.

Biostatistics Facility: [redacted]

Address: [redacted]

Name of the Project Director::

Signature of the Project Director: _______________ **Date:** __________________

Research Contact Information

Sponsor

Name	Gan & Lee Pharmaceuticals Co., Ltd.
Address	No.8 Nanfeng West 1st Street, Huoxian, Tongzhou District, Beijing
Postal code	101109

Medical expert/medical director of the sponsor

Name	[redacted]
Email	[redacted]
Work address	[redacted]

Clinical project leader of the sponsor

Name	[redacted]
Email	[redacted]
Work address	[redacted]

Contract research organization (CRO)

Name	[redacted]
Address	[redacted]
Postal code	[redacted]

CRO project manager

Name	[redacted]
Tel	[redacted]
Email	[redacted]
Work address	[redacted]

CRO clinical data manager

Name	[redacted]
Tel	[redacted]
Email	[redacted]
Work address	[redacted]

CRO clinical trial statistician

Name	[redacted]
Tel	[redacted]
Email	[redacted]
Work address	[redacted]

Additional research contact information is provided in a separate document.

Table of Contents

Sponsor Signature Page I
Signature Page for Investigator II
Signature Page of the Contract Research Organization（CRO） III
Signature Page of Biostatistics Facility IV
Research Contact Information V
Table of Contents 1
List of Tables 5
Abbreviations 6
1. Protocol Abstract 9
1.1 Summary 9
1.2 Study flowchart 28
1.3 PK blood sampling schedule 35
1.4 Study Design Chart 39
2. Introduction 40
2.1 Background and epidemiology of the target disease 40
2.2 Background information of this product 41
2.3 Risk/benefit assessment 41
3. Objectives and endpoints 43
3.1 Objectives 43
3.2 Endpoints 43
4. Trial design 45
4.1 General design 45
4.1.1 Study duration for each subject 45
4.1.2 Number of planned subjects and research sites 45
4.2 Justification for selection of doses 45
4.2.1 Summary of the design basis for the trial protocol 46
5. Subject population 48
5.1 Inclusion criteria 48
5.2 Exclusion criteria 48

5.3 Withdrawal criteria 51
5.4 Study termination criteria 53
5.5 Subject dropouts or lost to follow-up 53
5.6 Lifestyle concerns 54
5.7 Screen failure 54
5.8 Recruitment and retention strategy 54
6. Study intervention 55
6.1 Dose and administration 55
6.2 Drug preparation 57
6.2.1 Drug information 57
6.2.2 Appearance and packaging 57
6.2.3 Label 57
6.2.4 Storage and stability of investigational drugs 57
6.2.5 Drug inventory 58
6.3 Methods to reduce bias: Randomization and blinding 58
6.3.1 Randomization 58
6.3.2 Blinding 59
6.3.3 Emergency unblinding 60
6.4 Intervention compliance 60
6.5 Concomitant therapy 61
6.5.1 Prohibited concomitant treatment (drug and non-drug therapies) during the trial 62
6.5.2 Allowable concomitant treatment (drug and non-drug therapies) during the trial 62
7. Assessments and procedure in the clinical trial 63
7.1 Safety assessments 63
7.2 Efficacy assessment 66
7.3 Pharmacokinetic evaluation 66
7.4 Adverse event 67
8. Study Management and Administration 75
8.1 Protocol compliance 75
8.2 Monitoring 75

8.2.1 Definition of source data 76
8.2.2 Source data verification 77
8.3 Data processing 77
8.3.1 Case report form completion 77
8.3.2 Database entry and consistency verification 78
8.3.3 Subject screening and enrollment log/subject identification code list 79
8.4 Study termination 79
8.5 Archiving and data preservation 80
8.6 Audit and inspection 80
8.7 Good Clinical Practice 80
8.8 Biological samples 81
9. Statistics 82
9.1 Definition of analysis sets 82
9.2 General statistical consideration 82
9.2.1 Demographic characteristics of subjects 83
9.2.2 Compliance and statistical analysis of concomitant medication 83
9.3 Planned safety analysis 83
9.4 Scheduled efficacy analysis 84
9.5 Planned PK analysis 85
9.6 Handling of protocol deviations 85
9.7 Handling of dropouts or missing data 86
9.8 Determination of sample size 86
9.9 Statistical software 86
10. Regulation, Ethics, and Study Regulatory Requirements 86
10.1 Regulation 87
10.2 Informed consent 87
10.3 Subject ID card 88
10.4 Institutional Review Board (IRB) and Independent Ethics Committee (IEC) 88
10.5 Privacy of subjects 89
10.6 Protocol amendment 89
11. Finance, Insurance and Publication 90

12. References 91

List of Tables

Table 1-1 PK blood sampling schedule-once every two weeks （dose group 1 to 4）（based on PK blood sampling time window） 35

Table 1-2 PK blood sampling schedule-once-weekly（dose group 5）（based on PK blood sampling time window） 37

Table 4-1 48 mg dose-escalation multiplicity of GZR18 injection in phase II (GL-GLP-CH2005)...47

Table 6-1 Dosing design 55

Table 6-2 Study drug information...57

Table 6-3 Random number allocation...59

Abbreviations

Abbreviations	Full description
ADA	Anti-drug antibody
ADME	Absorption, Distribution, Metabolism and Excretion
ADR	Adverse drug reaction
AE	Adverse event
AESI	Adverse events of special interest
AUC	Area under the curve
ALB	Albumin
ALCOA	Attributable, Legible, Contemporaneous, Original and Accurate
ALP	Alkaline phosphatase
ALT	Alanine aminotransferase
APTT	Activated partial thromboplastin time
AST	Aspartate aminotransferase
BMI	Body Mass Index
BUN	Blood urea nitrogen
CA	Competent authority
CABG	Coronary artery bypass grafting
CKD-EPI	Chronic Kidney Disease - Epidemiology Collaboration
COA	Certificate of analysis
CL/F	Clearance rate/Frel
C_{max}	Maximum plasma concentration
CRF	Case report form
CPM	Clinical Project Manager
CRA	Clinical research associate
CRO	Contract research organization
CV	Coefficient of variation
Cr	Creatinine
DBIL	Direct bilirubin
DEM	Data Evaluation Meeting
DIO	Diet-induced obesity
DPP-4	Dipeptidyl peptidase-4
DSUR	Development Safety Update Report
eCRF	Electronic case report form
ECG	Electrocardiogram
EDC	Electronic Data Capture
eGFR	Estimated glomerular filtration rate
ET	Early termination
FAS	Full analysis set
FDA	Food and Drug Administration
FT3	Free tri-iodothyronine
FT4	Free tetra-iodothyronine/free thyroxine
GCGR	Glucagon Receptor
GCP	Good Clinical Practice

GLP-1	Glucagon-like peptide-1
GLP-1 RA	Glucagon-like peptide-1 receptor agonist
GIPR	Glucose-dependent insulinotropic polypeptide receptor
HDL-C	High density lipoprotein cholesterol
HIV	Human Immunodeficiency Virus
HSA	Human serum albumin
IB	Investigator's Brochure
ICH	International Council for Harmonization (of Technical Requirements for Registration of Pharmaceuticals for Human Use)
INR	International normalized ratio
IP	Investigational Product
IRB/IEC	Institutional Review Board/Independent Ethics Committee
ISH	International Society of Hypertension
ITT	Intent-to-Treat Set
IVRS/IWRS	Interactive Voice Response System/Interaction Web Response System
LDL-C	Low density lipoprotein cholesterol
MedDRA	Medical Dictionary for Regulatory Activities
MEN	Multiple endocrine neoplasia syndrome
MRT	Mean residence time
MTD	Maximum tolerated dose
NAb	Neutralizing antibody
NCI-CTCAE	National Cancer Institute - Common Terminology Criteria for Adverse Events
NEP	Neutral endopeptidase
NOAEL	No-observed-adverse-effect level
NYHA	New York Heart Association
OGTT	Oral glucose tolerance test
PD	Pharmacodynamics
PDPS	Pharmacodynamic analysis set
PHQ-9	Patient Health Questionnaire-9
PK	Pharmacokinetics
PKPS	Pharmacokinetic analysis set
PPS	Per Protocol Set
PT	Preferred term
PT	Prothrombin time
PV	Pharmacovigilance
SAE	Serious adverse event
SAP	Statistical Analysis Plan
SD	Sprague-Dawley
SD	Standard Deviation
SGLT-2	Sodium-glucose cotransporter-2
SNRI	Serotonin noradrenaline reuptake inhibitor
SOC	System Organ Class
SOP	Standard operating procedure
SS	Safety analysis set
SSRI	Selective serotonin reuptake inhibitor

SUSAR	Suspected unexpected serious adverse reaction
$t_{1/2}$	Half-life
TBIL	Total bilirubin
TC	Total cholesterol
TEAE	Treatment Emergent Adverse Event
TG	Triglycerides
t_{lag}	Lag Time
t_{max}	The Time to Maximum Plasma Concentration
TSH	Thyroid-stimulating hormone
TT	Thrombin time
TZD	Thiazolidinediones
ULN	Upper limit of normal
Vz/F	Extravascular apparent distribution volume corrected by bioavailability
WHO	World Health Organization

1. Protocol Abstract

1.1 Summary

**Study title**	A Multicenter, Randomized, Controlled *Phase II* Clinical Study to Evaluate the Efficacy and Safety of GZR18 Injection in Chinese Obese/Overweight Patients
**Sponsor**	Gan & Lee Pharmaceuticals Co., Ltd.
**Registration classification**	Category 1 Therapeutic Biological Product
**Protocol No.**	GL-GLP-CH2005
**Study phase**	Phase II
**Leader facility of the clinical study**	Peking University People's Hospital
**Principal investigator**	Professor Ji Linong
**Study subjects**	Adult obese/overweight patients with poor weight control after lifestyle intervention
**Study objectives**	**Primary objective：**<br>• To evaluate the weight loss effect of GZR18 injection in adult obese/overweight subjects after 30 weeks of treatment.<br>**Secondary objectives：**<br>• To evaluate the safety and tolerability of GZR18 injection.<br>• To evaluate the pharmacokinetics of GZR18 injection.<br>• To evaluate the immunogenicity of GZR18 injection.<br>• To evaluate the effects of GZR18 injection on glucose metabolism, cardiovascular disease risk factors, and weight-related quality of life reported by patient.<br>**Exploratory objectives：**<br>• To evaluate the effect of GZR18 injection on blood uric acid and fatty liver related indicators.
**Study endpoints**	**Primary efficacy endpoints：**<br>• Percent (%) change from baseline in body weight at the end of the study (W30).<br>**Secondary endpoints：**<br>**Efficacy endpoints**<br>• Proportion of subjects who achieved (yes/no) body weight reduction ≥5%, 10%, 15%, 20% from baseline.

	• The changes from baseline in body weight, waist circumference, waist to hip ratio (waist/hip circumference), and body mass index (BMI).<br>• The percentage change from baseline in body weight (%) after drug withdrawal at the last safe follow-up (W33).<br>• The changes from baseline in glucose metabolism indicators: hemoglobin A1c (HbA1c), fasting plasma glucose (FPG), fasting insulin, homeostatic model assessment for insulin resistance (HOMA-IR) and homeostasis model assessment- β (HOMA-β).<br>• The changes from baseline in cardiovascular disease risk factors: blood pressure (systolic and diastolic blood pressure), pulse, total cholesterol (TC), low-density lipoprotein cholesterol (LDL-C), high-density lipoprotein cholesterol (HDL-C), and triglycerides (TG).<br>• The changes from baseline in the impact of weight on quality of life score reported by patients: the total and individual scores of the Impact of Weight on Quality of Life–Lite Clinical Trials (IWQOL-Lite-CT), the total and individual scores of the physical and psychological parts of SF-36 scoring table.<br>**Safety endpoints：**<br>• The number of adverse events (AEs) that occurred during the study.<br>• The number of serious adverse events (SAEs) that occurred during the study.<br>• The number of adverse events of special interest (AESI) that occurred during the study: events of hypoglycaemia, adverse events of gastrointestinal (nausea, vomiting, diarrhoea, and constipation, etc.);<br>• Vital signs, Physical examination, 12-lead electrocardiogram, clinical laboratory test results (hematology, urinalysis, blood biochemistry, blood amylase, blood lipase, coagulation, calcitonin, thyroid-stimulating hormone, free tri-iodothyronine, free tetra-iodothyronine).<br>• The mental health status of the subjects: to evaluate using the Columbia-suicide severity rating scale and Patient Health Questionnaire。<br>**Immunogenic endpoints：**<br>• After administration of GZR18 injection, the changes from baseline in anti-drug antibodies (ADA) to GZR18 and neutralizing antibodies (NAb).<br>**Pharmacokinetics (PK) endpoints：**<br>• Subjects with intensive blood sampling: after the last administration, area under the plasma concentration-time curve from time 0 to the time of the last quantifiable concentration (AUC_{last}), area under the plasma concentration-time curve from time 0 to infinity ($AUC_{0\text{-}inf}$), time to peak plasma concentration (T_{max}), elimination rate constant (λz), half-life ($t_{1/2}$), the time point prior to the

	first observed/measured non-zero plasma concentration (t_{lag}), apparent clearance (CL/F), apparent volume of distribution (Vz/F) and percent of $AUC_{0\text{-}inf}$ extrapolated ($AUC_{\%extra}$), mean residence time (MRT), steady-state trough plasma concentration (C_{ss_min}), steady-state peak plasma concentration (C_{ss_max}, the same as C_{max}), average steady state plasma concentration (C_{ss_av}), degree of fluctuation (DF).<br>• All subjects: steady-state trough plasma concentration (C_{ss_min}).<br>**Exploratory efficacy endpoints：**<br>• Blood uric acid change from baseline.<br>• Fatty liver related indicators (the changes from baseline in alanine aminotransferase, aspartate aminotransferase and bile acid).
**Trial design**	This study is a multicenter, randomized, placebo-controlled phase II clinical study to evaluate the efficacy, safety, tolerability, and pharmacokinetics of GZR18 injection in Chinese adult obese/overweight subjects.<br>The obese/overweight adult subjects who are inadequately controlled with diet and exercise are included in the study.<br>Eligible subjects are randomly assigned to four different treatment groups: a group receiving subcutaneous injections of GZR18 solution once every two weeks at three different target doses (12 mg, 18 mg, 24 mg) (or corresponding volume of placebo), a group receiving subcutaneous injections of GZR18 solution once a week at a target dose of 24 mg* (or corresponding volume of placebo), and a newly added group receiving subcutaneous injections of GZR18 solution once every two weeks at a target dose of 48 mg (or corresponding volume of placebo). The specific administration methods are shown in Table 1.<br>*Note: This group is derived from the modification of the 3.0 version protocol 1-weekly 18 mg group. If subjects from the original 3.0 version protocol once a week 18 mg group do not agree to enter the once a week 24 mg dosing group, the target dose will remain at 18 mg.<br>All subjects received standard dietary and exercise guidance after randomization.
**Study period**	The maximum study period for each subject in this study is approximately 34 weeks: including a screening period of 1 week (Week -1), an administration treatment period/study visit of 30 weeks (Weeks 0 to 30, dose adjustment of weeks 10 to 18, stable administration treatment period of 12 to 20 weeks) + safety follow-up of 3 weeks (Weeks 31 to 33).

Number of subjects

This study plans to enroll 338 adult obese/overweight subjects.

- Subjects are treated with GZR18 injection/placebo once every two weeks: including three dose groups (12 mg, 18 mg, 24 mg) of GZR18 injection (or corresponding volume of placebo), with 65 subjects planned to be enrolled in each dose group (52 subjects will be treated with GZR18 injection and 13 subjects will be treated with placebo). a 48 mg once every two weeks GZR18 injection (or corresponding volume of placebo) arm has been added with a planned enrollment of 78 subjects (65 subjects will be treated with GZR18 injection and 13 subjects will be treated with placebo).

Study steps

Screening period

Each subject will undergo a screening period of up to 1 week, and those who pass the screening will be randomized to the group.

Treatment period

Eligible subjects will be randomly assigned to dose groups of 12 mg once every two weeks, 18 mg once every two weeks, 24 mg once every two weeks, and 24 mg once a week. The number of subjects in each dose group will be treated with GZR18 injection or placebo at a ratio of 4:1, and the group will be randomized double-blind. A 48 mg once every two weeks GZR18 injection (or corresponding volume of placebo) arm has been added, the number of subjects in the group will be treated with GZR18 injection or placebo at a ratio of 5:1, and the group will be randomized double-blind. The treatment period is 30 weeks, including 10 to 18 weeks of dose adjustment and 12 to 20 weeks of stable dose treatment. Administration visit (see Table 1)

Table 1 Administration design

Group	Frequency	Target Dose	Administration Design			Subjects	
			Duration (weeks)	Week of administration	Dose	GZR18 Injection	Placebo
1	once every two weeks	GZR18-12 mg	2	W0	3 mg	52	13
			4	W2/4	6 mg		
			4	W6/8	9 mg		
			20	W10/12/14/16/18/20/22/24/26/28	12 mg		
2		GZR18-18 mg	2	W0	3 mg	52	13
			4	W2/4	6 mg		
			4	W6/8	9 mg		
			4	W10/12	12 mg		
			16	W14/16/18/20/22/24/26/28	18 mg		
3		GZR18-24 mg	2	W0	3 mg	52	13
			4	W2/4	6 mg		
			4	W6/8	9 mg		
			4	W10/12	12 mg		

			4	W14/16	18 mg		
			12	W18/20/22/24/26/28	24 mg		
4		GZR18-48 mg	2	W0	3 mg	65	13
			4	W2/4	6 mg		
			4	W6/8	12 mg		
			4	W10/12	24mg		
			4	W14/16	36 mg		
			12	W18/20/22/24/26/28	48 mg		
5	once-weekly	GZR18-24 mg（18 mg）*	2	W0/1	3 mg	52	13
			4	W2/3/4/5	6 mg		
			4	W6/7/8/9	9 mg		
			4	W10/11/12/13	12 mg		
			4	W14/15/16/17	15 mg		
			12	W18/19/20/21/22/23/24/25/26/27/28/29	24 mg（18 mg）*		

*Note: If the subjects in the 18 mg once a week group of the original 3.0 version protocol do not agree to enter the 24 mg once a week group, the 18 mg dose will still be administered from W18 to W29.

- Early termination visit (ET)

Randomized subjects who withdraw from the clinical study due to meeting the withdrawal criteria need to complete ET visit as much as possible.

Safety follow-up visit period

- In the third week after the end of the study visit, the subjects undergo laboratory tests, electrocardiograms, vital signs, physical examinations, adverse event monitoring, and other examinations to complete the safety assessment.

Dose adjustment standards

The main adverse reaction of GLP-1 drugs is gastrointestinal intolerance. Subjects who do not tolerate any AE before titration up to 12 mg (≤ 12 mg) will withdraw from the trial after evaluation by the investigator.

After titrating from 12 mg to a higher dose, if intolerable, it can be retreated to the previous tolerable titration dose, with a minimum of 12 mg. Other visit and examination times remain unchanged, and the total administration time remains at 30 weeks.

If the subjects in the 48 mg dose group (once every two weeks' administration) experience intolerance when titrated from 36 mg to 48 mg. After evaluation by the investigators, they will be allowed to return to 36 mg and continue with once every two weeks' administration. Other visit and examination times will remain unchanged, and the total administration duration remain at 30 weeks.

During the experiment, investigators can also communicate with the sponsor about the dose reduction strategy initiated due to the patient's tolerance and subsequent

	dose titration strategies based on the patient's situation.<br>**During the protocol change period**<br>If subjects in the 18 mg once a week dose group of the original 3.0 version protocol are willing to enter the 24 mg once a week dose group, the study will be conducted according to the procedure of the 24 mg dose group (Group 5); if subjects are unwilling to enter the once a week 24 mg dose group, a weekly dose of 18 mg will still be administered from Week 18 to Week 29 until the end of the study. The laboratory tests at each visit time point will be consistent with the once a week 24 mg dose group.
**Biological sample collection**	**Time points of pharmacokinetic blood sampling:**<br>• Time points of sparse blood sampling:<br>✓ Dose group 1 (s.c. 12 mg once every two weeks): within 1 hour before the first administration (W0D0); within 1 hour before the first administration (W6) of the previous dose of the target dose; within 1 hour before reaching the target dose for administration (W10); within 1 hour before the 6th administration of the target dose (W20) and 72 hours ± 1 hour after administration; within 1 hour before administration at W26 and W28; blood samples are collected in the morning of the end of study visit (W30). There are a total of 8 blood sampling time points.<br>✓ Dose group 2 (s.c. 18 mg once every two weeks): within 1 hour before the first administration (W0D0); within 1 hour before the first administration (W10) of the previous dose of the target dose; within 1 hour before reaching the target dose for administration (W14); within 1 hour before the 6th administration of the target dose (W24) and 72 hours ± 1 hour after administration; within 1 hour before administration at W26 and W28; blood samples are collected in the morning of the end of study visit (W30). There are a total of 8 blood sampling time points.<br>✓ Dose group 3 (s.c. 24 mg once every two weeks): within 1 hour before the first administration (W0D0); within 1 hour before the first administration (W14) of the previous dose of the target dose; within 1 hour before reaching the target dose for administration (W18); within 1 hour before the 6th administration of the target dose (W28) and 72 hours ± 1 hour after administration; blood samples are collected in the morning of the end of study visit (W30). There are a total of 7 blood sampling time points.<br>✓ Dose group 4 (s.c. 48 mg once every two weeks): within 1 hour before the first administration (W0D0); within 1 hour before the first administration (W14) of the previous dose of the target dose; within 1 hour before reaching

	the target dose for administration (W18); within 1 hour before administration at W26; within 1 hour before the 6th administration of the target dose (W28) and 72 hours ± 1 hour after administration; blood samples are collected in the morning of the end of study visit (W30). There are a total of 7 blood sampling time points.<br>✓ Dose group 5 (s.c. 24 mg (18 mg)*once-weekly): within 1 hour before the first administration (W0D0); within 1 hour before the first administration (W14) of the previous dose of the target dose; within 1 hour before reaching the target dose for administration (W18); within 1 hour before the 6th administration of the target dose (W23) and 72 hours ± 1 hour after administration; within 1 hour before administration at W26 and W29; blood samples are collected in the morning of the end of study visit (W30). There are a total of 8 blood sampling time points.<br>*Note: If the subjects in the 18 mg once a week group of the original 3.0 version protocol do not agree to enter the 24 mg once a week group, the 18 mg dose will still be administered from W18 to W29.<br>• Time points of intensive blood sampling (at least 14 subjects receiving GZR18 injection in each group will undergo PK intensive blood sampling, specific operations can be found in the intensive blood sampling process document)<br>✓ Dose group 1 to 4 (GZR18 s.c. once every two weeks): blood samples are collected within 1 hour before the last administration at W28 (coinciding with the sparse blood sampling time point W28 before administration, no need to repeat collection), 1 hour, 6 hours, 24 hours, 48 hours, 72 hours after administration (the group receiving only 24 mg and 48 mg once every two weeks coincides with the sparse blood sampling time point W28 72 hours after administration, so there is no need to repeat the collection), 96 hours, 120 hours, 168 hours, 240 hours, 336 hours (coinciding with the sparse blood sampling time point W30 after administration, no need to repeat collection), and 504 hours (W31). The 12 mg and 18 mg once every two weeks' treatment groups have a total of 12 blood sampling time points, but two of them overlap with sparse blood sampling time points; The 24 mg and 48 mg once every two weeks' groups have a total of 12 blood sampling time points, but three of them overlap with sparse blood sampling time points.<br>✓ Dose group 5 (GZR18 s.c. once-weekly): blood samples are collected within 1 hour before the last administration at W29 (overlapping with the sparse blood sampling time point at W29 before administration, no need to repeat collection), and 1 hour, 6 hours, 24 hours, 48 hours, 72 hours, 96

	hours, 120 hours, 168 hours after administration (overlapping with the sparse blood sampling time point at W30, no need to repeat collection), 240 hours, 336 hours (W31), and 504 hours (W32). There are a total of 12 blood sampling time points, but two of them overlap with sparse blood sampling time points.<br>**Time points of immunogenic blood sampling：**<br>• Within 1 hour before the first administration (W0D0); within 1 hour before administration at W16; in the W30 visit morning of the study end/early termination; in the morning of the last safety follow-up for W33. Each group has 4 blood sampling time points.<br>**Time points of Glycated hemoglobin (HbA1c) blood sampling：**<br>During the screening period, blood samples are collected in the morning of the W30 study visit. Each group has 2 blood sampling time points.<br>**Time points of fasting blood glucose and insulin blood sampling：**<br>During the screening period, blood samples are collected in the morning of the W30 study visit. Each group has 2 blood sampling time points.<br>**Time points of safety indicators blood sampling：**<br>The safety blood sampling indicators here include blood routine, blood biochemistry, blood amylase, blood lipase, coagulation function, calcitonin, thyroid stimulating hormone, free triiodothyronine, and free thyroxine. The time point for blood pregnancy test detection is detailed in the flowchart.<br>Screening period; within 1 hour before administration of W8, W12, W16, W20, and W24; W30/ET Visit Morning; blood samples are collected in the morning of the last safety follow-up for W33. Each group has 8 blood sampling time points.
**Inclusion criteria**	Subjects must meet all of the following inclusion criteria to be eligible for the study：<br>1. Male or female aged 18–65 years (inclusive).<br>2. Obese subjects (BMI≥28 kg/m^2), or overweight subjects (24 kg/m^2≤BMI＜28 kg/m^2) with at least one of the following：<br>1） Concomitant with one or more of pre-diabetes, hypertension, dyslipidemia, fatty liver (diagnostic criteria can be found in attachments 1 and 2);<br>2） Concomitant weight-bearing joint pain；<br>3） Obesity-induced dyspnoea or obstructive sleep apnoea syndrome

	3. Able to understand the procedures and methods in this study; willing and able to maintain a stable diet and exercise lifestyle during the research period, and willing to sign the ICF voluntarily.
**Exclusion criteria**	Subjects will not be enrolled in the study if they meet any of the following criteria:：<br>1. Limb deformities or defects affecting height and body weight measurement.<br>2. Pregnant or lactating women, men or women of reproductive potential unwilling to use contraception throughout the study and for 6 months after the last dose of investigational drug.<br>3. History of drug abuse within 1 year before screening, or positive results in drug abuse screening during screening or before randomization.<br>4. Alcohol abuse history, defined as an average weekly alcohol consumption of more than 14 units for men/7 units for women (1 standard unit equivalent to 360 mL of beer or 150 mL of wine or 45 mL of spirits with 40% alcohol) within 6 months prior to screening.<br>5. Subjects who are known or suspected to be allergic to GLP-1 drugs or excipients.<br>6. Subjects with an absolute weight change greater than 5.0% due to any reason within the 3 months prior to screening. The formula for calculating weight change is: (highest weight in the 12 weeks prior to screening - lowest weight)/highest weight * 100%.<br>7. History or evidence of any of the following：<br>1) Previous diagnosis of type 1 or type 2 diabetes mellitus；<br>2) Proliferative retinopathy within 1 year prior to screening or at screening；<br>3) Previous severe hypoglycemia or recurrent symptomatic hypoglycemia (≥ 2 episodes in half a year)；<br>4) Secondary diseases or drugs leading to obesity, including: elevated cortisol (e.g. Cushing's syndrome), pituitary and hypothalamic damage leading to obesity, weight loss medicine reduction/discontinuation leading to obesity, etc；<br>5) Previous history of moderate to severe depression; or previous history of severe psychiatric disorders, such as schizophrenia, bipolar disorder, etc. Or when screening, the score of Patient Health Questionnaire (PHQ-9) ≥ 15;<br>6) Previous suicidal tendency or suicidal behaviour, or when screening, the subjects had suicidal ideation in categories 4 and 5 of the Columbia-Suicide Severity Rating Scale (C-SSRS);

	7) Systolic blood pressure ≥ 160 mmHg and/or diastolic blood pressure ≥ 100 mmHg at screening;<br>8) Previous history of thyroid C-cell carcinoma, multiple endocrine neoplasia (MEN) type 2A or 2B or related family history, or previous history of malignancy within the past 5 years (except for cured basal cell carcinoma of the skin, squamous cell carcinoma of the skin, other polyps and carcinoma in situ of the cervix)；<br>9) History of the following cardiovascular diseases: decompensated cardiac insufficiency (New York Heart Association [NYHA] Class III or IV), unstable angina or myocardial infarction, heart valve replacement surgery, coronary artery bypass grafting (CABG) or other invasive cardiovascular surgery including percutaneous coronary intervention, cerebrovascular accidents or stroke with sequelae;<br>10) History of acute and chronic pancreatitis, symptomatic gallbladder disease (excluding cholecystectomy), pancreatic injury, and other high-risk factors that may lead to pancreatitis;<br>11) Subjects with previous gastrointestinal diseases such as gastroparesis, esophageal motility disorders, gastroesophageal reflux, chronic diarrhea, fecal incontinence, and constipation, who are deemed unsuitable for participation in this study by the investigators.<br>8. Satisfaction of any laboratory test parameter with the following criteria at screening :<br>1) Serum calcitonin ⩾50 ng/L (pg/Ml);<br>2) Alanine aminotransferase (ALT) ≥ 3.0 × upper limit of normal (ULN) and/or aspartate aminotransferase (AST) ≥ 3.0 × ULN and/or total bilirubin ≥ 2.0 × ULN;<br>3) Estimated glomerular filtration rate (eGFR) < 60 mL/min/1.73 m^2, estimated by CKD-EPI equation (refer to attachment 3);<br>4) Presence of thyroid dysfunction (TSH＞6 mIU/L or ＜0.4 mIU/L);<br>5) Fasting triglycerides ⩾5.64 mmol/L (500 mg/dL);<br>6) Blood amylase or lipase＞1.5×ULN;<br>7) international normalized ratio (INR) of prothrombin time > upper limit of normal range；<br>8) Hemoglobin < 110 g/L (men) or < 100 g/L (women).<br>9) Fasting venous glucose ≥ 7.0 mmol/L or venous blood glucose after a 2-hour OGTT (75 g) ≥ 11.1 mmol/L;

	10) Subjects with positive HIV antibody, treponema pallidum antibody, hepatitis B virus surface antigen (HBsAg) and hepatitis C virus (HCV) antibody.<br>9. Presence of the following clinically significant 12-lead ECG abnormalities at screening: heart rate<50 beats/min or >100 beats/min, second- or third-degree atrioventricular (AV) block, long QT syndrome or QTc > 500 ms (The calculation formula can be found in Attachment 4), left or right bundle branch block, pre-excitation syndrome, or other significant arrhythmia (other than sinus arrhythmia).<br>10. Blood donation and/or blood loss ≥ 400 mL or bone marrow donation within 3 months prior to screening, or hematological disorders (including but not limited to haemoglobinopathy, haemolytic anaemia, thalassaemia, sickle cell anaemia).<br>11. Subjects who had undergone weight loss surgery except acupuncture and moxibustion, liposuction and abdominal liposuction within one year before screening;<br>12. Previous history of organ transplantation；or medium to major surgery, severe trauma and severe infection within 6 months prior to screening, unsuitable for participation in this study at the discretion of the investigator; or surgery scheduled during the study, except outpatient surgery posing no impact on the safety of subjects and the study results at the discretion of the investigator.<br>13. Use of any of the following drugs or treatments within 3 months prior to screening：<br>1) GLP-1 receptor (GLP-1R) agonists or GLP-1R/GCGR agonists or GIPR/GLP-1R agonists or GIPR/GLP-1R/GCGR agonists.<br>2) Drugs affecting body weight, including systemic steroids (intravenous, oral, or intra-articular administration), Antidepression such as selective serotonin reuptake inhibitors (SSRIs), serotonin noradrenaline reuptake inhibitors (SNRIs), tricyclics, tetracyclics, etc., other psychiatric agents or sedatives (e.g. imipramine, amitriptyline, mirtazapine, paroxetine, phenelzine, chlorpromazine, thioridazine, clozapine, olanzapine, valproic acid, valproic acid derivatives, lithium salts), diuretic, etc.;<br>3) Traditional Chinese medicine, Chinese patent drug, herbal medicine, health-care products, meal replacements, etc. that affect body weight.

	4) Weight loss drugs, such as sibutramine hydrochloride, orlistat, phentermine, phenylpropanolamine, mazindol, phentermine, diethylpropion, lorcaserin, phentermine/topiramate, naltrexone/bupropion, etc.;<br>5) Hypoglycemic agents, such as metformin, α-glucosidase inhibitors, sulfonylureas, DPP-4 inhibitors, SGLT-2 inhibitors, thiazolidinediones (TZDs), etc.;<br>14. Participation in clinical trials of other drugs, vaccines or medical devices within 3 months prior to screening and have received treatment.<br>Any other factor that may affect the efficacy or safety evaluation of this study and make the subject unsuitable for participation in this study at the discretion of the investigator.
**Withdrawal criteria**	1. Withdrawal at the discretion of the subject.<br>2. Subject withdrawal required by regulatory authorities.<br>3. Withdrawal at the discretion of the investigator: It refers to the situation where the enrolled subject is not suitable for further investigational drug treatment during the trial process and the investigator may request the subject to withdraw from the study.<br>1) Subjects who do not tolerate any AE before titration up to 12 mg (≤ 12 mg) will withdraw from the trial after evaluation by the investigator;<br>2) Subject with other AEs or SAEs that, in the judgment of the investigator, make continuation of investigational drug treatment inappropriate;<br>3) The subject is diagnosed with moderate to severe depression or other serious mental illness (generalized anxiety, panic attacks, bipolar disorder, schizophrenia, etc.), or the score of Patient Health Questionnaire (PHQ-9) is ≥ 15 during the trial;<br>4) Subjects exhibit suicidal tendencies or behaviors, or exhibit suicidal ideation in categories 4 and 5 of the Columbia-Suicide Severity Rating Scale during the trial;<br>5) Pregnancy, planned pregnancy or unwillingness to take effective contraceptive measures;<br>6) Subjects receiving treatment with GLP-1 receptor agonists and DPP-4 inhibitors;<br>7) Patients with acute/chronic pancreatitis or high-risk factors of pancreatitis, such as symptomatic gallbladder disease (such as multiple gallstones) and pancreatic injury;

	8) Suspected or confirmed thyroid C-cell carcinoma, multiple endocrine neoplasia (MEN) 2A or 2B, or other malignant tumors;<br>9) New or persistently progressive thyroid-stimulating hormone (TSH > 10 mIU/L) or calcitonin abnormality (calcitonin > 50 pg/mL) that, in the judgment of the investigator, makes continuation of investigational drug treatment inappropriate;<br>10) Subjects occurring a severe hypoglycemic event (a hypoglycemic event requiring assistance to obtain carbohydrates, glucagon, or other rescue measures) or a recurrent hypoglycemic event (2 or more episodes in 1 month) without an obvious precipitating factor;<br>11) Subjects with poor compliance, which affects safety and tolerability determinations; including but not limited to:<br>• Failure to take drugs and undergo tests as required;<br>• Use of other drugs or foods that interfere with the safety evaluation;<br>• Rejection by the subject for the dose level or escalation mode to which he/she is assigned to be received;<br>• Engagement in other behaviors that affect study results.
**Study termination criteria**	Study termination means the termination of all trials before the clinical trial is ended as per the protocol. The purpose of trial termination is mainly to protect the rights and interests of subjects, ensure the quality of the trial and avoid unnecessary economic losses.<br>1. In case of any serious safety issues during the trial, the investigator shall terminate the trial in a timely manner if he/she deems it necessary to terminate the trial;<br>2. If the drug is found to have no clinical value during the trial, the trial shall be terminated timely to protect the rights and interests of the subjects and avoid unnecessary economic losses;<br>3. If major errors in the clinical trial protocol are found in the trial, making it difficult to evaluate the PK/PD profiles and safety of the drug; or significant deviation is made during the implementation of the protocol, which affects the final evaluation of the drug, the trial should be terminated;<br>4. The sponsor requests termination on the premise of fully safeguarding the safety and rights of the subjects (e.g., funding reasons, management reasons, etc.);<br>5. The regulatory authority or ethics committee orders the trial to be terminated for some reasons.

Investigational drug

	Investigational drug	Placebo
Name	GZR18 Injection	Placebo (The vehicle of GZR18, which shares same components with GZR18 injection but contains no GZR18. Placebo is similar with the investigational drug in appearance, weight and smell, indistinguishable)
Strength	Please refer to the Certificate of analysis (COA) and injection SOP for details	0 mg/mL
Manufacturer	Gan & Lee Pharmaceuticals Co., Ltd	Gan & Lee Pharmaceuticals Co., Ltd
Administration method	Subcutaneous injection	Subcutaneous injection
Storage conditions	protect from light, 2°C-8°C	protect from light, 2°C-8°C

Note: The above drugs must be balanced to room temperature before use. Production batch number can be found in the COA

Concomitant therapy

Any treatment other than the investigational drug, including over-the-counter drugs, must be accurately documented in the source documents and Electronic case report form (eCRF).

All concomitant medication taken within the three months prior to screening shall be recorded in the eCRF at the screening visit, and thereafter, records shall only be made if there is a change in medication. The corresponding date when any changed administration is initiated shall be recorded in the eCRF.

For all subjects, all changes to their concomitant medications shall be recorded on the pages for concomitant medication of the eCRF, respectively. Such recording shall cover the drug name (generic name, if available, as well as the trade name), indication(s), dosage form, specification, date of administration, usage and dosage.

The above description also applies to non-drug therapy.

Prior and concomitant medications are listed by subject using WHODrug (current effective version) coding and summarized by the Anatomical Therapeutic Chemical (ATC) classification system.

Prohibited concomitant treatment (drug and non-drug therapies) during the trial

1) GLP-1 receptor (GLP-1R) agonists, GLP-1R/Glucagon Receptor (GCGR) agonists, Glucose-dependent insulinotropic polypeptide receptor (GIPR)/GLP-1R agonists or GIPR/GLP-1R/GCGR agonists;

2) Drugs affecting body weight, including systemic steroids (intravenous, oral, or intra-articular administration), Antidepression such as selective serotonin reuptake inhibitors (SSRIs), serotonin noradrenaline reuptake inhibitors (SNRIs), tricyclics, tetracyclics, etc., other psychiatric agents or sedatives (e.g. imipramine, amitriptyline, mirtazapine, paroxetine, phenelzine, chlorpromazine, thioridazine, clozapine, olanzapine, valproic acid, valproic acid derivatives, lithium salts), diuretic, etc.;

3) Traditional Chinese medicine, Chinese patent drug, herbal medicine, health-care products, meal replacements, etc. that affect body weight;

	4) Weight loss drugs, such as sibutramine hydrochloride, orlistat, phentermine, phenylpropanolamine, mazindol, phentermine, diethylpropion, lorcaserin, phentermine/topiramate, naltrexone/bupropion, etc.;<br>5) Hypoglycemic agents, such as metformin, α-glucosidase inhibitors, sulfonylureas, DPP-4 inhibitors, SGLT-2 inhibitors, thiazolidinediones (TZDs), insulin, insulin analogs, etc.;<br>**Allowable concomitant treatment (drug and non-drug therapies) during the trial**<br>If the investigator judges it necessary to use non-prohibited concomitant treatment (drug and non-drug therapy), the investigator shall truthfully record such information as name, duration, dosage, and the response to treatment, etc.
**Randomization method**	The random statistician will generate the project random table. The "block randomization" method is used to generate random numbers using the PLAN process of statistical software SAS 9.4 version or above, the subjects are randomly divided into GZR18 s.c. 12 mg once every two weeks group, GZR18 s.c. 18 mg once every two weeks group, GZR18 s.c. 24 mg once every two weeks group, GZR18 s.c. 24 mg once-weekly group (the original GZR s.c. 18 mg once-weekly group), After that, subjects in each GZR18 dose group are randomly divided into the study group and the control group (placebo) according to the ratio of 4:1. The random table is reproducible, and the seed parameters of the initial value of the set random number need to be saved. According to the version 4.0 protocol, a GZR18 s.c. 48 mg once every two weeks group is added, and 78 subjects are randomly divided into the study group and the control group (placebo) according to the ratio of 5:1. The random table of the new dose group is also reproducible, and the seed parameters of the initial value of the set random number need to be saved.
**Statistic analysis**	**Sample size calculation**<br>Based on the study results of similar drugs and the non-clinical data of GZR18 and the existing clinical human pharmacodynamic data of GZR18, it is estimated that the difference of the percentage of body weight change from baseline at W30 between GZR 18 and placebo group is 11% (GZR18 weight loss of 13%, placebo group weight loss of 2%), the combined standard deviation is 10%, the power of test is 0.8, unilateral α is 0.025, GZR18 group: placebo group: 4:1, then the calculated sample size is 40

subjects for GZR18 investigational product and 10 subjects for placebo. Considering the 20% dropout rate, 52 subjects receive GZR18 investigational drug and 13 subjects receive placebo actually. There are five dose groups of GZR18, including the 12 mg/18 mg/24 mg once every two weeks' group and the 24 mg (18 mg) once-weekly group, with 52 subjects in each investigational drug group and 13 subjects in the placebo group; the subjects in the 48 mg once every two weeks' group may not titrate 48 mg due to intolerance to AE, so the number of subjects in the GZR18 group is increased to 78 (65 subjects for the GZR18 investigational drug and 13 people for the placebo group). The total sample size is 338 subjects.

Analysis sets

Full Analysis Set (FAS): FAS includes all randomized subjects who received at least one dose of the investigational drug. The data set is the main analysis set of demographic data, baseline indicators, medical history and curative effect.

Per Protocol Set (PPS): PPS is a subset of FAS, which includes subjects who met all inclusion criteria, did not meet exclusion criteria, had primary endpoint efficacy indicators and did not have other major protocol deviations. The data set is used for the supportive analysis of efficacy.

Safety Set (SS): SS includes all randomized subjects who received at least one dose of investigational drug and had at least one safety evaluation. This data set is used for statistical analysis of safety indicators.

PK Concentration Set (PKCS): PKCS includes all randomized subjects who received at least one dose of investigational drug and had at least one measured plasma concentration of the investigational drug available after medication.

PK Parameter Set (PKPS): PKPS includes all randomized subjects who received at least one dose of the investigational drug and had at least one valid PK parameter of the investigational drug during the study.

Descriptives

The continuity data will be described by non-missing observations, mean, median, standard deviation, minimum and maximum. The classification data will be described by frequency and percentage. For the calculation of percentage, the denominator will be the population of relevant subjects (unless otherwise specified).

Baseline is defined as the last non-missing measurement before the first use of investigational drug (unless otherwise specified).

Efficacy evaluation

hypothesis-testing：

	Percentage change in body weight from baseline to W30 (one-sided α=0.025)
Null hypothesis	H_0: $\mu_T - \mu_C \geqslant 0$

Alternative hypothesis	H_1: $\mu_T - \mu_C < 0$

μ_T is the mean percentage change of body weight from baseline in the first to fifth dose groups after 30 weeks of GZR18 treatment (μ_T includes μ_{T1}, μ_{T2}, μ_{T3}, μ_{T4} and μ_{T5}, which corresponds to GZR18 s.c. 12 mg once every two weeks, GZR18 s.c. 18 mg once every two weeks, GZR18 s.c. 24 mg once every two weeks, GZR18 s.c. 48 mg once every two weeks and GZR18 s.c. 24 mg once-weekly respectively), μ_C is the mean percentage change of body weight from baseline in the first to fifth dose groups after 30 weeks of placebo treatment. α is significance level (Type I error rate).

Primary endpoint analysis: the percentage of body weight change (%) from baseline at week 30 will be taken as the primary endpoint, which will be analyzed based on the covariance model (ANCOVA), where, with baseline body weight as the covariate and group as the fixed effect, and the change from baseline is the dependent variable. The following indicators will be then calculated: the least-squares mean (LSMean) and its two-sided 95% CI of the percentage of the change from baseline in both groups, as well as the LSMean difference between the treatment and control groups and its two-sided 95% CI.

Secondary endpoint analysis: The continuity variables will be analyzed by the same method as the primary endpoint analysis. The number of cases reaching the target end point and the rate of reaching the target and its two-sided 95% CI will be counted in the analysis of the proportion between the two groups. At the same time, the rate difference between the two groups (the investigational drug group and the placebo group) and its two-sided 95% CI will be calculated. The rate of reaching the target and the two-sided 95% CI of the rate difference between the two groups will be calculated by the Clopper-Pearson Exact method.

Sensitivity analysis

Sensitivity analysis of main efficacy indicators.

1. The Mixed Model Repeated Measures (MMRM) method will be used to incorporate all time points from baseline to week 30 into the Linear Mixed model (LMM) as a supporting analysis to compare the efficacy differences between the treatment group and the placebo group.
2. Consistent with the primary endpoint analysis method, some specific populations will be excluded for comparison of efficacy between groups.

Statistical analysis of safety endpoints

Adverse events will be summarized for each treatment group using Medical Dictionary for Regulatory Activities (MedDRA) according to System Organ Class (SOC) and Preferred term (PT). All adverse events are presented in the form of a list,

	which at least includes the start date, end date, severity, relationship with the investigational drug, outcome, duration and other information.<br>Safety variables (vital signs, laboratory data, ECG, etc.) will be grouped and summarized according to the time points specified in the protocol. Concomitant medications will be classified and summarized by group. All other safety data (physical examination, blood pregnancy, PHQ-9 score, etc.) will be reflected in the form of a list. The changes of laboratory examination results, ECG and physical examination before and after treatment will be statistically described in the form of a cross table, and the list of subjects with abnormal clinical significance after treatment will be listed.<br>The summarized table will present the number and percentage of events. In addition, the Treatment Emergent Adverse Events (TEAEs) will be summarized according to the severity and the relationship with the investigational drug. TEAE is defined as adverse events after the use of investigational drugs, and the analysis of adverse events will be based on TEAE. Serious adverse events will also be summarized and listed separately.<br>For the same subject, if an adverse event occurs multiple times, it will be counted as one case when calculating the incidence of adverse events.<br>The adverse events leading to the early termination of the study medication will be described.<br>Immunogenicity evaluation index: the titer of ADA and NAB in serum according to groups, and the number and incidence of subjects with positive ADA and NAB will be described.<br>Heart rate, blood amylase and lipase will also be further analyzed using the covariance model (ANCOVA).<br>The dose-dependent assessment of safety and tolerability is based on five GZR18 injection study groups with four dose groups and two administration methods, and compared with the placebo control group to summarize the adverse events in each group.<br>**PK analysis**<br>Based on PKPs, Phoenix WinNonlin 8.3 or above is used for PK analysis. The list and descriptive statistics of the investigational drugs are carried out, and the average drug concentration-time curves of individual and group are drawn respectively. The PK parameters are calculated by non atrioventricular model according to the actual blood sampling time, and the parameters are summarized by descriptive statistics. The main pharmacokinetic parameters are summarized by the number of subjects, arithmetic mean, standard deviation, coefficient of variation (CV),

	median, minimum, maximum, geometric mean and geometric coefficient of variation. Power model is used to explore the linear characteristics of pharmacokinetic parameters and dose when the data are sufficient.<br>**Statistical programming**<br>Statistical programming will be completed with SAS 9.4 version or above, Phoenix WinNonlin 8.3 version or above.

1.2 Study flowchart

Assessment	Screening	Basline	Study visit														Safety follow-up	Early termination
**Visit**	**V1**	**V2**	**V3**	**V4**	**V5**	**V6**	**V7**	**V8**	**V9**	**V10**	**V11**	**V12**	**V13**	**V14**	**V15**	**V16**	**V17**	**V18**	**ET**
**Week(W)**	**W-1**	**W0**	**W2**	**W4**	**W6**	**W8**	**W10**	**W12**	**W14**	**W16**	**W18**	**W20**	**W22**	**W24**	**W26**	**W28**	**W30**	**W33**	
**Day(D)**	**D-7~D-1**	**D0**	**D14**	**D28**	**D42**	**D56**	**D70**	**D84**	**D98**	**D112**	**D126**	**D140**	**D154**	**D168**	**D182**	**D196**	**D210**	**D231**	
**Time window(days)**			**±2**	**±2**	**±2**	**±2**	**±2**	**±2**	**±2**	**±2**	**±2**	**±2**	**±2**	**±2**	**±2**	**±2**	**±2**	**±3**	
Signing of ICF	X																		
Inclusion and exclusion criteria	X	X																	
Demographics[1]	X																		
Past history and comorbidities[2]	X																		
Concomitant treatment/medication	X	X	X	X	X	X	X	X	X	X	X	X	X	X	X	X	X	X	X
Immunoserologic panel test[3]	X																		
Pregnancy test [4]	X	X		X		X		X		X		X		X			X	X	X
Chest X-ray (anteroposterior and lateral view) or lung CT[5]	X																		
Hepatobiliary, pancreatic, splenic and urinary system color Doppler ultrasound[5]	X																		
OGTT	X																		

**Assessment**	**Screening**	**Basline**	**Study visit**															**Safety follow-up**	**Early termination**
**Visit**	**V1**	**V2**	**V3**	**V4**	**V5**	**V6**	**V7**	**V8**	**V9**	**V10**	**V11**	**V12**	**V13**	**V14**	**V15**	**V16**	**V17**	**V18**	**ET**
**Week(W)**	**W-1**	**W0**	**W2**	**W4**	**W6**	**W8**	**W10**	**W12**	**W14**	**W16**	**W18**	**W20**	**W22**	**W24**	**W26**	**W28**	**W30**	**W33**	
**Day(D)**	**D-7~D-1**	**D0**	**D14**	**D28**	**D42**	**D56**	**D70**	**D84**	**D98**	**D112**	**D126**	**D140**	**D154**	**D168**	**D182**	**D196**	**D210**	**D231**	
**Time window(days)**			**±2**	**±2**	**±2**	**±2**	**±2**	**±2**	**±2**	**±2**	**±2**	**±2**	**±2**	**±2**	**±2**	**±2**	**±2**	**±3**	
Drug abuse screening (urine)[6]	X	X																	
Blood biochemistry (including blood lipids), hematology, and coagulation[7]	X					X		X		X		X		X			X	X	X
Amylase and lipase	X					X		X		X		X		X			X	X	X
TSH/FT3/FT4	X					X		X		X		X		X			X	X	X
Calcitonin	X					X		X		X		X		X			X	X	X
Urinalysis[7]	X					X		X		X		X		X			X	X	X
ECG[8]	X					X		X		X		X		X			X	X	X
Physical examinations[9]	X					X		X		X		X		X			X	X	X
Vital signs[10]	X	X	X	X	X	X	X	X	X	X	X	X	X	X	X	X	X	X	X
Fundus examination	X									X							X	X	X
Injection site observation[11]		X	X	X	X	X	X	X	X	X	X	X	X	X	X	X	X	X	X
Immunogenicity blood sampling[12]		X		nab						X							X	X	X
PHQ-9 scores	X							X						X				X	X
Evaluation of Columbia-suicide severity rating scale	X							X						X				X	X
Adverse event		X	X	X	X	X	X	X	X	X	X	X	X	X	X	X	X	X	X

**Assessment**	**Screening**	**Basline**	**Study visit**															**Safety follow-up**	**Early termination**
**Visit**	**V1**	**V2**	**V3**	**V4**	**V5**	**V6**	**V7**	**V8**	**V9**	**V10**	**V11**	**V12**	**V13**	**V14**	**V15**	**V16**	**V17**	**V18**	**ET**
**Week(W)**	**W-1**	**W0**	**W2**	**W4**	**W6**	**W8**	**W10**	**W12**	**W14**	**W16**	**W18**	**W20**	**W22**	**W24**	**W26**	**W28**	**W30**	**W33**	
**Day(D)**	**D-7~D-1**	**D0**	**D14**	**D28**	**D42**	**D56**	**D70**	**D84**	**D98**	**D112**	**D126**	**D140**	**D154**	**D168**	**D182**	**D196**	**D210**	**D231**	
**Time window(days)**			**±2**	**±2**	**±2**	**±2**	**±2**	**±2**	**±2**	**±2**	**±2**	**±2**	**±2**	**±2**	**±2**	**±2**	**±2**	**±3**	
Body measurements[13]	X	X	X	X	X	X	X	X	X	X	X	X	X	X	X	X	X	X	X
Fasting blood glucose、insulin[14]	X																X		
Glycosylated hemoglobin	X																X		
IWQoL-Lite-CT questionnaire		X						X						X			X		X
SF-36 questionnaire		X						X						X			X		X
PK blood sampling[15]		X			X[a]		X[b]		X[c]		X[d]	X[e]		X[f]	X	X[g]	X		
Randomization		X																	
Hospital Administration[16]		X	X	X	X	X	X	X	X	X	X	X	X	X	X	X			
Training and distribution of injection pen[17]		X																	
Dispensing of drugs (only once-weekly admistration)[18]		X	X	X	X	X	X	X	X	X	X	X	X	X	X				
Recovery of drugs (only once-weekly admistration)[18]			X	X	X	X	X	X	X	X	X	X	X	X	X	X			
Evaluation of drug use compliance outside hospital (only			X	X	X	X	X	X	X	X	X	X	X	X	X	X			

**Assessment**	**Screening**	**Basline**	**Study visit**															**Safety follow -up**	**Early termin ation**
**Visit**	**V1**	**V2**	**V3**	**V4**	**V5**	**V6**	**V7**	**V8**	**V9**	**V10**	**V11**	**V12**	**V13**	**V14**	**V15**	**V16**	**V17**	**V18**	**ET**
**Week(W)**	**W-1**	**W0**	**W2**	**W4**	**W6**	**W8**	**W10**	**W12**	**W14**	**W16**	**W18**	**W20**	**W22**	**W24**	**W26**	**W28**	**W30**	**W33**	
**Day(D)**	**D-7~D-1**	**D0**	**D14**	**D28**	**D42**	**D56**	**D70**	**D84**	**D98**	**D112**	**D126**	**D140**	**D154**	**D168**	**D182**	**D196**	**D210**	**D231**	
**Time window(days)**			**±2**	**±2**	**±2**	**±2**	**±2**	**±2**	**±2**	**±2**	**±2**	**±2**	**±2**	**±2**	**±2**	**±2**	**±2**	**±3**	
once-weekly admistration)[18]																			
Diet/exercise instruction		X	X	X	X	X	X	X	X	X	X	X	X	X	X	X	X		
Diary card recording training		X																	
Distribution of diary cards		X	X	X	X	X	X	X	X	X	X	X	X	X	X	X	X		
Recovery of diary cards			X	X	X	X	X	X	X	X	X	X	X	X	X	X	X	X	X

Note： ET：early termination.

1. Demographic data: including date of birth, age, sex and ethnicity.
2. Past history/comorbidities: including past medical history/concomitant diseases, prior treatment history, allergy history, personal history (smoking history, alcohol consumption history), family history, etc.
3. Immunoserologic panel test: infection screening tests for hepatitis B surface antigen (HBsAg), hepatitis C virus antibody (HCV-Ab), human immunodeficiency virus antibody (HIV-Ab), and Treponema pallidum antibody.
4. Pregnancy test: only women of childbearing age are tested. See Annex 6 for the definition of women of childbearing age; during the screening period, W12, W24, W30, W33 and early withdrawal, blood pregnancy examination will be performed, and urine pregnancy examination will be performed at other visits. If the urine pregnancy examination result is positive or suspected to be positive, or the subject is not convenient for urine pregnancy during menstruation or the research center is unable to perform urine pregnancy examination, blood pregnancy examination will be performed.
5. For chest X-ray (anteroposterior and lateral view) or lung CT and hepatobiliary, pancreatic, splenic and urinary system color Doppler ultrasound, result within 3 months prior to screening are acceptable.
6. Drug abuse screening (urine) refers to the urine test of amphetamines, benzodiazepines, marijuana, cocaine, methadone and morphine-like drugs with the test kits.

7. Hematology: including white blood cell count, lymphocyte count, monocyte count, neutrophil count, eosinophil count, basophil count, lymphocyte percentage, monocyte percentage, neutrophil percentage, eosinophil percentage, basophil percentage, red blood cell count, hemoglobin, and platelet count. Blood biochemistry: including alanine aminotransferase (ALT), aspartate aminotransferase (AST), γ-glutamyl transpeptidase (GGT), alkaline phosphatase (ALP), total protein (TP), albumin (ALB), total bilirubin (TBIL), direct bilirubin (DBIL), blood urea nitrogen (BUN) or urea, creatinine (Cr), triglycerides (TG), total cholesterol (TC), low density lipoprotein cholesterol (LDL-c), and high density lipoprotein cholesterol (HDL-c). Coagulation: prothrombin time (PT), thrombin time (TT), fibrinogen (FIB), activated partial thromboplastin time (APTT), and international normalized ratio (INR), of which INR is a mandatory item, and other coagulation test items may be selected according to the conditions of the site. Midstream urine should be collected for urinalysis, which at least includes urobilinogen, bilirubin, ketone body, protein, nitrite, glucose, pH, red blood cell count, and white blood cell count in urine.
8. 12-lead ECG: heart rate, PR interval, QRS interval, QT interval, and QTc interval, examined in a supine position.
9. Physical examinations: Including examinations of general conditions, skin, neck (including thyroid), head, chest, abdomen, back, lymph nodes, extremities, and nervous system.
10. Vital signs: Including body temperature, blood pressure, respiratory rate, pulse and heart rate. On dosing days, vital signs will be examined before dosing.
11. Injection site observation: Injection site observation are observed 30 min before and after dosing as judged by physical examination by the investigator.
12. Immunogenicity: Including anti-drug antibody (ADA) and neutralizing antibody (NAb), sampling point: within 1 h before the first administration (W0D0); W16 within 1 h before administration; the morning of W30 study completion/early termination (ET) visit; W33 last safety follow-up morning.
13. Body measurements: For the same subject, weight and waist circumference should be measured under as consistent circumstances as possible (in a fasting state with clothes of similar thickness) each time. Body height is measured only during the screening period to calculate BMI.
14. Fasting blood glucose, fasting insulin, glycosylated hemoglobin: screening period, blood samples are collected in the morning after the W30 study.
15. Time points of pharmacokinetic blood sampling：
    - Time points of sparse blood sampling：
    - ✓ Dose group 1 (s.c. 12 mg once every two weeks): Within 1 hour before the first administration (W0D0); within 1 hour before the first administration (W6) of the previous dose of the target dose; within 1 hour before reaching the target dose for administration (W10); within 1 hour before the 6th administration of the target dose (W20) and 72 hours ± 1 hour after administration; within 1 hour before administration at W26 and W28; blood samples are collected in the morning of the end of study visit (W30). There are a total of 8 blood sampling time points.
    - ✓ Dose group 2 (s.c. 18 mg once every two weeks): Within 1 hour before the first administration (W0D0); within 1 hour before the first administration (W10) of the previous dose of the target dose; within 1 hour before reaching the target dose for administration (W14); within 1 hour before the 6th administration of the target dose (W24) and 72 hours ± 1 hour after administration; within 1 hour before administration at W26 and W28; blood samples are collected in the

morning of the end of study visit (W30). There are a total of 8 blood sampling time points.

- ✓ Dose group 3 (s.c. 24 mg once every two weeks): Within 1 hour before the first administration (W0D0); within 1 hour before the first administration (W14) of the previous dose of the target dose; within 1 hour before reaching the target dose for administration (W18); within 1 hour before the 6th administration of the target dose (W28) and 72 hours ± 1 hour after administration; blood samples are collected in the morning of the end of study visit (W30). There are a total of 7 blood sampling time points
- ✓ Dose group 4 (s.c. 48 mg once every two weeks): Within 1 hour before the first administration (W0D0); within 1 hour of the first administration (W14) of the previous dose of the target dose; Within 1 hour before reaching the target dose for administration (W18); within 1 hour before administration at W26; within 1 hour before the 6th administration of the target dose (W28) and 72 hours ± 1 hour after administration; blood samples are collected in the morning of the end of study visit (W30). There are a total of 7 blood sampling time points.
- ✓ Dose group 5 (s.c. 24 mg (18 mg)*once-weekly): Within 1 hour before the first administration (W0D0); Within 1 hour before the first administration (W14) of the previous dose of the target dose; Within 1 hour before reaching the target dose for administration (W18); Within 1 hour before the 6th administration of the target dose (W23) and 72 hours ± 1 hour after administration; Within 1 hour before administration at W26 and W29; Blood samples are collected in the morning of the end of study visit (W30). There are a total of 8 blood sampling time points.

- Time points of intensive blood sampling (not shown in the flowchart): Dose group 1 to 4 (GZR18 s.c. once every two weeks): Blood samples are collected within 1 hour before the last administration at W28 (coinciding with the sparse blood sampling time point W28 before administration, no need to repeat collection), 1 hour, 6 hours, 24 hours, 48 hours, 72 hours after administration (the group receiving only 24 mg and 48 mg once every two weeks coincides with the sparse blood sampling time point at W28 72 hours after administration, so there is no need to repeat the collection), 96 hours, 120 hours, 168 hours, 240 hours, 336 hours (coinciding with the sparse blood sampling time point at W30 after administration, no need to repeat collection), and 504 hours (W31). Dose group 5 (GZR18 s.c. once-weekly) Blood samples are collected within 1 hour before the last administration at W29 (overlapping with the sparse blood sampling time point at W29 before administration, no need to repeat collection), and 1 hour, 6 hours, 24 hours, 48 hours, 72 hours, 96 hours, 120 hours, 168 hours after administration (overlapping with the sparse blood sampling time point at W30, no need to repeat collection), 240 hours, 336 hours (W31), and 504 hours (W32).

16. In Hospital Administration (complete at 9:00 ± 3 hours on the day of the visit): after screening, each subject in the dose group 1 to dose group 4 is injected once every two weeks, each time in the hospital, and placebo is injected with the same volume; After screening, each subject in dose group 5 will be injected once a week, and then come to the hospital for injection once two weeks. The injection will be carried out alternately inside and outside the hospital. If the subject could not inject by himself or the drug could not be stored outside the hospital, the investigator will inject the drug in the hospital, and the placebo will be given the same volume of drug. **Subjects in GZR18 s.c. 24 mg (18 mg)*dose group will be injected with the investigational drug in the hospital at W23 and W29,**

and PK blood sampling will be conducted (not shown in the flow chart).

17. Training on the use of injection pen: see the attached instructions or other SOPs and other documents for the specific use methods and administration methods of injection pen. Only the subjects who need out of hospital injection are given the injection pen.
18. Only the dose group 5 (s.c. once-weekly) is applicable. After the subjects are injected with drugs during each visit, the study drug for the next injection will be distributed to them for out of hospital injection next week.
19. The time window of drug administration outside the hospital for subjects who are administered once a week is the same as visiting the hospital, which is ± 2 days.
    a. W6: Only sampling within 1 hour before administration from the group of s.c. 12 mg once every two weeks
    b. W10: Only sampling within 1 hour before administration from the group of s.c. 12 mg and 18 mg once every two weeks
    c. W14: Only sampling within 1 hour before administration from the group of s.c. 18 mg, 24 mg and 48 mg once every two weeks
    d. W18: Only sampling within 1 hour before administration from the group of s.c. 24 mg and 48 mg once every two weeks, Only sampling within 1 hour before administration from the group of s.c. 24 mg (18 mg)* once-weekly.
    e. W20: Only sampling within 1 hour before administration and 72 hours after administration from the group of s.c. 12 mg once every two weeks.
    f. W24: Only sampling within 1 hour before administration and 72 hours after administration from the group of s.c. 18 mg once every two weeks.
    g. W28: Only sampling within 1 hour before administration from the group of s.c. 12 mg, 18 mg, 24 mg and 48 mg once every two weeks, as well as sampling 72 hours after administration from the group of s.c. 24 mg and 48 mg once every two weeks.

The following dose group are not shown in the flowchart: for the group of s.c. 24 mg (18 mg) once a week *, W23: sampling within 1 hour before administration and 72 hours after administration, W29: sampling within 1 hour before administration

*Note: If subjects from the original 3.0 version protocol 1-weekly 18 mg group do not agree to enter the 1-weekly 24 mg dosing group, the target dose will remain at 18 mg.

1.3 PK blood sampling schedule

Table 1-1 PK blood sampling schedule-once every two weeks (dose group 1 to 4) (based on PK blood sampling time window)

Serial Number	Visits	Week	Collection time	Time window	Note
1	V2	W0	D0	Not Applicable	Within 1 hour before administration
2	V5	W6	D42	±2 d	Only within 1 hour before administration of dose group 1
3	V7	W10	D70	±2 d	Only within 1 hour before administration in dose groups 1 and 2；
4	V9	W14	D98	±2 d	Only within 1 hour before administration in dose groups 2, 3, and 4；
5	V11	W18	D126	±2 d	Only within 1 hour before administration in dose groups 3 and 4
6	V12	W20	D140	±2 d	Only within 1 hour before administration and 72 hours ± 1 hour after administration in dose group 1
7	V14	W24	D168	±2 d	Only within 1 hour before administration and 72 hours ± 1 hour after administration in dose group 2
8	V15	W26	D182	±2 d	Within 1 hour before administration
9	V16	W28	D196	±2 d	Only within 1 hour before administration in dose groups 1, 2, 3, and 4, and 72 hours ± 1 hour after administration in dose groups 3 and 4
10		After the last administration	1 h	±5 min	
11		After the last administration	6 h	±15 min	
12		After the last administration	24 h	±1 h	
13		After the last administration	48 h	±1 h	
14		After the last	72 h	±1 h	The dose group 3 and 4 overlap with sparse blood sampling at 72 hours after

		administration			W28 administration, and there is no need to repeat the collection
15		After the last administration	96 h	±2 h	
16		After the last administration	120 h	±2 h	
17		After the last administration	168 h	±2 h	
18		After the last administration	240 h	±1 d	
19	V17	After the last administration (Intensive blood sampling) W30 (Sparse blood sampling)	336 h (Intensive blood sampling) D210 (Sparse blood sampling)	±1 d (Intensive blood sampling) ±2 d (Sparse blood sampling)	
20		After the last administration	504 h	±1 d	W31

Note: dose group 1: s.c. 12 mg once every two weeks; dose group 2: s.c. 18 mg once every two weeks; dose group 3: s.c. 24 mg once every two weeks; dose group 4: s.c. 48 mg once every two weeks.

Table 1-2 PK blood sampling schedule-once-weekly (dose group 5) (based on PK blood sampling time window)

Serial Number	Visits	Week	Collection time	Time window	Note
1	V2	W0	D0	Not Applicable	Within 1 hour before administration
2	V9	W14	D98	±2 d	Within 1 hour before administration
3	V11	W18	D126	±2 d	Within 1 hour before administration
4		W23	D161	±2 d	Within 1 hour before administration and 72 hours ± 1 hour after administration
5	V15	W26	D182	±2 d	Within 1 hour before administration
6		W29	D203	±2 d	Within 1 hour before administration
7		After the last administration	1 h	±5 min	
8		After the last administration	6 h	±15 min	
9		After the last administration	24 h	±1 h	
10		After the last administration	48 h	±1 h	
11		After the last administration	72 h	±1 h	
12		After the last administration	96 h	±2 h	
13		After the last administration	120 h	±2 h	
14	V17	After the last administration (Intensive blood sampling)<br>W30 (Sparse blood sampling)	168 h (Intensive blood sampling)<br>D210 (Sparse blood sampling)	±2 h (Intensive blood sampling)<br>±2 d (Sparse blood sampling)	
15		After the last administration	240 h	±1 d	
16		After the last administration	336 h	±1 d	W31
17		After the last administration	504 h	±1 d	W32

Note: dose group 5: s.c. 24 mg (18 mg) *once-weekly. *Note: If the subjects in the 18 mg once a week group of the original 3.0 version protocol do not agree to enter the 24 mg once a week group, the 18 mg dose will still be administered from W18 to W29.

1.4 Study Design Chart

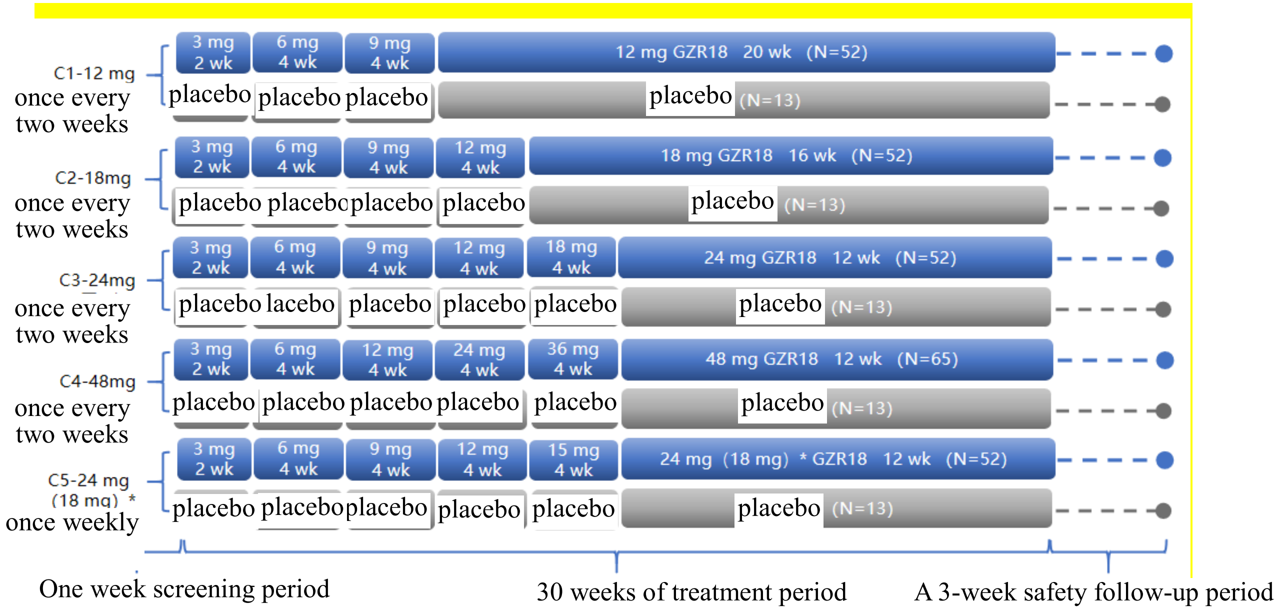

*If the subjects in the 18 mg once a week group of the original 3.0 version protocol do not agree to enter the 24 mg once a week group, the target dose will still be 18 mg.

2. Introduction

2.1 Background and epidemiology of the target disease

Obesity is a state of the body where excessive accumulation of fat leads to health damage. In the past two decades, obesity has become a serious global medical and social problem. In a survey involving over 192 million people, investigators analyzed global data trends from 1975 to 2014 and predicted that by 2025, the global trend of obesity would be 18% for males and over 21% for females; Among them, 6% of males and 9% of females have a body mass index (BMI) exceeding 40 kg/m^2, respectively. In China, surveys showed that 266 million adults are overweight and 53 million are obese, accounting for 27.8% and 5.5% of the total adult population, respectively [1].

In 2017, obesity was classified by the World Obesity Federation as a chronic recurrent disease and is believed to be the result of the interaction of genetic, metabolic, environmental, and behavioral factors. Obesity can also increase the incidence and mortality rates. Obese patients are more likely to suffer from coronary heart disease, hypertension, hyperlipidemia, diabetes, cerebrovascular accident, osteoarthritis, sleep apnea and other diseases than normal people. Some surveys also suggest that obesity is associated with a higher incidence of certain cancers. Compared to normal weight individuals of the same age, obese patients have an increased risk of death compared to deaths from various causes.

Traditionally, lifestyle interventions such as diet, behavior, and exercise counseling have been the main treatment methods for obesity. However, the causes of obesity are multifactorial and are related to many complex environmental, physiological, and genetic factors, which also make the treatment of obesity challenging. Scientific evidence suggests that weight gain leads to hormonal, metabolic, and neurochemical adaptations, which may affect the regulation of energy balance, promote the maintenance of weight gain, and make weight loss difficult. In addition, during weight loss, the body appears to compensate by reducing metabolism and increasing the production of appetite stimulating hormones. These combined effects often benefit weight recovery and explain why sustained weight loss is difficult to achieve, as well as why many people strive to maintain their weight through simple lifestyle interventions.

Surgical treatment provides an effective alternative for some severely obese patients. However, these methods are not available or appropriate for many obese patients, and are often associated with the risks and complications associated with inherent surgical issues

in obese patients.

Therefore, there are few effective treatment options for obese patients, especially those with obesity related health issues. Drug therapy can serve as a valuable adjunct to lifestyle interventions to achieve and maintain weight loss; it may also reduce metabolic reactions that are beneficial for weight recovery. However, the currently available weight loss drugs are very limited.

The efforts to treat obesity with drugs have been plagued by safety concerns, leading to some drugs not being approved in the United States (such as Rimonabant) or being withdrawn from the US market after approval due to severe toxicity (such as fenfluramine, dexfenfluramine, phenylpropanolamine, and sibutramine). In recent years, new drugs have been approved. As of now, drugs approved by the US Food and Drug Administration (FDA) for the treatment of chronic obesity include olistat, amfepramone/naltrexone, phentermine /topiramate, liraglutide, and semaglutide.

2.2 Background information of this product

GZR18 is a glucagon-like peptide-1 (GLP-1) receptor agonist (RA) under development by Gan & Lee Pharmaceuticals Co., Ltd (hereinafter referred to as Gan & Lee Pharmaceuticals). GLP-1 is secreted by L-cells in the lower part of the small intestine. Studies have shown that GLP-1 has good glucose-lowering and weight-loss effects, whose mechanism of action includes: 1) acting on islet β-cells to activate GLP-1 receptor (GLP-1R), thus promoting insulin synthesis and secretion in a glucose-dependent manner; 2) promoting proliferation and differentiation of islet β-cells and inhibiting apoptosis of islet β-cells to increase the number of islet β-cells;3) acting on islet α-cells and δ-cells to inhibit glucagon secretion; 4) acting on the nervous system to suppress appetite, delay gastric emptying and induce satiety [3].

Given that the majority of subjects with T2DM (over 80%) are overweight, GLP-1 drugs have obvious superiority over other treatments for T2DM due to their concomitant weight loss effects. Since 2014, GLP-1 drugs have been used for weight loss treatment in non-diabetic obese patients.

Natural GLP-1 is readily degraded *in vivo* by dipeptidyl peptidase-4 (DPP-4) and neutral endopeptidase (NEP) with a short half-life of less than 2–3 min [3]. Therefore, one of the important development strategies of GLP-1 therapeutic drugs is to extend their half-lives by modification of their structure.

2.3 Risk/benefit assessment

GZR18 Injection is a human-derived GLP-1 analog. As a long-acting weekly preparation, it is administered subcutaneously once a week (or once two weeks). Compared with once-daily GLP-1 analogs, the low frequency of GZR18 injection alleviates the suffering of patients and improves patients' medication compliance. Non-clinical trials have demonstrated its glucose-lowering and weight loss effects in animals.

With modification to human-derived GLP-1 molecule, GZR18 has clear mechanism of action; the molecular sequence does not contain unnatural amino acids, and thus there may be low immunogenicity; the non-clinical data show that the PK and PD profiles of GZR18 are relatively close to those of Semaglutide, a marketed weekly GLP-1 analog, so the development experience of Semaglutide combined with abundant reference data can be referred to in the clinical development process of GZR18, and thus the overall risk is low.

GLP-1 receptor agonists have glucose-dependent glucose-lowering effect, exhibiting low risk of hypoglycemia and high safety. The clinically common adverse reactions of this kind of drugs are gastrointestinal adverse reactions, mainly including nausea, vomiting, diarrhoea, etc. Similar partial symptoms were also observed in non-clinical studies of GZR18, suggesting that similar adverse reactions may occur in clinical trials of GZR18. The study in all dose groups has been completed in the first human trial and the results suggest that GZR18 has better safety and tolerability than Semaglutide, the best drug of its kind on the market, and it is expected that the severity and incidence of adverse reactions will be lower.

At present, a series of *in vivo* and *in vitro* non-clinical studies of GZR18 have been completed, which fully confirms the high safety and low toxicity of GZR18 in rodents (rats) and non-human primates (cynomolgus monkeys), the efficacy of GZR18 in animal models of diabetes mellitus (db/db mouse model) and animal models for obesity with insulin resistance and prediabetes (ob/ob and DIO mouse models). Partial clinical studies have been completed, indicating that GZR18 has high safety in the human body. These study results provide sufficient theoretical basis and safety assessment for further clinical development of GZR18 injection. It is expected that the clinical benefits of GZR18 outweigh the risks, thus providing a new treatment options for obese/overweight patients.

3. Objectives and endpoints

3.1 Objectives

Primary objective:

- To evaluate the weight loss effect of GZR18 injection in adult obese/overweight subjects after 30 weeks of treatment.

Secondary objectives:

- To evaluate the safety and tolerability of GZR18 injection.
- To evaluate the pharmacokinetic (PK) of GZR18 injection.
- To evaluate the immunogenicity of GZR18 injection.
- To evaluate the effects of GZR18 injection on glucose metabolism, cardiovascular disease risk factors, and patient reported weight related quality of life.

Exploratory objective:

- To evaluate the effects of GZR18 on blood uric acid and fatty liver related indicators.

3.2 Endpoints

Primary efficacy endpoint:

- Percent (%) change from baseline in body weight at the end of the study (W30).

Secondary endpoints:

Efficacy endpoints

- Proportion of subjects who achieved (yes/no) body weight reduction ≥5%, 10%, 15%, 20% from baseline.
- The changes from baseline in body weight, waist circumference, waist to hip ratio (waist/hip circumference), and BMI.
- The percentage change from baseline in body weight (%) after drug withdrawal at the last safe follow-up (W33).
- The changes from baseline in glucose metabolism indicators: HbA1c, FPG, fasting insulin, HOMA-IR and HOMA-β.
- The changes from baseline in cardiovascular disease risk factors: blood pressure (systolic and diastolic blood pressure), pulse, TC, LDL-C, HDL-C, and TG.
- The changes from baseline in the impact of weight on quality of life score reported by patients: the total and individual scores of the IWQOL-Lite-CT, the total and individual scores of the physical and psychological parts of SF-36 scoring table.

Safety endpoints

- The number of AEs that occurred during the study.
- The number of SAEs that occurred during the study.
- The number of AESI that occurred during the study: events of hypoglycaemia, adverse events of gastrointestinal (nausea, vomiting, diarrhoea, and constipation, etc.);
- Vital signs, physical examination, 12-lead electrocardiogram, clinical laboratory test results (hematology, urinalysis, blood biochemistry, blood amylase, blood lipase, coagulation, calcitonin, thyroid-stimulating hormone, free tri-iodothyronine, free tetra-iodothyronine).
- The mental health status of the subjects: to evaluate using the Columbia-suicide severity rating scale and Patient Health Questionnaire.

Immunogenic endpoints

- After administration of GZR18 injection, the changes from baseline in anti-drug antibodies (ADA) to GZR18 and neutralizing antibodies (NAb).

Pharmacokinetics (PK) endpoints

- Subjects with intensive blood sampling: after the last administration, area under the plasma concentration-time curve from time 0 to the time of the last quantifiable concentration (AUC_{last}), area under the plasma concentration-time curve from time 0 to infinity (AUC_{0-inf}), time to maximum observed plasma concentration (T_{max}), elimination rate constant (λz), half-life ($t_{1/2}$), the time point prior to the first observed/measured non-zero plasma concentration (t_{lag}), apparent clearance (CL/F), apparent volume of distribution (Vz/F) and percent of AUC_{0-inf} extrapolated ($AUC_{\%extra}$), mean residence time (MRT), steady-state trough plasma concentration (C_{ss_min}), steady-state peak plasma concentration (C_{ss_max}, the same as C_{max}), average steady state plasma concentration (C_{ss_av}), degree of fluctuation (DF).
- All subjects: steady-state trough plasma concentration (C_{ss_min}).

Exploratory efficacy endpoints

- Blood uric acid change from baseline
- Fatty liver related indicators (the changes from baseline in alanine aminotransferase, aspartate aminotransferase and bile acid).

4. Trial design

4.1 General design

This study is a multicenter, randomized, placebo-controlled phase II clinical study to evaluate the efficacy, safety, tolerability, and pharmacokinetics of GZR18 injection in Chinese adult obese/overweight subjects.

The obese/overweight adult subjects who are inadequately controlled with diet and exercise are included in the study.

Eligible subjects are randomly assigned to four different treatment groups: a group receiving subcutaneous injections of GZR18 solution once every two weeks at three different target doses (12 mg, 18 mg, 24 mg) (or corresponding volume of placebo), a group receiving subcutaneous injections of GZR18 solution once a week at a target dose of 24 mg* (or corresponding volume of placebo), and a newly added group receiving subcutaneous injections of GZR18 solution once every two weeks at a target dose of 48 mg (or corresponding volume of placebo). The specific administration methods are shown in Table 1.

*Note: This group is derived from the modification of the 3.0 version protocol 1-weekly 18 mg group. If subjects from the original 3.0 version protocol 1-weekly 18 mg group do not agree to enter the 1-weekly 24 mg dosing group, the target dose will remain at 18 mg.

All subjects will receive standard dietary and exercise guidance after randomization

4.1.1 Study duration for each subject

- The maximum study period for each subject in this study is approximately 34 weeks: including a screening period of 1 week (Week -1), an administration treatment period/study visit of 30 weeks (Weeks 0 to 30, dose adjustment of weeks 10 to18, stable dosage treatment period of 12 to 20 weeks) + safety follow-up of 3 weeks (Weeks 31 to 33).

4.1.2 Number of planned subjects and research sites

This study plans to enroll 338 adult obese/overweight subjects in approximately 24 research centers in China.

4.2 Justification for selection of doses

This study sets the GZR18 dose groups and titration modalities with reference to GZR18 non-clinical safety support data, clinical trial results obtained to date, and

comparable GLP-1 single-targeted Semaglutide and dual-targeted Tirzepatide.

4.2.1 Summary of the design basis for the trial protocol

This trial protocol refers to its own non-clinical and clinical data, as well as trial designs of similar drugs, and has high feasibility and safety. The specific summary is as follows.

The highest dose setting basis

The conversion of non-clinical toxicological data for GZR18 resulted in a single dose HED of approximately 81 mg~22.8 mg, and a weekly dose HED of approximately 19.44 mg~68.04 mg. The completed clinical trial of GZR18 showed that the subjects tolerated well at a maximum dose of 30 mg.

Existing Phase Ia clinical data indicate that within the dose range of 20 to 50 μg/kg, GZR18 exhibits a dose-proportional-response relationship, indicating that GZR18 dose and exposure can be predicted through a linear relationship, which is also one of the safety foundations for further dose escalation.

The relationship between dose and AE in Phase Ib/IIa Part B showed that AE is mainly concentrated in the early stage of dose escalation (3 to 5 mg). When the dose exceeded 7 mg, the number of AE no longer increased with dose escalation, but showed a significant decreasing trend. This suggests that GZR18 has gradually developed tolerance in the human body after multiple doses, and it is expected that subsequent doses of 24 mg once a week and 48 mg once two weeks may not produce stronger gastrointestinal reactions and other AEs.

Finally, the similar drug Semaglutide is still being re-explored for efficacy at higher doses (7.2 mg and 16 mg) after the 2.4 mg strength for an existing weight-loss indication has been marketed, suggesting that there is a trend in the development of increased doses of GLP-1 analogues to explore superior effects.

In summary, this study is planned to add a 48 mg once every two weeks' target dose group, and if the subjects do not tolerate it, it is allowed to reduce it to 36 mg once every two weeks and continue to be administered. In order to explore the optimal choice of efficacy/safety/compliance for different dosing frequencies in comparison with the 48 mg once every two weeks' group, and to provide more and more sufficient experimental basis for the dosage selection of the subsequent clinical development, the target dose of the once-a-week group is set at 24 mg.

The initial titration dose setting basis

In the phase Ia trial, subjects were given GZR18 at a dose of 50 μg/kg, which was still

well tolerated and safe, and the actual dose of GZR18 administered in this dose group ranged from 2.84 to 4.06 mg/subject, or approximately 3 to 4 mg. Although there was a single pre-adaptive administration of 10 μg/kg prior to the target dose of 50 μg/kg, the design of this dose group could not be used as the basis for the tolerability of a single dose, however, considering that the dose of 10 μg/kg is relatively small and the target dose of 50 μg/kg increases significantly (5 times) compared to it, it is believed that the effect of pre adapted administration of 10 μg/kg can be ignored. 50 μg/kg can be roughly considered as a single dose and well tolerated. Therefore, this study used 3 mg as the starting titration dose.

The dose increase interval setting basis

The increase multiple of the 48 mg dose group in the study is shown in Table 4-1, the amplification factor between doses is maintained at 1.3 to 2 times. The rate of dose escalation in the high-dose group slows down, and it is expected to have certain feasibility.

Table 4-1 48 mg dose-escalation multiplicity of GZR18 injection in phase II (GL-GLP-CH2005)

Number	Dose	Multiples of previous dose
1	3 mg	NA
2	6 mg	2
3	12 mg	2
4	24 mg	2
5	36 mg	1.5
6	48 mg	1.3

Safety and security

This study conducted a comprehensive safety check on the subjects on average once a month, including laboratory tests, ECG tests, physical examinations, etc., to ensure that any potential AEs can be detected in a timely manner during the dose escalation process. And this study has also established strict withdrawal criteria and dosage adjustment strategies, which can be withdrawn from the trial based on safety assessment results or subject wishes. The above measures can maximize the safety and rights of the subjects.

5. Subject population

5.1 Inclusion criteria

Subjects must meet all of the following inclusion criteria to be eligible for the study:

1. Male or female aged 18–65 years (inclusive).
2. Obese subjects (BMI≥28 kg/m^2), Or overweight subjects (24 kg/m^2≤BMI＜28 kg/m^2) with at least one of the following:
   1） Concomitant with one or more of pre-diabetes, hypertension, dyslipidemia, fatty liver (diagnostic criteria can be found in attachments 1 and 2);
   2） Concomitant weight-bearing joint pain;
   3） Obesity-induced dyspnoea or obstructive sleep apnoea syndrome
3. Able to understand the procedures and methods in this study; willing and able to maintain a stable diet and exercise lifestyle during the research period, and willing to sign the ICF voluntarily.

5.2 Exclusion criteria

Subjects will not be enrolled in the study if they meet any of the following criteria:

1. Limb deformities or defects affecting height and body weight measurement.
2. Pregnant or lactating women, men or women of reproductive potential unwilling to use contraception throughout the study and for 6 months after the last dose of investigational drug.
3. History of drug abuse within 1 year before screening, or positive results in drug abuse screening during screening or before randomization.
4. Alcohol abuse history, defined as an average weekly alcohol consumption of more than 14 units for men/7 units for women (1 standard unit equivalent to 360 mL of beer or 150 mL of wine or 45 mL of spirits with 40% alcohol) within 6 months prior to screening.
5. Subjects who are known or suspected to be allergic to GLP-1 drugs or excipients.
6. Subjects with an absolute weight change greater than 5.0% due to any reason within the 3 months prior to screening. The formula for calculating weight change is: (highest weight in the 12 weeks prior to screening - lowest weight)/highest weight * 100%.
7. History or evidence of any of the following:

1) Previous diagnosis of type 1 or type 2 diabetes mellitus;

2) Proliferative retinopathy within 1 year prior to screening or at screening;
3) Previous severe hypoglycemia or recurrent symptomatic hypoglycemia (≥ 2 episodes in half a year);
4) Secondary diseases or drugs leading to obesity, including: elevated cortisol (e.g. Cushing's syndrome), pituitary and hypothalamic damage leading to obesity, weight loss medicine reduction/discontinuation leading to obesity, etc;
5) Previous history of moderate to severe depression; or previous history of severe psychiatric disorders, such as schizophrenia, bipolar disorder, etc. Or when screening, the score of Patient Health Questionnaire (PHQ-9) ≥ 15;
6) Previous suicidal tendency or suicidal behaviour, or when screening, the subjects had suicidal ideation in categories 4 and 5 of the Columbia-Suicide Severity Rating Scale (C-SSRS);
7) Systolic blood pressure ≥ 160 mmHg and/or diastolic blood pressure ≥ 100 mmHg at screening;
8) Previous history of thyroid C-cell carcinoma, multiple endocrine neoplasia (MEN) type 2A or 2B or related family history, or previous history of malignancy within the past 5 years (except for cured basal cell carcinoma of the skin, squamous cell carcinoma of the skin, other polyps and carcinoma in situ of the cervix);
9) History of the following cardiovascular diseases: decompensated cardiac insufficiency (New York Heart Association [NYHA] Class III or IV), unstable angina or myocardial infarction, heart valve replacement surgery, coronary artery bypass grafting (CABG) or other invasive cardiovascular surgery including percutaneous coronary intervention, cerebrovascular accidents or stroke with sequelae;
10) History of acute and chronic pancreatitis, symptomatic gallbladder disease (excluding cholecystectomy), pancreatic injury, and other high-risk factors that may lead to pancreatitis;
11) Subjects with previous gastrointestinal diseases such as gastroparesis, esophageal motility disorders, gastroesophageal reflux, chronic diarrhea, fecal incontinence, and constipation, who are deemed unsuitable for participation in this study by the investigators.

8. Satisfaction of any laboratory test parameter with the following criteria at screening :

1) Serum calcitonin ≥50 ng/L (pg/Ml);
2) Alanine aminotransferase (ALT) ≥ 3.0 × upper limit of normal (ULN) and/or aspartate aminotransferase (AST) ≥ 3.0 × ULN and/or total bilirubin ≥ 2.0 × ULN;
3) Estimated glomerular filtration rate (eGFR) < 60 mL/min/1.73 m^2, estimated by CKD-EPI equation (refer to attachment 3);
4) Presence of thyroid dysfunction (TSH＞6 mIU/L or ＜0.4 mIU/L);
5) Fasting triglycerides ≥5.64 mmol/L (500 mg/Dl);
6) Blood amylase or lipase＞1.5×ULN;
7) International normalized ratio (INR) of prothrombin time > upper limit of normal range;
8) Hemoglobin < 110 g/L (men) or < 100 g/L (women);
9) Fasting venous glucose ≥ 7.0 mmol/L or venous blood glucose after a 2-hour OGTT (75 g) ≥ 11.1 mmol/L;
10) Subjects with positive HIV antibody, treponema pallidum antibody, hepatitis B virus surface antigen (HBsAg) and hepatitis C virus (HCV) antibody.

9. Presence of the following clinically significant 12-lead ECG abnormalities at screening: heart rate<50 beats/min or >100 beats/min, second- or third-degree atrioventricular (AV) block, long QT syndrome or QTc > 500 ms (The calculation formula can be found in Attachment 4), left or right bundle branch block, pre-excitation syndrome, or other significant arrhythmia (other than sinus arrhythmia).
10. Blood donation and/or blood loss ≥ 400 mL or bone marrow donation within 3 months prior to screening, or hematological disorders (including but not limited to haemoglobinopathy, haemolytic anaemia, thalassaemia, sickle cell anaemia).
11. Subjects who had undergone weight loss surgery except acupuncture and moxibustion, liposuction and abdominal liposuction within one year before screening;
12. Previous history of organ transplantation; or medium to major surgery, severe trauma and severe infection within 6 months prior to screening, unsuitable for participation in this study at the discretion of the investigator; or surgery scheduled during the study, except outpatient surgery posing no impact on the safety of subjects and the study results at the discretion of the investigator.
13. Use of any of the following drugs or treatments within 3 months prior to screening:

1) GLP-1 receptor (GLP-1R) agonists or GLP-1R/GCGR agonists or GIPR/GLP-1R agonists or GIPR/GLP-1R/GCGR agonists.
2) Drugs affecting body weight, including systemic steroids (intravenous, oral, or intra-articular administration), Antidepression such as selective serotonin reuptake inhibitors (SSRIs), serotonin noradrenaline reuptake inhibitors (SNRIs), tricyclics, tetracyclics, etc., other psychiatric agents or sedatives (e.g. imipramine, amitriptyline, mirtazapine, paroxetine, phenelzine, chlorpromazine, thioridazine, clozapine, olanzapine, valproic acid, valproic acid derivatives, lithium salts), diuretic, etc.;
3) Traditional Chinese medicine, Chinese patent drug, herbal medicine, health-care products, meal replacements, etc. that affect body weight.
4) Weight loss drugs, such as sibutramine hydrochloride, orlistat, phentermine, phenylpropanolamine, mazindol, phentermine, diethylpropion, lorcaserin, phentermine/topiramate, naltrexone/bupropion, etc.;
5) Hypoglycemic agents, such as metformin, α-glucosidase inhibitors, sulfonylureas, DPP-4 inhibitors, SGLT-2 inhibitors, thiazolidinediones (TZDs), etc.;

14. Participation in clinical trials of other drugs, vaccines or medical devices within 3 months prior to screening and have received treatment.
15. Any other factor that may affect the efficacy or safety evaluation of this study and make the subject unsuitable for participation in this study at the discretion of the investigator.

5.3 Withdrawal criteria

1. Withdrawal at the discretion of the subject.
2. Subject withdrawal required by regulatory authorities.
3. Withdrawal at the discretion of the investigator: It refers to the situation where the enrolled subject is not suitable for further investigational drug treatment during the trial process and the investigator may request the subject to withdraw from the study.
    1) Subjects who do not tolerate any AE before titration up to 12 mg (≤ 12 mg) will withdraw from the trial after evaluation by the investigator.
    2) Subject with other AEs or SAEs that, in the judgment of the investigator, make continuation of investigational drug treatment inappropriate
    3) The subject is diagnosed with moderate to severe depression or other serious mental illness (generalized anxiety, panic attacks, bipolar disorder,

schizophrenia, etc.), or the score of Patient Health Questionnaire (PHQ-9) is ≥ 15 during the trial.

4) Subjects exhibit suicidal tendencies or behaviors, or exhibit suicidal ideation in categories 4 and 5 of the Columbia-Suicide Severity Rating Scale during the trial
5) Pregnancy, planned pregnancy or unwillingness to take effective contraceptive measures;
6) Subjects receiving treatment with GLP-1 receptor agonists and DPP-4 inhibitors;
7) Patients with acute/chronic pancreatitis or high-risk factors of pancreatitis, such as symptomatic gallbladder disease (such as multiple gallstones) and pancreatic injury;
8) Suspected or confirmed thyroid C-cell carcinoma, multiple endocrine neoplasia (MEN) 2A or 2B, or other malignant tumors;
9) New or persistently progressive thyroid-stimulating hormone (TSH > 10 mIU/L) or calcitonin abnormality (calcitonin > 50 pg/mL) that, in the judgment of the investigator, makes continuation of investigational drug treatment inappropriate;
10) Subjects occurring a severe hypoglycemic event (a hypoglycemic event requiring assistance to obtain carbohydrates, glucagon, or other rescue measures) or a recurrent hypoglycemic event (2 or more episodes in 1 month) without an obvious precipitating factor;
11) Subjects with poor compliance, which affects safety and tolerability determinations; including but not limited to:

- Failure to take drugs and undergo tests as required;
- Use of other drugs or foods that interfere with the safety evaluation;
- Rejection by the subject for the dose level or escalation mode to which he/she is assigned to be received;
- Engagement in other behaviors that affect study results

If a subject withdraws from the study, the investigator shall attempt to obtain subject information. For subjects lost to follow-up, the investigator shall make every effort (at least three contacts via telephone with the subject) to complete the final evaluation and document his/her efforts (the date and information summary of the telephone contact shall be included in the source document). All evaluations and

observations, as well as a narrative description of the reasons for subject exclusion, must be documented in the source documents. The eCRF must document the primary reason for withdrawal from the study.

The investigator should contact the CRA as early as possible to discuss subject withdrawal.

5.4 Study termination criteria

Study termination means the termination of all trials before the clinical trial is ended as per the protocol. The purpose of trial termination is mainly to protect the rights and interests of subjects, ensure the quality of the trial and avoid unnecessary economic losses.

1. In case of any serious safety issues during the trial, the investigator shall terminate the trial in a timely manner if he/she deems it necessary to terminate the trial;
2. If the drug is found to have no clinical value during the trial, the trial shall be terminated timely to protect the rights and interests of the subjects and avoid unnecessary economic losses;
3. If major errors in the clinical trial protocol are found in the trial, making it difficult to evaluate the PK/PD profiles and safety of the drug; or significant deviation is made during the implementation of the protocol, which affects the final evaluation of the drug, the trial should be terminated;
4. The sponsor requests termination on the premise of fully safeguarding the safety and rights of the subjects (e.g., funding reasons, management reasons, etc.);
5. The regulatory authority or ethics committee orders the trial to be terminated for some reasons.

5.5 Subject dropouts or lost to follow-up

For subjects who have dropped out, their original medical records should be retained and analyzed：

1. For subjects who have dropped out, study personnel should contact them as much as possible, inquire about the reasons and possible safety; For subjects who dropped out before the first administration of drug, study personnel do not need to undergo follow-up examinations for withdrawal. For subjects who dropped out after administration of drug, study personnel should complete follow-up examinations for withdrawal as much as possible.

2. For subjects who withdrew from the trial due to adverse events, the study personnel should take corresponding measures based on their actual situation, and follow up until the adverse events have improved or returned to normal/abnormal, with no clinical significance or baseline status, or the subjects have been lost to follow-up;
3. The relevant trial data of the dropout subjects should be properly preserved

5.6 Lifestyle concerns

Subjects will maintain a reasonable diet and exercise throughout the trial[5-7]。

The specific dietary and exercise guidance shall be based on the formulated *Dietary and Exercise Management Guidance Manual* for this project.

5.7 Screen failure

Screen failure refers to a subject who has signed the ICF to agree to participate in the study, but is not subsequently randomized, i.e., excluded from the trial according to the inclusion and exclusion criteria at screening or baseline stage. Some information of screen failure should also be documented to ensure that failed subjects can be reported according to uniform standards for clinical trial reporting and to answer the questions from regulatory authorities. Information to be recorded includes: time of screening visit, time of informed consent form signing, demographic data, and reason for screen failure.

If necessary and within the screening window, subjects do not need to generate a new screening number. They can repeat any of the screening assessments/screening procedures once, and will then be judged as a screen failure or not by the investigator based on the results.

The investigators will determine whether secondary screening of the subjects is possible. Secondary screening is only allowed once and should be completed within 4 weeks. The screening number shall be regenerated.

5.8 Recruitment and retention strategy

This trial adopts a combination of in-hospital and out-of-hospital recruitment.

If a subject meets the withdrawal criteria in this protocol, the subject will be withdrawn from the trial.

The reason for withdrawal will be documented for each case. If possible and necessary, subjects who withdraw after administration should have a final visit and complete an end-of-trial form.

6. Study intervention

6.1 Dose and administration

Each subject is to be administered by subcutaneous injection at the site two finger lengths away from the belly button, once every two weeks or once weekly.

Eligible subjects are randomly assigned to four different treatment groups: a group receiving subcutaneous injections of GZR18 solution once every two weeks at three different target doses (12 mg, 18 mg, 24 mg) (or corresponding volume of placebo), a group receiving subcutaneous injections of GZR18 solution once a week at a target dose of 24 mg* (or corresponding volume of placebo), and a newly added group receiving subcutaneous injections of GZR18 solution once every two weeks at a target dose of 48 mg (or corresponding volume of placebo). The specific administration methods are shown in Table 1.

*Note: This group is derived from the modification of the 3.0 version protocol 1-weekly 18 mg group. If subjects from the original 3.0 version protocol 1-weekly 18 mg group do not agree to enter the 1-weekly 24 mg dosing group, the target dose will remain at 18 mg.

Table 6-1 Dosing design

Group	Frequency	Target dose	Dosing design			Subjects	
			Duration (weeks)	Week of Dosing	Dose	GZR18 Injection	Placebo
1	once every two weeks	GZR18-12 mg	2	W0	3 mg	52	13
			4	W2/4	6 mg		
			4	W6/8	9 mg		
			20	W10/12/14/16/18/20/22/24/26/28	12 mg		
2		GZR18-18 mg	2	W0	3 mg	52	13
			4	W2/4	6 mg		
			4	W6/8	9 mg		
			4	W10/12	12 mg		
			16	W14/16/18/20/22/24/26/28	18 mg		
3		GZR18-24 mg	2	W0	3 mg	52	13
			4	W2/4	6 mg		
			4	W6/8	9 mg		
			4	W10/12	12mg		
			4	W14/16	18 mg		
			12	W18/20/22/24/26/28	24mg		
4		GZR18-	2	W0	3 mg	65	13

		48 mg	4	W2/4	6 mg		
			4	W6/8	12 mg		
			4	W10/12	24 mg		
			4	W14/16	36 mg		
			12	W18/20/22/24/26/28	48 mg		
5	once-weekly	GZR18-24 mg（18 mg）*	2	W0/1	3 mg	52	13
			4	W2/3/4/5	6 mg		
			4	W6/7/8/9	9 mg		
			4	W10/11/12/13	12 mg		
			4	W14/15/16/17	15 mg		
			12	W18/19/20/21/22/23/24/25/26/27/28/29	24 mg（18 mg）*		

*Note: If the subjects in the 18 mg once a week group of the original 3.0 version protocol do not agree to enter the 24 mg once a week group, the 18 mg dose will still be administered from W18 to W29.

Dose adjustment standards

The main adverse reaction of GLP-1 drugs is gastrointestinal intolerance. Subjects who do not tolerate any AE before titration up to 12 mg (≤ 12 mg) will withdraw from the trial after evaluation by the investigator.

After titrating from 12 mg to a higher dose, if intolerable, it can be retreated to the previous tolerable titration dose, with a minimum of 12 mg. Other visit and examination times remain unchanged, and the total administration time remains at 30 weeks.

The subjects in the 48 mg dose group (once every two weeks' administration) experience intolerance when titrated from 36 mg to 48 mg. After evaluation by the investigators, they will be allowed to return to 36 mg and continue with once every two weeks' administration. Other visit and examination times remain unchanged, and the total administration duration remaine at 30 weeks.

During the experiment, investigators can also communicate with the sponsor about the dose reduction strategy initiated due to the patient's tolerance and subsequent dose titration strategies based on the patient's situation.

During the protocol change period

If subjects in the 18 mg once a week dose group of the original 3.0 version protocol are willing to enter the 24 mg once a week dose group, the study will be conducted according to the procedure of the 24 mg dose group (Group 5); If subjects are unwilling to enter the once a week 24 mg dose group, a weekly dose of 18 mg will still be administered from Week 18 to Week 29 until the end of the study. The laboratory tests at each visit time point will be consistent with the once a week 24 mg dose group.

6.2 Drug preparation

6.2.1 Drug information

The study drugs used in the study include investigational drugs and placebo. The investigational drug is GZR18 injection, and the placebo is made up of all excipients other than the active ingredient. The detailed information of the experimental drugs is shown in Table 6-2.

Table 6-2 Study drug information

	Investigational drug	Placebo
Name	GZR18 Injection	Placebo (The vehicle of GZR18, which shares same components with GZR18 injection but contains no GZR18. Placebo is similar with the investigational drug in appearance, weight and smell, indistinguishable)
Strength	Please refer to the Certificate of analysis (COA) and injection SOP for details	0 mg/mL
Manufacturer	Gan & Lee Pharmaceuticals Co., Ltd	Gan & Lee Pharmaceuticals Co., Ltd
Administration method	Subcutaneous injection	Subcutaneous injection
Storage conditions	Protect from light, 2°C-8°C	Protect from light, 2°C-8°C

6.2.2 Appearance and packaging

GZR18 and Placebo injections are identical in appearance and are colorless and clear solutions; their packaging is also the same, 3 mL cartridge, aluminum passette, and a small white box from inside to outside.

6.2.3 Label

Labeling of clinical drug supplies will comply with current International Conference on Harmonization (ICH) Good Clinical Practice (GCP) and Good Manufacturing Practice and will include any locally required statements.

6.2.4 Storage and stability of investigational drugs

The investigational drugs are stored in locked cabinets as required and stored according to the standard storage conditions of the drugs. The investigational drugs must be administered according to the process described in this protocol. According to all relevant regulatory requirements, only enrolled subjects are allowed to receive the investigational drug administration. Only authorized research center personnel can distribute investigational drugs. All investigational drugs must be stored in a safe area, and

only investigators and authorized research center personnel can obtain the drugs. The physical storage conditions should meet the relevant requirements of the investigational drugs. The temperature of the storage area for investigational drugs should be 2-8°C. During the storage period of investigational drugs, temperature must be continuously monitored and recorded. Daily maximum and minimum temperatures can be recorded through manual recording or real-time monitoring with automatic thermometers. Once an over temperature situation is found, the inspector or sponsor personnel should be immediately notified to confirm whether the drug can continue to be used

Please refer to the *Drug Administration Manual* for specific requirements.

6.2.5 Drug inventory

Drugs provided for this study may be administered only in accordance with the guidance instructions in the study protocol. Study staff will count all trial medications.

The investigator is responsible for counting, verifying and documenting the trial drug. Throughout the course of the study, the investigator or a dedicated person at the research center must document the counting of trial drugs in accordance with all relevant regulatory requirements. This person will record the number of trial medications received from the sponsor, the number supplied, and/or the number administered and returned by the subjects.

Upon completion of the study and upon receipt of written authorization from the Sponsor, all unused Trial Drugs will be counted and returned to the Sponsor for destruction after completion of the count record.

Please refer to the *Drug Administration Manual* for specific requirements.

6.3 Methods to reduce bias: Randomization and blinding

6.3.1 Randomization

The study is conducted in a placebo-controlled and double-blind manner and a randomization code list is generated using a block randomization procedure with SAS Version 9.4 or above by the statistician independent of this trial. The randomization data is reproducible so that the set parameters such as the seed (initial value) of the random number and block size should be stored. The original randomization list will be preserved by the statistician for the randomization of the project. The randomization list will be imported into the central randomization system.

Subjects will be provided with a unique screening number after signing the ICF. The screening number is in the format of Sxxxxx, where the first two digits of xx represents the site number, the last three digits xxx represents a 3-digit sequence number starting from

001, e.g., S01001 represents the first screened subject at site 01. After completing all assessments and passing screening, the subjects will receive a unique random number, The allocation of random numbers is shown in Table 6-3, and the specific instructions shall prevail. F represents obesity.

Table 6-3 Random number allocation

Number	Dose group	Random number
1	once every two weeks GZR18-12 mg	FA001-065
2	once every two weeks GZR18-18 mg	FB001-065
3	once every two weeks GZR18-24 mg	FC001-065
4	once every two weeks GZR18-48 mg	FG001-078
5	once-weekly GZR18-24 mg (original once-weekly GZR18-18 mg)	FD001-065

The random statistician will generate the project random table. The "block randomization" method is used to generate random numbers using the PLAN process of statistical software SAS 9.4 version or above, the subjects are randomly divided into GZR18 s.c. 12 mg once every two weeks group, GZR18 s.c. 18 mg once every two weeks group, GZR18 s.c. 24 mg once every two weeks group, GZR18 s.c. 24 mg once-weekly group (the original GZR s.c. 18 mg once-weekly group), After that, subjects in each GZR18 dose group are randomly divided into the study group and the control group (placebo) according to the ratio of 4:1. The random table is reproducible, and the seed parameters of the initial value of the set random number need to be saved. According to the version 4.0 protocol, a GZR18 s.c. 48 mg once every two weeks' group is added, and 78 subjects are randomly divided into the study group and the control group (placebo) according to the ratio of 5:1. The random table of the new dose group is also reproducible, and the seed parameters of the initial value of the set random number need to be saved. Randomized subjects who withdraw for any reason, regardless of whether they have used the investigational drug or not, will have their randomization number retained and will not be allowed to re-enter this part of the trial. If there are dropout subjects after randomization, no replacement will be performed.

6.3.2 Blinding

The frequency and dosage of administration in this study are in an open form. In order to reduce bias, this study adopts a double-blind design (i.e., both the investigator and the subjects are blinded) for the subjects who are given GZR18, or placebo within each group. The investigator and the subjects are always blinded to whether GZR18 or placebo is

administered to the subjects throughout the trial, and the safety of GZR18 and placebo is evaluated in a blinded manner, so as to reduce the subjective bias in the study results.

The color of the solution, the smell, the appearance of the packaging (size and color) of GZR18 and placebo will be identical, so that neither the investigator nor the subject will be able to tell whether the subject is receiving GZR18, or placebo.

Kit numbers will be assigned at Gan & Lee Pharmaceuticals or the Clinical Trial Supply Department of the CRO entrusted by Gan & Lee based on the packaging list generated by the validation procedure. This list will be provided to IVRS/IWRS.

Gan & Lee Pharmaceuticals (or designee) will generate the treatment randomization scheme in a manner that will ensure the study team remains blinded according to current Standard Operating Procedure (SOP). The randomization scheme will be kept in a secure place until the unblinding study is conducted for final statistical analysis.

The sponsor, research centers, and staff of Contract Research Organization (CRO) involved in this study are all blinded to the treatment code with the following exceptions:

- ✓ Sponsor's workforce and its subcontractors directly involved in IMP packaging or IVRS/IWRS management.
- ✓ The Pharmacovigilance (PV) staff will be allowed to have access to the IWRS separately to meet their requirements for reporting SAEs to regulatory authorities.

6.3.3 Emergency unblinding

Emergency unblinding might be performed in the event of an emergency situation where it is necessary to know the investigational medical product actually taken by the subject. IWRS is contacted prior to emergency unblinding to determine the group to which the subject is assigned and the dose given. Detailed procedures or training on how to perform code unblinding through the system will be provided to all sites at the start of the study. Whenever possible, CRA or equivalent personnel should be contacted prior to unblinding.

If the code is unblinded, the Clinical Project Manager (CPM) or designee will be notified immediately via the IWRS, but the CPM will remain blinded to specific treatment information. Any unblinding of the IMP by the Investigator must be documented in the source documents and on the study termination eCRF page.

6.4 Intervention compliance

According to the research evaluation schedule 1.2.1, the investigational drug will be administered by the study nurse or the patient his/herself at the time points specified in the protocol.

The study nurse/subjects/their parents/legal representatives must document the subject's medication compliance for each investigational drug administration. For each injection, time compliance is defined as within the dosing time (9:00 a.m.) ± 3 h. If the drug is dispensed to the subjects and injected outside the hospital, the compliance of injection dose shall be additionally recorded: The patient shall take photos of GanleePen with the dose knob adjusted, which will be collected uniformly to record the injection dose. The investigator will instruct the subject/his or her parent/legal representative to bring back at each visit the vial containing all remaining investigational drug dispensed at the previous visit. The acceptable dose compliance is defined as not exceeding the protocol-specified dose ± 10%. Dose compliance is calculated as: The amount of drug injected once into the subject/the total amount of drug to be injected once into the subject ×100%.

If the drug is dispensed to a subject, the investigator will instruct the subject/his or her parent/legal representative to bring back at each visit the vial containing all remaining investigational drug dispensed at the previous visit.

If a subject is found to be persistently non-compliant with treatment (time compliance or dose compliance out of the specified compliance range for 3 times cumulatively), the sponsor will decide with the Investigator whether the subject should be withdrawn from the study.

The quantity of drug dispensed and returned must be recorded in the source documents. Please refer to the drug management manual or injection SOP for specific requirements.

6.5 Concomitant therapy

Any treatment other than the investigational drug, including over-the-counter drugs, must be accurately documented in the source documents and eCRF.

All concomitant medication taken within the three months prior to screening shall be recorded in the eCRF at the screening visit, and thereafter, records shall only be made if there is a change in medication. The corresponding date when any changed administration is initiated shall be recorded in the eCRF.

For all subjects, all changes to their concomitant medications shall be recorded on the pages for concomitant medication of the eCRF, respectively. Such recording shall cover the drug name (generic name, if available, as well as the trade name), indication(s), dosage

form, specification, date of administration, usage and dosage.

The above description also applies to non-drug therapy.

Prior and concomitant medications are listed by subject using WHODrug (current effective version) coding and summarized by the Anatomical Therapeutic Chemical (ATC) classification system.

6.5.1 Prohibited concomitant treatment (drug and non-drug therapies) during the trial

1) GLP-1 receptor (GLP-1R) agonists, GLP-1R/Glucagon Receptor (GCGR) agonists, Glucose-dependent insulinotropic polypeptide receptor (GIPR)/GLP-1R agonists or GIPR/GLP-1R/GCGR agonists;

2) Drugs affecting body weight, including systemic steroids (intravenous, oral, or intra-articular administration), Antidepression such as selective serotonin reuptake inhibitors (SSRIs), serotonin noradrenaline reuptake inhibitors (SNRIs), tricyclics, tetracyclics, etc., other psychiatric agents or sedatives (e.g. imipramine, amitriptyline, mirtazapine, paroxetine, phenelzine, chlorpromazine, thioridazine, clozapine, olanzapine, valproic acid, valproic acid derivatives, lithium salts), diuretic, etc.;

3) Traditional Chinese medicine, Chinese patent drug, herbal medicine, health-care products, meal replacements, etc. that affect body weight;

4) Weight loss drugs, such as sibutramine hydrochloride, orlistat, phentermine, phenylpropanolamine, mazindol, phentermine, diethylpropion, lorcaserin, phentermine/topiramate, naltrexone/bupropion, etc.;

5) Hypoglycemic agents, such as metformin, α-glucosidase inhibitors, sulfonylureas, DPP-4 inhibitors, SGLT-2 inhibitors, thiazolidinediones (TZDs), insulin, insulin analogs, etc.;

6.5.2 Allowable concomitant treatment (drug and non-drug therapies) during the trial

If the investigator judges it necessary to use non-prohibited concomitant treatment (drug and non-drug therapy), the investigator shall truthfully record such information as name, duration, and dosage.

7. Assessments and procedure in the clinical trial

7.1 Safety assessments

Physical examination

Physical examinations will be performed at the time points specified in the study assessment schedule, including: General condition, skin, neck (including thyroid), head, chest, abdomen, back, lymph nodes, the four limbs, and nervous system.

Fundus examination

Fundus examination will be performed at the time points specified in the study assessment schedule. Fundus examination results will be recorded as “normal”, “abnormal but not clinically significant”, or “abnormal and clinically significant”. The “abnormal and clinically significant” will be defined as AE.

Vital signs

Vital signs, including the following, will be measured at the time points specified in the study assessment schedule:

- Blood pressure (mmHg), measured at rest;
- Breath rate (breaths per min);
- Pulse (times per min)
- Body temperature (°C).

Electrocardiogram

Standard resting 12-lead ECGs will be documented for subjects at the time points specified in the study assessment schedule. The results of ECG recordings will be included in the CRF. The source ECG recording will be evaluated by the investigator using automated measurements and the results will be interpreted according to the categories of normal, abnormal but not clinically significant, and abnormal and clinically significant. An ECG is defined as an AE only if it is assessed as abnormal and clinically significant.

Hypoglycaemic episodes and treatment plan

In this study, hypoglycaemia is defined as a blood glucose level below 3.0 mmol/L. Once a hypoglycaemic event is observed, it is graded according to CTCAE 5.0, and classified as either an AE or SAE. The following classification of hypoglycaemia will be used if the blood glucose can be measured:

- Grade 1: < lower limit of normal–55 mg/dL; < lower limit of normal–3.0 mmol/L;
- Grade 2: <55–40 mg/dL; <3.0–2.2 mmol/L;
- Grade 3: <40–30 mg/dL; <2.2–1.7 mmol/L;

- Grade 4: <30 mg/dL; <1.7 mmol/L; life-threatening; epileptic seizure;
- Grade 5: Death.

If the subject experiences symptoms of hypoglycaemia, but his/her blood glucose is not measured or his/her blood glucose ≥ 3.0 mmol/L, investigators need to comprehensively determine whether it is a hypoglycemic event; If it cannot be determined, AE can be recorded separately based on symptoms. The subject who develops a hypoglycaemic reaction may have the following clinical manifestations: palpitations, asthenia, starvation, sweating, pallor and shakiness of hands and feet, etc.; in such a case, the doctor should be informed immediately.

Severe hypoglycaemia is defined if the following criteria are met: hypoglycaemia without specific blood glucose limits, with severe cognitive dysfunction and requiring additional measures to help recover.

Hypoglycemia treatment plan is as follows:

For conscious subjects: The investigator may provide the subjects with carbohydrate food (such as steamed bread, dessert, biscuits, etc.), oral glucose or sucrose, etc. For unconscious subjects: the investigator may establish intravenous access to administrate intravenous glucose as prescribed by the doctor. During the treatment, attention should be paid to the changes of consciousness, speech clarity, body movements and cognitive function. Blood pressure, heart rate, breath rate, and skin color of the subjects should be monitored as well as their blood glucose for any changes to ensure the safety of subjects, and keep them warm. At the same time, medical records and hypoglycemia event reports shall be recorded.

According to the experimental flow chart, the investigator recorded the occurrence of hypoglycemia-related events according to the trial flow chart, if any, in the Hypoglycemic Event Form in the original medical records, and recorded them in the eCRF as well. Information to be recorded includes: Date, time of hypoglycaemic episode, time of last meal before episode, whether there are any symptoms at the time of episode, whether it is self-treatable, and the blood glucose level before treatment (if any).

Hypoglycemia treatment plan is as follows:

For conscious subjects: The investigator may provide the subjects with carbohydrate food (such as steamed bread, dessert, biscuits, etc.), oral glucose or sucrose, etc. For unconscious subjects: The investigator may establish intravenous access to administrate intravenous glucose as prescribed by the doctor. During the treatment, attention should be

paid to the changes of consciousness, speech clarity, body movements and cognitive function. Blood pressure, heart rate, breath rate, and skin color of the subjects should be monitored as well as their blood glucose for any changes to ensure the safety of subjects, and keep them warm. At the same time, medical records and hypoglycemia event reports shall be recorded.

According to the study process table, the investigator should record the occurrence of hypoglycemia-related events, if any, in the Hypoglycemic Event Form in the original medical records. Information to be recorded includes: Date, time of hypoglycaemic episode, time of last meal before episode, whether there are any symptoms at the time of episode, whether it is self-treatable, and the blood glucose level before treatment (if any).

Pancreatitis

If acute or severe & persistent abdominal pain consistent with characteristics of acute pancreatitis occurs, IMP should be interrupted until pancreatitis is ruled out. The investigator may arrange follow-up examinations based on the specific conditions according to national guidelines, expert consensus and diagnosis and treatment specifications such as *Expert Consensus on Emergency Diagnosis and Treatment of Acute Pancreatitis*[8] issued by Chinese Society for Emergency Medicine of Chinese Medical Association, *Guidelines for diagnosis and treatment of acute pancreatitis in China (2021)*[9] issued by Chinese Pancreatic Surgery Association, Chinese Society of Surgery, Chinese Medical Association. If acute pancreatitis is excluded, the subject may be re-enrolled at the discretion of the investigator.

Allergic reactions

In the event of a possible IMP-related severe acute allergic reaction during the study, blood will be drawn for test of trypsin (total and activated) within 3 h following the onset of the allergic reaction. Blood should be drawn for anti-GZR18 IgE antibodies and anti-GZR18 binding antibodies and trypsin after a washout period of at least 5 weeks.

Clinical laboratory tests

Blood will be drawn for hematology, blood biochemistry (fasting), coagulation, lipase, amylase, and TSH/FT3/FT4 at the time points specified in the study assessment schedule. Midstream urine should be collected for urinalysis. The above indexes are all tested in the Department of Clinical Laboratory of the sub-site (hospital).

Blood will be drawn for calcitonin at the time points specified in the study assessment schedule. Based on available data from rodent experiments with other GLP-1 analogs, this class of drugs has a risk of causing thyroid C-cell tumors. But the findings in rodents have

not been observed in non-human primates and are not considered related to humans. However, as a precaution, calcitonin levels in the blood need to be measured during the trial (calcitonin concentrations have been proven to be related with the thyroid function in rodent models). Calcitonin will be tested by the Department of Clinical Laboratory of the sub-site.

Injection site observation

The injection site will be observed for abnormalities at the time points specified in the study assessment schedule, as judged by the subject's chief complaint and the physical examination by the investigator.

Immunogenicity

Blood will be drawn for immunogenicity, including GZR18 anti-drug antibodies (ADA) and neutralizing antibodies (NAb) at the time points specified in the study assessment schedule.

Mental health conditions

Conduct C-SSRS questionnaire and PHQ-9 questionnaire evaluation according to the specified time points in the experimental flowchart, record the mental health status of the subjects, and refer to Attachment 7 and Attachment 8 for detailed questionnaire content.

7.2 Efficacy assessment

Body measurements

Body measurements will be performed at the time points specified in the study assessment schedule, including the height (cm), weight (kg), waist, hip circumference and calculated BMI (kg/m^2) and waist circumference (cm). During measurement, subjects shall with their shoes and coat removed, and shall be fasted with bladder emptied. Please refer to Attachment 5 for specific operations.

Quality of Life Assessment

Conduct SF-36 questionnaire and IWQoL Lite CT questionnaire evaluations according to the specified time points in the experimental flowchart, record the quality of life of the subjects, and refer to Attachment 9 and Attachment 10 for detailed questionnaire content.

7.3 Pharmacokinetic evaluation

One of the secondary objectives of this trial is to assess the pharmacokinetics (PK) of continuous doses of GZR18 by determining the concentration of GZR18 in plasma.

Blood samples will be collected at the time points specified in the study assessment schedule, and the exact time of blood sampling will be documented in the original medical records. PK samples are tested and analyzed by a third-party testing company**.**

7.4 Adverse event

Definition of adverse event

Adverse event (AE) is defined as any untoward medical occurrence in a subject administered the investigational medical product (IMP), which can be manifested as any abnormal sign, symptom, disease, or laboratory finding, and which does not necessarily have to have a causal relationship with investigational drug.

Any untoward medical occurrence from the signing of ICF to the first dose is recorded as medical history. All AEs specified in this protocol that occur after treatment must be completely recorded in the eCRF.

Definition of serious adverse event

Once a subject has been identified as experiencing an AE, the severity of that AE must be assessed. A serious adverse event (SAE) must meet one or more of the following criteria:

1) Death

2) Life threatening

("Life-threatening" means that the subject is at immediate risk of death at the time of the adverse event, that is, it does not include the adverse event that is assumed to cause death in a more serious form.)

3) Leading to persistent or significant disability or incapacity

4) Resulting in congenital anomalies or birth defects (including those developed prenatally)

5) Other important medical events (IMEs). IMEs may not be immediately life threatening, result in death, or lead to hospitalization, etc., but may, in medical and scientific judgment, endanger the patient/subject or may require intervention to prevent any of the above outcomes. Generally, these events shall also be considered as SAEs. Examples of such events include but not limited to: intensive treatment for allergic bronchospasm in the emergency room or at home; cachexia or convulsions that do not cause hospitalization; drug dependence or drug abuse.

6) A need for hospitalization or prolongation of hospitalization (The patient is hospitalized and meet the criteria for requiring hospitalization even if discharged on the same day. Emergency room visits that resulted in hospitalization also meet the criteria for

requiring hospitalization. However, emergency room visits that do not result in hospitalization do not meet this criterion and should be evaluated for one of the other criteria in this definition [e.g., life-threatening adverse event, important medical event]. Hospitalization for reasons unrelated to the occurrence of the AE [e.g., scheduled or elective surgery for a pre-existing condition that does not worsen or present with unusual/atypical manifestations] is not required to be reported. For example, if a subject develops a condition documented in the medical history and subsequently undergoes a previously planned surgery for that condition, it is not appropriate to record the surgery or hospitalization as a serious adverse event because no adverse event occurs that can be assessed against criteria for seriousness. Note that if a pre-existing condition worsens or shows an abnormal or atypical pattern, it is an adverse event and the seriousness of the event will have to be assessed if necessary.)

Adverse events of special interest

Adverse events of special interest (AESIs) mainly includes hypoglycemic events and gastrointestinal intolerance reactions (vomiting, nausea, diarrhoea, abdominal pain, constipation, etc.).

Treatment emergent adverse events

Treatment emergent adverse events (TEAEs): any AE emerging after the first injection of GZR18 or placebo through the end of the safety follow-up period. AEs that occur before dosing of which the severity is increased during the trial are also considered TEAEs.

Definition of suspected unexpected serious adverse reaction

Suspected unexpected serious adverse reactions (SUSARs): the suspected and unexpected serious adverse reactions whose nature and severity of clinical manifestations are beyond the information available in the Investigator's Brochure for the investigational drug, the Instructions for Use of the marketed drug or the summary of product characteristics.

Judgment of severity of AEs

The investigator graded the AEs according to the *Common Terminology Criteria for Adverse Events* (NCI-CTCAE) v5.0. AEs not described in the CTCAE v5.0 are classified according to the following criteria:

Grade 1:	Mild: asymptomatic or mild symptoms; clinical or diagnostic observations only; intervention not indicated.
Grade 2:	Moderate; minimal, local or noninvasive intervention indicated; limiting age appropriate instrumental ADL*.
Grade 3:	Severe or medically significant but not immediately life-threatening; hospitalization or prolongation of hospitalization indicated; disabling; limiting self care ADL**.

Grade 4:	Life-threatening consequences; urgent intervention indicated.
Grade 5:	Death related to AE.

*Instrumental ADL refer to preparing meals, shopping for groceries or clothes, using the telephone, managing money, etc.

**Self care ADL refer to bathing, dressing and undressing, feeding self, using the toilet, taking medications, and not bedridden.

Causality determination of adverse drug reactions

An adverse drug reaction (ADR) is any untoward or unintended reaction possibly related to the investigational drugs in clinical trials. If there is at least a reasonable possibility in the causal relationship between the investigational drugs and the AE, the relatedness cannot be ruled out.

The relatedness between AEs and the investigational drugs are determined according to the following table:

Criteria for judgment of relatedness of AEs to investigational drugs

Definitely related	The AE follows a reasonable temporal sequence from investigational drug administration, and is a known adverse reaction to the investigational drug, which is alleviated or disappears after drug withdrawal, and reappears after drug re-administration, and cannot be reasonably explained by the subject's existing disease.
Probably related	The AE follows a reasonable temporal sequence from investigational drug administration, and is a known reaction to the investigational drug, which is alleviated or disappears after investigational drug withdrawal, and cannot be reasonably explained by the subject's existing disease, with unknown effect after re-administration.
Possibly related	The AE follows a reasonable temporal sequence from investigational drug administration, and is a known or suspected reaction to the investigational drug; however, there may be other factors that may cause the event, such as disease, concomitant medication, etc., and the effect after drug discontinuation is unclear, with unclear information or lack of conclusive information.
Unlikely related	The AE follows a reasonable temporal sequence from investigational drug administration, but the event does not belong to a known type of adverse drug reaction and is most likely caused by the subject's disease or other treatment.
Definitely not related	The AE does not follow a reasonable sequence from investigational drug administration, e.g., the event has occurred before the administration of the investigational drug ; it is not a known adverse drug reaction; or the AE is clearly caused by other factors (e.g., disease of the subject, other treatment, and concomitant medications).

Classification and definition of adverse event outcomes

- Recovered/resolved: The subject is fully recovered, or the condition is recovered to the level at which the subject participated in the first trial-related activity after signing the ICF by medical or surgical treatment;
- Recovering/resolving: The condition is improving and the subject is expected to recover from the event;
- Resolved with sequelae: The subject has recovered from the disease, but there is a persistent effect from the disease, injury, treatment, or surgery. If sequelae meet the criteria for serious, the adverse event must be reported as serious adverse event;
- Not recovered/resolved: The subject's disease has not improved and symptoms have not changed, or the outcome is unknown at the time of reporting;
- Death: This term is only applicable if the subject dies due to a condition associated with the reported AE. The outcome of other AEs reported of the subject prior to death should be assessed as: "recovered", "recovering", "recovered/resolved with sequela", or "not recovered". AEs with fatal outcome must be reported as serious adverse events;
- Unknown: This term is only applicable if the subject is lost to follow-up.

Adverse event reporting

If reporting SAE, the research center must notify Gan & Lee Pharmaceuticals and its

commissioned CRO within 24 hours of receiving this information. Even if the data is incomplete or more data is clearly needed to draw any conclusions, investigators must still properly fill out the SAE report form provided by Gan & Lee Pharmaceuticals (or its authorized CRO) and submit it to Gan & Lee Pharmaceuticals (or its authorized CRO). Gan & Lee Pharmaceuticals (or its authorized CRO) is responsible for entering the information recorded in this table into the global safety database.

The SAE report form will be provided to the investigators. For investigators, it is important to include an assessment of the causal relationship between SAE and the investigational drug when filling out the SAE report form. The evaluation information of investigators is crucial for Gan & Lee Pharmaceuticals to evaluate the safety of IMP and consider whether it is necessary to accelerate the reporting of SAE to regulatory authorities.

Investigators must report any other information received (such as autopsy or laboratory reports) within 24 hours, and the relevant information must be summarized in the SAE report form.

It is explicitly required that investigators collect any SAE events that occur in each subject within 4 weeks after the first and last administration (even if the investigators determine that these events are not related to IMP), and report them to Gan & Lee Pharmaceuticals (or its authorized CRO). Investigators believe that SAEs related to the investigational drug must be reported to Gan & Lee Pharmaceuticals and its authorized CRO, regardless of the time between the event and the end of the study.

After receiving the SAE report form, Gan & Lee Pharmaceuticals and its authorized CRO will evaluate the expected performance of the SAE report. SAE Expectancy Assessment will be conducted based on IB.

Suspected and Unexpected Serious Adverse Reactions (SUSARs) that are fatal or life-threatening should be reported by the sponsor to the drug regulatory authority and the health care authority as soon as they are first notified, and by the sponsor to all the investigators, institutions, and ethical committees participating in the clinical trial as soon as they are aware of it, and the whole process should not take more than 7 days. And the investigators should improve the follow-up information in the subsequent follow-up (the day of the sponsor's first notification is day 0).

Suspected and Unexpected Serious Adverse Reactions (SUSARs) that are not fatal or life-threatening should be reported by the sponsor to the drug regulatory authority and the health care authority as soon as possible within 15 days of first becoming aware of them, and they should be reported by the sponsor to all investigators, institutions, and ethics

committees participating in the clinical trial. And the follow-up information should be improved in the subsequent follow-up (the day of the sponsor's first notification is day 0).

Other AE information will be summarized in the Development Safety Update Report (DSUR) and submitted to regulatory authorities within the time limit specified by relevant regulations along with the DSUR.

**Reporting of serious adverse events (24 h)**	
Email	[redacted]
Reporting process	Complete and sign the SAE Report Form within 24 h after being informed of any SAE and sends the form in PDF format to the sponsor's email [redacted] CRO Pharmacovigilance Public email [redacted]
Mail title	Protocol No.-Site No.+Site name-First/Follow-up#/Summary-Random No.-SAE description-Severity criteria-Relatedness-Date the investigator was informed of this information.<br>E.g. GL-GLP-CH2005-01**Hospital-First-F001-Allergic reaction-Prolonged hospitalization-Unlikely related-20230310
**Reporting of pregnancies (24 h)**	
Email	[redacted]
Reporting process	The investigator completes and signs the Form for Pregnancy Report within 24 h after being informed of any pregnancy and sends the form in PDF format to the sponsor's email [redacted] CRO Pharmacovigilance Public email [redacted]
Mail title	Protocol No.-Site No.+Site name-First/Follow-up#/Summary-Subject No.-Pregnancy-Date the investigator was informed of this information<br>E.g.： GL-GLP-CH2005-01**Hospital-First-F001-Pregnancy-20230310

Adverse event code

Encode all adverse events (severe and non-severe) using MedDRA (current effective version).

Treatment and follow-up of AEs

Any AE occurring during the trial will be treated according to medical practice. All AEs should be followed up until the subject is recovered/resolved, recovering to stable status, recovered/resolved with sequelae, dead, or lost to follow-up.

Any AE continuing in the subject at the end of the study should be followed up until the subject is recovered from the event/recovered with sequelae and the sequelae remain stable, or until the investigator considers the event no longer clinically significant, or until the subject is lost to follow-up. If follow-up is not performed, the investigator must provide justification.

Pregnancy records and follow-up visit

If the investigator finds that the subject became pregnant within 4 weeks after signing the ICF and the last medication, the investigator must fill out a pregnancy event report form

and provide it to the PV department of Gan & Lee Pharmaceutical (or its authorized CRO) within 24 hours. After confirming pregnancy (positive pregnancy test), the subject should withdraw from the study and complete the following activities:

- The subjects should return for early termination (ET) visits.
- The subjects should immediately stop using IMP.
- A safety visit should be planned 2 weeks after the subject terminates IMP treatment.

The investigator must inform the subjects of the current known potential risks and existing alternative treatments.

Pregnancy events should be recorded in the pregnancy event report form provided to the investigator. Pregnancy report forms must be used to track pregnancy progress and final childbirth (when applicable), and investigators must report the health status of the mother and child. Every effort should be made to follow up on the health status of the baby within 30 days after birth to determine if there are any significant medical issues. In some cases, Gan & Lee Pharmaceuticals may require continuous follow-up for more than 30 days. If the subject is lost to follow-up and/or refuses to provide information, the investigator shall provide written records of attempts to contact the subject and keep them on file at the research center. The PV department of Gan & Lee Pharmaceuticals /CRO is the primary contact person responsible for any issues related to pregnancy, final birth, and follow-up data collection.

If a partner of a male subject enrolled in a clinical study becomes pregnant, the investigator or designee is requested to contact the subject and ask the partner of the subject to sign a partner pregnancy consent form approved by relevant IRB/IEC and include this form in the study site file. If there is any objection to the informed consent process, the investigator may contact the CRA of Gan & Lee/CRO. Only after the partner has consented to the collection of additional information and provided a signed partner pregnancy consent form, the pregnancy report form will be completed by the investigator and sent to the PV Department of Gan & Lee/CRO (see SAE reporting information at the beginning of this protocol for contact information). The PV Department of Gan & Lee/CRO is also the primary point of contact for any questions related to the partner pregnancy, final birth and follow-up data collection.

If the following situations occur, pregnancy events are recorded as serious adverse event (SAE): miscarriage, miscarriage (elective or spontaneous), ectopic pregnancy, stillbirth, or any congenital abnormalities/birth defects in the baby. These SAEs must be

reported separately using the SAE report form. Selective induced abortion without medical reasons to terminate pregnancy is not considered an AE/SAE.

Overdose of the investigational drug

Overdose events (exceeding the protocol-prescribed dose, including overdose) shall be recorded in the eCRF. Any other AE/SAE associated with overdose must be followed up as any other AE/SAE. Events are reported as AEs/SAEs (such as a suicide attempt) only if they are accompanied by clinical signs and symptoms or if the overdose itself is an AE/SAE.

Safety signal detection

Selected data from this study will be reviewed periodically to detect any safety concerns related to investigational drug as early as possible so that the Investigator, clinical study subjects, Certificate Authority (CA), and IRB/IEC can be notified appropriately and as early as possible.

The study doctor or medically qualified designee/equivalent personnel will perform ongoing SAE review and ongoing SAE consistency verification in conjunction with the PV representative of Gan & Lee Pharmaceuticals/CRO.

Based on the development stage of the investigational drug and the accumulated experience, other safety assessment measures (e.g.: AEs, vital signs, laboratory or ECG findings) may be determined by medically qualified personnel of Gan & Lee Pharmaceuticals/CRO, and the data will be reviewed periodically during the study.

8. Study Management and Administration

8.1 Protocol compliance

Deviations from the protocol, principles of ICH GCP, or applicable regulations are considered “protocol deviations”. The investigator and all personnel involved in the study should carefully read and strictly follow the protocol. The investigator should not deviate from the protocol. Except in case of emergency, no changes or modifications to the protocol may be made during the execution without the consent of Gan & Lee Pharmaceuticals Co., Ltd. If changes or modifications are required, they must be made in accordance with the specified procedures, and the Ethics Committee (IRB/IEC) must be notified of any change or amendment that may increase the risk of the subject and/or have a negative impact on the rights of the subject and the efficacy of the study, and the written consent of the IRB/IEC must be obtained.

However, standard medical care (prophylactic, diagnostic, and therapeutic procedures) remains the responsibility of the subject’s attending physician (the investigator) in order to protect the health and safety of subjects from any immediate risk. If, in the opinion of the investigator or the qualified professional (associate investigator) authorized by the investigator, the safety of the subject would be jeopardized, any necessary measure that deviate from the protocol or are not specified in the protocol may be taken. In these cases, such measures should be taken immediately without prior notification to regulatory authorities, IEC (Independent Ethics Committee)/IRB (Institutional Review Board), and the sponsor.

Protocol deviations caused by emergencies, accidents, or errors must be reported to the sponsor by the investigator or the designated personnel within 24 hours to facilitate an early decision on whether the subject should be withdrawn from the study.

Any protocol deviations should be documented in detail.

8.2 Monitoring

A risk-based approach is used for quality management of the study. Quality management of the study is initiated by assessing the data and procedures critical to subject protection and the reliability of results, as well as identifying and assessing associated risks. The integrated quality and risk management plan documents the rationale and strategy for risk management during the conduct of the trial, including monitoring methods, supplier management, and other processes that focus on the highest risk areas.

Ongoing risk review and assessment may lead to adjustments to trial implementation, trial design, or monitoring methods.

This study is monitored by Gan & Lee Pharmaceuticals (or designee) for compliance with the sponsor's SOP for monitoring, ICH-GCP guidelines, and applicable regulatory requirements, and to ensure appropriateness of initiation, implementation and termination of the study. Gan & Lee Pharmaceuticals may entrust the study monitoring to CRO or contract CRA.

The investigator and the personnel shall cooperate with Gan & Lee Pharmaceuticals (or designee) and may answer questions sufficiently and provide any missing information during monitoring visits. The investigator/institution allows direct access to source data/documents for study-related monitoring, audits, IRB/IEC reviews, and regulatory inspections.

The investigator allows Gan & Lee Pharmaceuticals (or designee) to periodically review all eCRFs and corresponding source documents (e.g., hospital and laboratory records for each study participant). Monitoring visits provide an opportunity for Gan & Lee Pharmaceuticals (or designee) to evaluate the study progress, verify the accuracy and completeness of the eCRFs, ensure the realization of all protocol requirements, relevant legal requirements, and investigator obligations, and resolve inconsistencies in the study records.

8.2.1 Definition of source data

In accordance with regulatory requirements, the investigator shall prepare and keep adequate and accurate source documents and study records, including all observations and other study data relevant to each study patient. Source data and reported data should follow the "Attributable, Legible, Contemporaneous, Original and Accurate (ALCOA)" principle and should be attributable, legible, contemporaneous, original and accurate. Data changes should be traceable (audit trail).

All source documents must be accurate, legible, permanent, and auditable. Permanent record modes (in ink, printing, CD-ROM) are required. Data are not allowed to be altered with correction fluid or temporary sticky note (e.g., removable self-stick note). Photocopies and/or printouts of the CRF cannot be used as qualified source documents. The source documents are the originals of the raw data first recorded. Such documents include hospital/outpatient/general practitioner records, charts, logs, X-rays, laboratory results, printouts, pharmacy records, nursing records, ECGs or other printouts, completed scales or quality of life questionnaires, etc. Source documents should be kept in a secure site with

restricted access. Original laboratory test results and other test reports are considered source documents and should be stored with the subject's study information.

Printouts of the source documents generated by the computer and saved electronically must be provided for review by the CRA (e.g., ECG reports). The printed copies should be signed and dated by the investigator and kept permanently in the source documents of the subject. The investigator shall simplify the monitoring process and check the printouts with the data stored in the computer to ensure that all data are consistent and correct.

Electronic data records (e.g., continuous glucose monitoring records, if applicable) must be saved and stored as directed by Gan & Lee Pharmaceuticals (or designee).

8.2.2 Source data verification

Source data verification ensures the accuracy and reliability of the study data.

During monitoring visits, the accuracy, integrity, and traceability of the reported data are reviewed against the source documents (e.g., subject documents, records for automatic instrument, tracings, radiographs, records for laboratory tests). All data reported in the eCFR should be supported by the source documents, unless otherwise specified in Section 8.2.1.

8.3 Data processing

8.3.1 Case report form completion

An electronic data capture (EDC) system is used for this study.

The investigator is responsible for timely reporting of accurate, complete and clear data in the eCRF and all specified reports. Justification is required for any change or correction made after saving of eCRF. Corrections made after review and approval by the investigator (via password/electronic signature) are reapproved by the investigator. The investigator should maintain a list of personnel authorized to enter data into the eCRF.

The data on the eCRF must be consistent with the source data. The inconsistencies must be explained.

The current medical history of the subject may not be sufficient to confirm his/her eligibility for this study. So the investigator may need to ask for evidence of past medical history and any diagnostic tests. In such case, the investigator must make at least one attempt to retrieve the previous medical records and document it. In case of failure, oral medical history provided by the patient may be accepted (if applicable). The investigator shall determine the subject's eligibility for this study based on all available information and document it in the source documents.

If the subject does not comply with the protocol, any corrective actions (e.g., retraining) must be documented in the subject file.

For eCRFs, data must be derived from source documents such as:

- Subject identification: Sex, year of birth (according to local laws and regulations)
- Participation of the subject in the study (drug, study protocol number, patient ID, date of informed consent of the patient)
- Date of visit of the subject
- Medical history (including trial indication and concomitant diseases, if applicable)
- AE (onset date (mandatory) and end date (if applicable))
- SAE (onset date (mandatory) and end date (if applicable))
- Concomitant therapy (start date, changes)
- Originals or copies of laboratory results and other imaging or test results, and appropriately documented medical evaluations (in validated electronic format, if applicable)
- Trial completion of the subject (end date; reasons for early termination should be recorded).
- Evidence that the study participant meets all inclusion criteria and none of the exclusion criteria must be documented in source data (e.g. medical records) before he/she is assigned to the clinical study. Lack of documentation to support inclusion/exclusion criteria (whether medical records or verbal record feedback of the patient, or protocol-specified examinations) may result in disqualification of the subject from participating in the clinical study.

Refer to the filling guide of eCRF for detailed instructions.

8.3.2 Database entry and consistency verification

It is the primary responsibility of the investigator to ensure that the data reported on the electronic case report form or otherwise are accurate, complete, and timely, and it should be ensured that the data on the electronic case report form are derived from the original data from the subject and an explanation must be given for any differences.

Clinical research associates (CRAs) are responsible for source data verification (SDV), logging on to the EDC at the site of the research center to verify the consistency of the eCRF data with the source data, and can issue a query online at any time if they find a problem. After the data are filled into the EDC database, the system will automatically verify the data according to the system logic verification that has been set up, and system queries will be issued automatically for problematic data. The Data Manager (DM)

manually verifies the data according to the DVP, and sends manual queries to the EDC database for problematic data.

Challenges issued by the CRA SDV & DM Verification should be answered as soon as possible by the investigator or the clinical research coordinator (CRC) designated and trained by the investigator, and the CRAs and DMs confirm their own queries on the basis of the investigator's answers, and if necessary, may reissue the challenge until all data queries have been resolved.

The investigator or CRC can modify the data after verifying the data, the modified data need to fill in the reason for modification on the eCRF and leave an audit trail in the database, and the investigator needs to audit all the final data. After all the data in the database have been verified and cleaned without doubt by CRA and DM, the principal investigator confirms the authenticity and integrity of the data and signs it electronically.

8.3.3 Subject screening and enrollment log/subject identification code list

Screening information and enrollment information of the subject is recorded in the subject screening and enrollment log.

The investigator keeps a subject identification code list. This list is kept by the investigator for accurate identification of each subject.

The subject’s consent to participate in the study and enrollment information must be recorded in the his/her medical records. These data should be labeled “For use in this study only”. The date on which the subject participates in the study should be indicated.

8.4 Study termination

Gan & Lee Pharmaceuticals reserves the right to suspend or permanently terminate the study in a single site, multiple sites or all sites at any time for reasons including, but not limited to, safety or ethical issues, incorrect or incomplete data recording, noncompliance, or unsatisfactory quality or quantity of patients recruited.

If the study is early terminated or suspended, Gan & Lee Pharmaceuticals (or its representative) will notify the investigator/study sites and regulatory authorities as required by the appropriate regulatory authorities and provide the reasons for termination or suspension of the study. The sponsor or the investigator/study site should also notify the IRB/IEC and explain the reasons for termination or suspension of the study, as required by the appropriate regulatory authorities. In addition, all unused IMP and other materials will be returned at scheduled times according to the study procedures of Gan & Lee Pharmaceuticals.

8.5 Archiving and data preservation

Source documents and other important GCP-specified documents (including ISF) must be kept by study sites and the record keepers of IRB/IEC according to the protocol or for a locally required period of validity at the end of the study (whichever is longer). If Gan & Lee Pharmaceuticals requests to extend the retention period of these documents, the retention period and method will be determined after discussion between Gan & Lee Pharmaceuticals and study sites.

Gan & Lee Pharmaceuticals (or designee) is responsible for informing the record holder when these documents will no longer be retained.

Gan & Lee Pharmaceuticals and its CRO must retain the necessary documents in accordance with their SOPs and contracts.

8.6 Audit and inspection

Upon rational notification, the investigators or the head of the research center involved in this study will allow Gan & Lee Pharmaceuticals to enforce study-related audits and allow inspections by domestic and foreign regulatory authorities.

The primary objective of the audits or inspections is to ensure that the rights and health of enrolled subjects are safeguarded, that the enrolled subjects (i.e., subjects who sign the consent and undergo study procedures) are suitable for participation in this study, and that all relevant data for IMP evaluation have been processed and reported in accordance with established arrangements, the study protocol, SOPs for research center and IRB/IEC, ICH GCP, and relevant regulatory requirements.

If an inspection is notified by a regulatory authority, the investigator should promptly notify Gan & Lee Pharmaceuticals (or designee).

At the time of study-related surveillance, audits, IRB/IEC reviews and regulatory inspections, the investigator must provide direct access to eCRFs and all source documents/data (including copies of progress notes, laboratory results and medical examination results), and must be readily available for review by CRA, auditors, and regulatory inspectors (e.g., Medical Products Administration). They can review all eCRFs and ICFs. The accuracy of the data is verified by direct comparison with the source documents described in Section 8.2.1.

The sponsor and its CRO are also monitor compliance with the protocol and GCP.

8.7 Good Clinical Practice

For noncompliance with the protocol, ICH-GCP, or requirements of local regulatory authority by the investigator, study sites, their personnel, or designee of the sponsor, Gan & Lee Pharmaceuticals will take timely measures to ensure compliance. Persistent noncompliance may result in termination of study activities at relevant sites.

8.8 Biological samples

Biological samples for this study will be stored in accordance with national laws and regulations. Gan & Lee Pharmaceuticals and its partners can only use biological samples for the purposes stated in the protocol and ICF. Subjects have the right to request the destruction of their biological samples at any time.

Detailed information on collection, processing, storage, and shipment of biological samples are shown in the sample handling manual or corresponding documents.

9. Statistics

The statistical methods are described below.

9.1 Definition of analysis sets

Full Analysis Set (FAS): FAS includes all randomized subjects who received at least one dose of the investigational drug. The data set is the main analysis set of demographic data, baseline indicators, medical history and curative effect.

Per Protocol Set (PPS): PPS is a subset of FAS, which includes subjects who met all inclusion criteria, did not meet exclusion criteria, had primary endpoint efficacy indicators and did not have other major protocol deviations. The data set is used for the supportive analysis of efficacy.

Safety Set (SS): SS includes all randomized subjects who received at least one dose of investigational drug and had at least one safety evaluation. This data set is used for statistical analysis of safety indicators.

PK Concentration Set (PKCS): PKCS includes all randomized subjects who received at least one dose of investigational drug and had at least one measured plasma concentration of the investigational drug available after medication.

PK Parameter Set (PKPS): PKPS includes all randomized subjects who received at least one dose of the investigational drug and had at least one valid PK parameter of the investigational drug during the study.

Other

All efficacy analyses are performed primarily in FAS. To verify the robustness of the efficacy results, the primary efficacy variable is also analyzed for PPS. Safety analysis is conducted in SS.

All efficacy analyses are based on randomized treatment assignment, Safe analysis based on actual groups. Subjects receiving incorrect treatment are evaluated during a blinded Data Evaluation Meeting (DEM) to assess the potential impact of such cases and any special analytical considerations.

9.2 General statistical consideration

Descriptive statistics such as mean, standard deviation (SD), median, lower quartile (Q1), upper quartile (Q3), minimum and maximum for quantitative variables, and counts and percentages for categorical variables are provided. Critical supporting data are provided in data listings. Unless otherwise stated, all statistical tests (95% CIs) will be two-

sided at a significance level of 0.05, and calculate the corresponding 95% confidence interval (CI). A statistical outlier is defined as a value that is inconsistent with other values and is clinically unreliable. Removal of outliers from analyses requires a thorough justification based on statistical and clinical background. Any outliers are to be reviewed during the blinded EDM (see Section 9.5). All statistical analyses will use SAS 9.4 version or above.

9.2.1 Demographic characteristics of subjects

Age (years), sex, ethnicity, height (cm), body weight (kg), waist circumference (cm), and BMI (kg/m^2). Descriptive statistical method is used to list the demographic data, including distribution of analysis sets of subjects, listings of datasets of subjects, subjects who violate the protocol and their reasons.

The demographic characteristics and other baseline characteristics are analyzed descriptively based on FAS. Continuous variables are described by number of subjects, mean, standard deviation, median, lower quartile (Q1), upper quartile (Q3), minimum and maximum, and calculate frequency number and frequency of counting and grading data.

9.2.2 Compliance and statistical analysis of concomitant medication

Compliance is defined in Section 6.4.

Compliance is analyzed based on FAS. Concomitant medication is described in detail in a tabular format.

9.3 Planned safety analysis

Safety endpoints are summarized using descriptive statistics based on SS. The number, number of subjects and incidences of various AEs and adverse reactions (including all AEs, TEAE/TRAE, AEs/adverse reactions of special interest, AEs/adverse reactions related to hypoglycemic events, AEs/adverse reactions of Grade III or above, SAEs/serious adverse reactions, AEs/adverse reactions leading to dose suspension, AEs/adverse reactions leading to study withdrawal) are summarized.

AEs are coded according to the Medical Dictionary for Regulatory Activities (MedDRA®). AE data listings, including preferred term (PT), system organ class (SOC), treatment, severity, and relationship to investigational drug s. The number of subjects with AEs and number of AEs are summarized by SOC and PT. AEs are summarized by severity and relationship to investigational drugs.

Laboratory evaluations, vital sign assessments, and ECG parameters are summarized at protocol-specified collection time points. Changes from baseline are summarized at each

protocol-specified time point. Changes of each clinical assessment parameter in clinical significance from baseline were cross-tabulated at each visit.

Concomitant medications are listed by subject, coded using the World Health Organization Drug Dictionary (WHODrug), and summarized by ATC.

Immunogenicity analysis:

The proportion of ADA-positive subjects and NAb-positive subjects in each group are summarized, and listings of ADA-positive subjects and NAb-positive subjects are provided. Samples from positive subjects will be further investigated in vitro if necessary, and additional detailed analysis reports of ADA and NAb will be provided separately.

9.4 Scheduled efficacy analysis

hypothesis-testing:

	Percentage change in body weight from baseline to W30 (one-sided α=0.025)
Null hypothesis	H_0: $\mu_T - \mu_C \geqslant 0$
Alternative hypothesis	H_1: $\mu_T - \mu_C < 0$

μ_T is the mean percentage change of body weight from baseline in the first to fifth dose groups after 30 weeks of GZR18 treatment (μ_T includes μ_{T1}, μ_{T2}, μ_{T3}, μ_{T4} and μ_{T5}, which corresponds to GZR18 s.c. 12 mg once every two weeks , GZR18 s.c. 18 mg once every two weeks , GZR18 s.c. 24 mg once every two weeks , GZR18 s.c. 48 mg once every two weeks and GZR18 s.c. 24 mg once-weekly respectively), μ_C is the mean percentage change of body weight from baseline in the first to fifth dose groups after 30 weeks of placebo treatment. α is significance level (Type I error rate).

Based on FAS, conduct effectiveness analysis on efficacy indicators. PPS as a supportive analysis result.

Primary endpoint analysis: the percentage of body weight change (%) from baseline at week 30 will be taken as the primary endpoint, which will be analyzed based on the covariance model (ANCOVA), where, with baseline body weight as the covariate and group as the fixed effect, and the change from baseline is the dependent variable. The following indicators will be then calculated: the least-squares mean (LSMean) and its two-sided 95% CI of the percentage of the change from baseline in both groups, as well as the LSMean difference between the treatment and control groups and its two-sided 95% CI.

Secondary endpoint analysis: The continuity variables will be analyzed by the same method as the primary endpoint analysis. The number of cases reaching the target end point

and the rate of reaching the target and its two-sided 95% CI will be counted in the analysis of the proportion between the two groups. At the same time, the rate difference between the two groups (the investigational drug group and the placebo group) and its two-sided 95% CI will be calculated. The rate of reaching the target and the two-sided 95% CI of the rate difference between the two groups will be calculated by the Clopper-Pearson Exact method.

Sensitivity analysis

Sensitivity analysis of main efficacy indicators. The Mixed Model Repeated Measures (MMRM) method will be used to incorporate all time points from baseline to week 30 into the Linear Mixed model (LMM) as a supporting analysis to compare the efficacy differences between the treatment group and the placebo group. Consistent with the primary endpoint analysis method, some specific populations are excluded for comparison of efficacy between groups.

9.5 Planned PK analysis

Drug concentration data will be summarized based on the PKCS analysis population. The analysis results of trough concentrations will be summarized by dose groups using the number of subjects, arithmetic mean, standard deviation, coefficient of variation (CV), median, quartile, minimum, maximum, and geometric mean. Mean blood concentration-time plots (raw and semi-log transformed values) will be plotted according to the planned time of blood sampling, and individual blood concentration-time plots (raw and semi-log transformed values) will be plotted according to the actual time of blood sampling.

Pharmacogenetic parameters will be calculated for each subject based on PKPS using the non-atrial model in Phoenix WinNonlin 8.3 (Certara LP, Princeton, NJ, USA) software or above. The arithmetic mean, standard deviation, coefficient of variation, median, quartiles, minimum, maximum, geometric mean and geometric coefficient of variation will be also calculated for each parameter. The Power model will be used to explore the linear characterization of pharmacokinetic parameters with respect to dose when data were sufficient.

9.6 Handling of protocol deviations

A blinded DEM will be held prior to unblinding of the clinical database and the study after all eCRFs are retrieved and entered and the queries are resolved. The objectives of the blinded DEM are to assess the quality of data from subsequent database locks, identify protocol deviations, identify analysis populations, agree on summary of statistical analysis

stratification levels, and verify the appropriateness of statistical hypothesis for the primary analysis.

9.7 Handling of dropouts or missing data

Subjects who provide at least one efficacy variable data during the treatment period are included in the primary analysis. If a subject early terminates the study prior to the end of the treatment period, his/her last efficacy variable data will be collected for analysis.

9.8 Determination of sample size

Based on the study results of similar drugs and the non-clinical data of GZR18 and the existing clinical human pharmacodynamic data of GZR18, it is estimated that the difference of the percentage of body weight change from baseline at W30 between GZR 18 and placebo group is 11% (GZR18 weight loss of 13%, placebo group weight loss of 2%), the combined standard deviation is 10%, the degree of grasp is 0.8, one-side α 0.025, GZR18 group: placebo group: 4:1, then the calculated sample size is 40 subjects for GZR18 investigational drug and 10 subjects for placebo. Considering the 20% dropout rate, 52 subjects receive GZR18 investigational drug and 13 subjects receive placebo actually. There are five dose groups of GZR18, including the 12 mg/18 mg/24 mg once every two weeks' group and the 24 mg (18 mg) once-weekly group, with 52 subjects in each investigational drug group and 13 subjects in the placebo group; The subjects in the 48 mg once every two weeks' group may not titrate 48 mg due to intolerance to AE, so the number of subjects in the GZR18 group is increased to 78 (65 subjects for the GZR18 investigational drug and 13 people for the placebo group). The total sample size is 338 subjects.

The number of people to be enrolled will be determined based on the assessment of data from nonclinical studies of GZR18 and some data from the clinical trial of GZR18, as well as the number of Phase II clinical trials of similar drugs under development or already marketed, and information considered sufficient by the investigator and sponsor to achieve the trial objectives.

9.9 Statistical software

Unless otherwise specified, SAS 9.4 version or above will be used for all statistical analyses.

10. Regulation, Ethics, and Study Regulatory Requirements

10.1 Regulation

This study is conducted in accordance with the protocol, the ethical principles of the Declaration of Helsinki, ICH GCP harmonised guidelines, SOPs for Gan & Lee or its CRO, and other relevant regulations. The investigator and staffs of research centers must adhere to these principles.

In accordance with national and international regulations, this study may only be initiated after the required regulatory documents have been reviewed and approved by the Institutional Review Board (IRB)/Independent Ethics Committee (IEC) and the Certificate Authority (CA). The same process is also required for the implementation of the revised changes.

10.2 Informed consent

In accordance with local regulations, ICH-GCP requirements, and ethical principles derived from the principles of the Declaration of Helsinki, informed consent must be obtained and documented prior to the patient's participation in the study.

Each signature of the subject (or his/her legal representative) on the written ICF must be personally dated by the signer. The ICF and any other notices for subjects are kept by the investigator as part of the trial records. Each subject or his/her legal representative must be provided with a signed copy of the ICF and any other notices for patients.

Prior to obtaining informed consent, the investigator (or designee) should provide study information verbally and in writing to the subject or his/her legal representative in a language and at a level of complexity that is understandable to the subject or his/her legal representative. Adequate time must be given to the subject or his/her legal representative to consider participation in the study. Each subject or his/her legal representative have the opportunity to discuss the study and its alternatives with the investigator.

An ICF is personally signed and dated by the subject or his/her legal representative and the person responsible for the informed consent (the investigator or designee) prior to participation in the study. A trial partner providing a supplementary explanation should also sign (or stamp) and date the ICF. The subject or his/her legal representative should be provided with a signed and dated copy of the ICF.

In addition, the subject will sign an IRB/IEC-approved consent form, as applicable (based on subject age and local requirements). In the informed consent process, each subject must agree to have direct access to his/her medical record for study-related monitoring, audits, IRB/IEC review, and regulatory inspection.

If the ICF is amended during the study, the investigator (or the sponsor, as applicable) must comply with all relevant regulatory requirements for the revised ICF approved by the IRB/IEC and use the revised ICF.

When additional relevant information becomes available, a second informed consent procedure may be required. The procedure shall be performed as directed by the sponsor.

The processes of informed consent and re-consent should be properly documented in the source documents.

10.3 Subject ID card

After signing ICF and consent form (if applicable), subjects or their legal representatives are provided with the subject ID card in their native languages. The investigator fills out the ID card with subject identification information and medical emergency contact information, and instruct subjects to carry the card with them at all times.

10.4 Institutional Review Board (IRB) and Independent Ethics Committee (IEC)

The study is conducted with the support of the IRB/IEC in accordance with local regulations, ICH-GCP and the ethical principles of the Declaration of Helsinki.

The investigator/Gan & Lee Pharmaceuticals will ensure that an IRB/IEC appropriately constituted in accordance with current ICH-GCP or relevant country-specific regulatory requirements is responsible for the initial and ongoing review and approval of the clinical study. Prior to start of the study, the investigator/Gan & Lee Pharmaceuticals will submit copies of the protocol, ICF, Investigator's Brochure, the investigator's curriculum vitae (as applicable), advertisements (as applicable), and all other subject-related documents to the IRB/IEC for review and approval.

The investigator will obtain written full approval of the signature and date of the protocol by the responsible IRB/IEC prior to the start of the study.

The investigator will also promptly report to the IRB/IEC all protocol changes in the study, all unexpected issues that pose a risk to human subjects or others, and any protocol deviations, so as to eliminate a direct risk to subjects.

The investigator should not make any changes to the conduct of the study during the study without the approval of the IRB/IEC, unless the changes are necessary to eliminate apparent direct hazards to subjects. For minor modifications made to the previously approved protocol during the period covered by the original approval, the investigator may obtain expedited review as permitted by the IRB/IEC.

As part of the IRB/IEC requirements for continuing review of approved studies, the investigator will be responsible for submitting periodic progress reports (as required by the IRB/IEC) to the IRB/IEC at intervals appropriate to the degree of risk to subjects involved, with annual review times based on the requirements of each participating hospital. The investigator should provide the final report to the IRB/IEC after the end of the study.

Gan & Lee Pharmaceuticals (or its representative) will provide safety information to relevant regulatory authorities and all investigators conducting the study in accordance with relevant regulatory requirements. The investigator or the sponsor will also notify the relevant IRB/IEC in accordance with the respective regulatory requirements of each relevant country. The investigator should provide evidence of these IRB/IEC notifications to the sponsor (or its representative), as applicable.

10.5 Privacy of subjects

Personnel (or designee) of Gan & Lee Pharmaceuticals will ensure and maintain the confidentiality of the subject's identity. All data provided to Gan & Lee Pharmaceuticals (or designee) throughout the study will be identified only by the random number assigned at screening. With the consent of the investigator, representatives of Gan & Lee Pharmaceuticals, designees, relevant IRB/IEC representatives, or representatives of regulatory authorities will be allowed to review portions of the subject's primary medical records that are directly related to the study (including but not limited to laboratory tests, test result reports, ECG reports, hospitalization/discharge summaries of the subject during participation in the study, and autopsy reports of deaths during the study).

10.6 Protocol amendment

Protocol changes may affect the legal and ethical status of the study, and may also affect the statistical evaluation of the sample size and the likelihood that this study will achieve its primary objectives.

Major changes to the protocol will be made only as protocol amendments and must be approved by Gan & Lee Pharmaceuticals, IRB/IEC and regulatory authorities (if required) prior to implementation.

11. Finance, Insurance and Publication

The rights of the investigator and the sponsor to publish the results of this study, as well as the financial provisions, are described in the study contract. Typically, study results should not be published prior to finalization of the clinical study report.

The program will provide/purchase clinical trial insurance.

12. References

[1]. Yang Wenying, Lu Juming, Weng Jiangping, et.al. Prevalence of diabetes among men and women in China [J]. N Engl J Med. 2010, 362(25):2425-2426.

[2]. Drucker, D.J. Mechanisms of Action and Therapeutic Application of Glucagon-like Peptide-1. Cell metabolism. 2018, 27(4):740-756.

[3]. Meier, J.J. GLP-1 receptor agonists for individualized treatment of type 2 diabetes mellitus. Nature reviews Endocrinology. 2012, 8(12):728-742.

[4]. American Diabetes Association. STANDARDS OF MEDICAL CARE IN DIABETES, 2022.

[5]. Writing committee of expert consensus on overweight/obesity medical nutrition therapy in China. Expert consensus on overweight/obesity medical nutrition therapy in China[J]. Chinese Journal of DiabetesMellitus, 2016,8(9):525-540.

[6]. Guideline for the Prevention and Treatment of Type 2 Diabetes Mellitus in China (2020 Edition) [J]. Chinese Journal of Diabetes Mellitus,2021, 13(4):315-409.

[7]. Nutrition and Metabolic Management Branch of China International Exchange and Promotive Association for Medical and Health Care, Clinical Nutrition Branch of Chinese Nutrition Society, Chinese Diabetes Society, etc. Guidelines for medical nutrition treatment of overweight/obesity in China (2021) [J]. Chinese Journal of the Frontiers of Medical Science. 2021, 13(11):1-55.

[8]. Chinese Society for Emergency Medicine of Chinese Medical Association. Expert Consensus on Emergency Diagnosis and Treatment of Acute Pancreatitis [J]. Journal of Clinical Hepatology. 2021, 37(5):1034-1041.

[9]. Chinese Pancreatic Surgery Association, Chinese Society of Surgery, Chinese Medical Association. Guidelines for Diagnosis and Treatment of Acute Pancreatitis in China (2021) [J]. Chinese Journal of Surgery. 2021, 59(7): 578-587.
